# Uncovering the post-pandemic timing of influenza, RSV, and COVID-19 driving seasonal influenza-like illness in the United States: a retrospective ecological study

**DOI:** 10.1016/j.lana.2025.101359

**Published:** 2026-01-01

**Authors:** George Dewey, Austin G. Meyer, Raul Garrido Garcia, Mauricio Santillana

**Affiliations:** a Machine Intelligence Group for the Betterment of Health and the Environment, Northeastern University, Boston, MA, 02115, USA; b Network Science Institute, Northeastern University, Boston, MA, 02115, USA; c Department of Physics, Northeastern University, Boston, MA, 02115, USA; d Baylor Scott and White Health, Dallas, TX, 75426, USA; e Department of Epidemiology, Harvard T.H. Chan School of Public Health, Boston, MA, 02115, USA

**Keywords:** Disease surveillance, RSV, Influenza, Influenza-like illness, Forecasting, Pandemic preparedness

## Abstract

**Background:**

Influenza and respiratory syncytial virus (RSV) are major contributors to the burden of seasonal influenza-like illnesses (ILI) in the US. The prevention and treatment of ILI varies substantially across age groups and in cost and administration schedule. This study aimed to characterize the timing and ordering of RSV, influenza, and COVID-19 epidemics in the post-pandemic period to inform public health preparedness.

**Methods:**

We implemented a series of independent regression models to infer the contribution of each of these diseases to seasonal ILI syndromic indicators. We further implemented anomaly-detection algorithms on data from the US Centers for Disease Control and Prevention National Syndromic Surveillance Program for the 2022–23, 2023–24, and 2024–25 ILI seasons to identify the timing of onsets and peaks of RSV, influenza, and COVID-19.

**Findings:**

A total of 148 state-ILI seasons were analyzed. In 114 out of 148 (77.0%) of analyzed seasons, volume of RSV emergency department (ED) visits peaked before influenza ED visits. The median time difference between peaks of RSV and peaks of influenza was +3.0 weeks (95% percentile range: −7.0, +7.0 weeks; interquartile range: 5.0 weeks). The timing of RSV and influenza onsets were found to occur more synchronously in the 2023–2024 and 2024–2025 ILI seasons. The timing of COVID-19 outbreaks did not show a consistent seasonal pattern across the study period.

**Interpretation:**

RSV epidemics frequently reach peak volume before influenza epidemics across the US. Healthcare professionals and public health authorities should anticipate increases in RSV cases and hospitalizations at the start of the annual ILI season and establish infrastructure and planning to handle incoming surges of both RSV and influenza appropriately.

**Funding:**

CDC 10.13039/100025287Center for Forecasting and Outbreak Analytics; 10.13039/100000002National Institutes of Health.

**Research in context**

**Evidence before this study**

The COVID-19 pandemic significantly disrupted seasonal patterns of epidemics of RSV, influenza, and COVID-19, resulting in uncertainty about the relative timing of outbreaks of these respiratory diseases. In the United States (US), the timing of RSV, influenza, and COVID-19 epidemics has been captured by influenza-like illness (ILI) surveillance. We conducted searches of PubMed from November 11 to November 19, 2025, using the search terms “timing” and “influenza” and “RSV”; “ordering” and “influenza” and “RSV”; or “sequence” and “influenza” and “RSV” for studies published after March 2020 with no restriction for language. While several recent studies noted changes in RSV and influenza seasonality post-pandemic, these studies focused predominantly on non-US regions; none of these studies used recent, near-real-time national syndromic surveillance data to quantitatively decompose the ILI signal and define the precise ordering of RSV, influenza, and COVID-19 across the US.

**Added value of this study**

This study provides a robust, state-by-state analysis of the three complete ILI seasons immediately following the pandemic (2022–2025). Using a regression-based approach on national syndromic data, we quantify the relative contribution of each virus to the seasonal ILI burden. We show that in 114 of 148 state-ILI seasons from between 2022 and 2025, peak volume of RSV emergency department visits peaked before influenza ED visits. We also find that the median time difference between peaks of RSV and peaks of influenza was +3.0 weeks, while the timing of RSV and influenza onsets were found to occur more synchronously in 2023–24 and 2024–25.

**Implications of all the available evidence**

Influenza, RSV, and COVID-19 epidemics stress health facilities and the general population due to variations in morbidity and mortality, type and cost of treatment, and wide-ranging effects across many age groups. Infants and individuals aged 65 and older are at higher risk of substantial symptoms such as lower respiratory infection because of these viral infections. Improved understanding of the timing and sequencing of these epidemics during the annual ILI season is important for increasing efficiency of healthcare systems and reducing costs to patients. Furthermore, the distinct, less predictable circulation of COVID-19 highlights the ongoing need for separate and robust surveillance strategies for SARS-CoV-2.

## Introduction

Influenza-like illnesses (ILI), defined by the US Centers for Disease Control (CDC) as illnesses causing a fever (usually 100 °F or 37.8 °C or higher) plus cough and/or sore throat,^1^ affect more than 9 to 41 million people in the US every year.^2^ ILI syndromic systems have operated for more than a decade in the United States (US). Indicators tracking ILI in the populations are frequently used as proxies of the timing of seasonal RSV and influenza outbreaks, which have historically started in autumn and ended by the beginning of spring. However, the COVID-19 pandemic brought about two major shifts to the respiratory disease landscape: first, it established the co-circulation of three pathogens with substantial epidemic potential which fall under the umbrella of ILI: influenza, respiratory syncytial virus (RSV), and SARS-CoV-2; second, it resulted in temporary enhancement of respiratory disease surveillance that enabled the disentanglement of these diseases during and between annual ILI seasons.^3^

While the symptom-based definition of ILI ensures that a majority of cases presenting to outpatient health facilities are detected by surveillance efforts, its lack of specificity results in the aggregation of cases of not only influenza A and B, RSV, and COVID-19, but also other respiratory infections before laboratory testing occurs.^4^ Each of the component infections of infections demonstrate different levels of morbidity and mortality, especially across age groups; for example, RSV infection is associated with higher rates of lower respiratory infection in young children^5^ while influenza, RSV, and COVID-19 are all associated with increased mortality among people aged 65 and over.^6^^,^^7^ Additionally, antiviral treatments for severe cases of these infections differ, with no overlap between US-approved treatments for the different viruses.^8^, ^9^, ^10^ Misidentification or misconceptions about the infections aggregated into ILI has substantial consequences for public health response and outbreak preparedness: although many ILI cases in the US are not life-threatening, surges in the volume of respiratory infections can stress health infrastructure and medical providers, especially as care regimens for different infections differ in cost and complexity.

Because respiratory illness surveillance, prevention, and interventions have historically focused on influenza in the US,^11^, ^12^, ^13^ healthcare systems and personnel are much more optimized to combat influenza outbreaks compared to outbreaks of other diseases. For example, while an influenza vaccination for a child age 6 months and older costs between US $15 and US $50 for a single dose, a comparable RSV vaccination costs between US $250 and US $500 per dose.^14^ Furthermore, the public health focus on influenza has also led to the persistence of misconceptions related to ILI and influenza among healthcare workers, such as believing peaks in ILI activity are caused only by outbreaks of influenza A followed by outbreaks of influenza B.^15^, ^16^, ^17^ Fortunately, new data streams introduced during the COVID-19 pandemic show promise in the ability to clearly distinguish between trends of influenza, RSV, and COVID-19 and to predict the onset and peaks of epidemics of these infections.^18^ These new data streams represent an advantageous resource in the post-pandemic era where influenza, RSV, and COVID-19 interact with each other and apply a combined influence on the healthcare system. Properly understanding the interactions of these diseases in addition to the changed healthcare landscape stemming from advancements in public health technology and adoption will be vital for future epidemiological and clinical response to outbreaks of respiratory disease.

Anticipating the timing of respiratory epidemics that drive the seasonal trajectory of ILI has several important implications for public health officials and medical practitioners. First, predicting the onset of an ILI season provides healthcare facilities and staff with advance notice to prepare for upcoming respiratory emergencies. Prediction algorithms and surveillance systems, such as those implemented by the CDC's FluSight initiative,^19^^,^^20^ aim to provide public health officials with valuable information to improve health communication and guide the allocation of resources, including both human personnel and protective equipment. Second, administration of therapeutics and vaccinations against respiratory infections is sensitive to timing, especially for at-risk populations such as young children or the elderly. For example, RSV prophylaxis for infants (a high-risk group for serious RSV infection) using the monoclonal antibody nirsevimab must be administered before the onset of an infant's first RSV season; infants born during the RSV season should receive nirsevimab within one week of birth.^21^ In addition, prior studies suggest passive immunity from nirsevimab wanes over time,^22^, ^23^, ^24^ with the waning period matching the average duration of a single RSV season. As a result, it would be ideal to administer nirsevimab doses no earlier than necessary for any specific region of the US. Clearer understanding and prediction of the timing of the start of the ILI season, especially as it relates to the onset of seasonal RSV surges, would allow healthcare facilities to increase stock of needed treatments and brief concerned parents well before the seasonal surge in RSV cases occurs. Improved understanding of seasonal RSV will complement established knowledge that immunity for influenza following vaccination undergoes significant waning after a few months.^25^^,^^26^ Therefore, timing for influenza vaccination and RSV experience similar public health constraints and distinguishing the two during an ILI season is critical to public health response. With these benefits in mind, this study aimed to improve our understanding of the observed timing and ordering of respiratory epidemics in the US that contribute to the trajectory of seasonal ILI.

## Methods

### Data sources

This study utilized publicly available, aggregated surveillance data from two complementary CDC systems: the National Syndromic Surveillance Program (NSSP)^27^ and the US Outpatient Influenza-like Illness Surveillance Network (ILINet). The NSSP provides near real-time electronic health data from a broad network of healthcare facilities across the United States.^27^ The program receives de-identified data from over 7200 facilities, encompassing approximately 83% of all emergency departments (EDs) in the 50 states and the District of Columbia.^28^ We use the weekly percentage of ED visits associated with chief complaints or diagnoses for influenza, COVID-19, or RSV as reported by NSSP as predictors in our modeling approach.

ILINet is a sentinel surveillance system that monitors outpatient ILI activity through a voluntary network of over 3400 healthcare providers across all 50 states, Puerto Rico, and the District of Columbia. Each week, sentinel providers report the total number of patient visits and the number of visits for ILI, defined as a fever with temperature of 100 °F [37.8 °C] or greater in combination with a cough and/or a sore throat. While the primary ILINet data is syndromic, a subset of these providers also participates in virologic surveillance by collecting respiratory specimens from patients with ILI for testing at public health laboratories. This optional testing helps identify circulating influenza strains but is not a required component for all ILINet reporting. The data used in our study reflects the aggregated weekly volume of outpatient ILI visits.

The final analysis incorporated 148 out of a possible 153 state-ILI seasons spanning the 2022–23, 2023–24, and 2024–25 ILI seasons. Exclusions were made due to data availability: three state-seasons from Missouri were excluded as the state does not report to NSSP, and the 2024-25 seasons for Wyoming and Vermont were excluded due to incomplete or unavailable NSSP time series data at the time of analysis. The analysis of the 2022–23 season begins on October 1, 2022, corresponding to the start of available NSSP data. All statistical analyses were performed using data up to March 30, 2025.

### Estimating the contribution of influenza, RSV and COVID-19 signals to ILI indicators

To estimate the relative contribution of influenza, RSV, and COVID-19 to the seasonal ILI signal, we developed and applied a series of independent regression models for each state-ILI season. An ILI season was defined as the period from May of one year to May of the following year to capture the full annual cycle of respiratory virus activity. For each of the 148 individual state-season datasets, we first normalized the time series of weekly percentage of visit data for ILI, influenza, RSV, and COVID-19 to a [0, 1] scale using min–max scaling for each time series, in each individual state, and during each season. This step ensures that the magnitudes of the different signals are comparable within the model. We then represented the normalized ILI signal, in each state and during each individual season (2022–23, 2023–24, 2024–25), as a linear combination of the three normalized pathogen signals using a ridge regression (L2 penalty) framework. Each model's coefficients (β) were constrained to be positive to ensure that each pathogen could only have a non-negative contribution to the ILI signal. The model intercept was removed to attribute the ILI signal exclusively to influenza, RSV, and COVID-19. We also conducted a sensitivity analysis where we included a non-zero intercept and found very similar results (Supplementary Figure S1). The regularization parameter λ was optimized for each model individually via a cross-validation-informed grid search. A full mathematical description of each model is provided in the Supplementary Methods. The resulting coefficients represent the relative contribution of each pathogen. By multiplying the normalized pathogen time series by their respective coefficients, we generated a set of contribution-weighted time series, which were used to identify the timing of peak viral activity. The ridge regressions were implemented in R version 4.5.1 using the *glmnet* package.

### Evaluating the time difference between epidemics

We also carried out analyses to determine the time difference between the peaks and onsets of RSV and influenza seasonal epidemic outbreaks. To perform these analyses, we used an anomaly detection methodology previously used by Kogan et al.^29^ and Stolerman et al.^30^ to determine the timing of the onsets of each epidemic time series. This method serves to provide a more robust mathematical structure behind the results we observed in the regression-based approach detailed above. Specifically, for each week *t* of our time series, we assessed the preceding 6 weeks and determined if exponential growth of cases was observed. This was done by computing a retrospective linear regression model on the time series at week *t* to find the multiplicative constant that maps the number of cases at time *t* to those occurring at time *t* + *1* (i.e., the coefficient *λ*_*t*_ of a lag-1 autoregressive model without an intercept) using data from the immediate six-week time period preceding week *t. λ*_*t*_ is a proxy of the effective reproductive number R_t_ which is a commonly used epidemiological measure to determine whether an epidemic is growing or decreasing in size.^29^^,^^30^ Outbreaks were identified as the first point in time when *λ*_*t*_ was larger than 1 for a sustained period of six weeks. We choose this six-week evaluation period to reduce the chance of mislabeling noisy oscillations in the data as outbreaks; sensitivity analyses of this 6-week evaluation period (Supplementary Figures S2–S27) found that changes to this window did not result in substantial differences in the results.

To determine epidemic peaks, we used the *find_peaks* function from the Python library SciPy to identify peak values of our disease time series. After identification, each peak was assessed and selected based on the following three criteria: 1) peaks had to span a minimum duration of 2.5 weeks; 2) the peak value had to exceed the 75th percentile of the historical time series for each state; and 3) each peak needed to be more than 20 weeks apart from any previously detected peak unless it was higher than a previously detected peak that had occurred within the last 20 weeks. We chose the peak width of 2.5 weeks to obtain epidemiologically relevant peaks; we used such a width parameter because the anomaly detection algorithm generally functions in real time and must account for the possibility of future peaks in its detection decisions. We provide a pseudocode example for the onset detection algorithm in the Supplementary Methods, while a detailed discussion of these parameters and a methodological justification for the parameter values we used in this study can be found elsewhere.^31^ The anomaly detection algorithms described above were implemented in Python version 3.10 using the *SciPy* package.

Given the criteria for peak and onset detection and the unavailability of data from before October 2022, we only used the anomaly detection method to analyze the 23–24 and 24–25 seasons. For both methods, we calculated the median and 95% percentile range (PR) for the median time difference between two peaks or between two onsets for all assessed state-seasons. We obtained the PRs for the medians using a non-parametric bootstrap method. Specifically, we generated 1000 bootstrap resamples by sampling the observed set of state-season peak time differences with replacement. We calculated the median for each resample, and the 95% PR represents the 2.5th and 97.5th percentiles of the resulting distribution of 1000 medians.

This study utilized aggregated, de-identified surveillance data that are publicly available. The study was determined to not constitute human subjects research, and institutional review board (IRB) approval was not required.

### Role of the funding source

The funders of the study had no role in the study design, data collection, data analysis, data interpretation, or writing of the report. All authors had full access to all the data in the study and agreed to submit the report for publication.

## Results

**Fig. 1:**
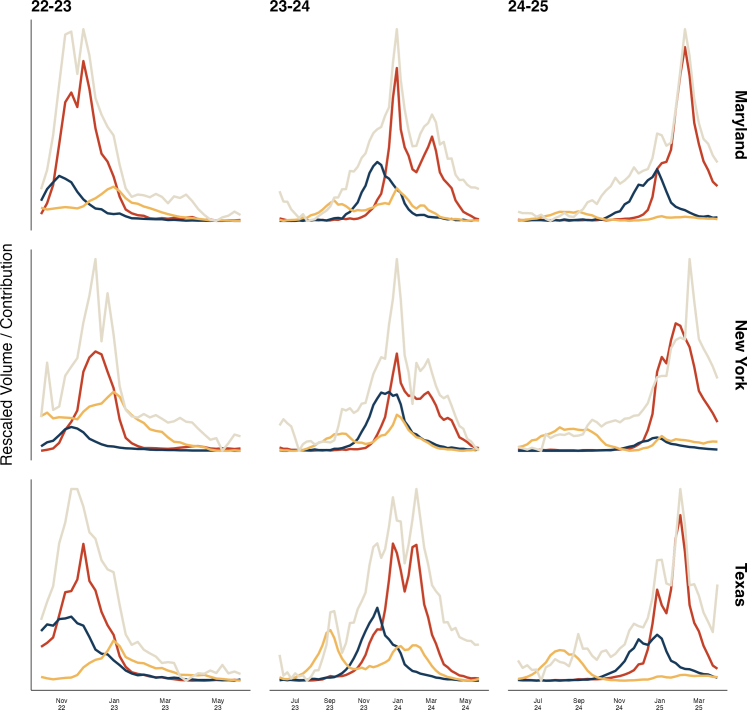
**RSV epidemics precede influenza epidemics during many ILI seasons, while COVID-19 co-circulates.** In each of the three states displayed (Maryland, New York, and Texas), RSV volume (blue) reaches its peak value before influenza volume (red) in each of the three analyzed seasons. COVID-19 volume (gold) peaked after both RSV and influenza in 22–23 but peaked before RSV and influenza in 23–24 and 24–25. Curves for RSV, flu, and COVID-19 represent contribution-weighted time series, generated by multiplying normalized pathogen data by coefficients from our regression model. The ILI curve (grey) is the min–max scaled time series. Abbreviations: COVID-19: coronavirus disease 2019, flu: influenza, ILI: influenza-like illness, RSV: respiratory syncytial virus.

We analyzed 148 state-ILI seasons across the 2022–23, 2023–24, and 2024–25 ILI seasons (henceforth the 22–23, 23–24, and 24–25 seasons), encompassing data from 49 US states and the District of Columbia (Fig. 1). From this data, we identified three main patterns of epidemic timings. In the most common and most relevant pattern, RSV epidemics reached peak volume before influenza epidemics (114/148 state-seasons, 77.0%). Secondary to this pattern were seasons in which RSV epidemics and influenza epidemics peaked concurrently (i.e., the peak volume of ED visits for both RSV and influenza occurred within the same 7-day period) (14/148 seasons, 9.5%) and seasons in which influenza epidemics peaked before RSV epidemics (20/148 seasons, 13.5%) (Supplementary Figures S28–S30).

ILI seasons most frequently reached their peak volume in November and December, with some late surges in February and March in the 23–24 and 24–25 seasons (Supplementary Figure S31). Peak volume of RSV in the 22–23 season occurred as early as the first week of November (in Alabama, Mississippi, and Tennessee) and as late as the last week of December (in Montana). In the 23–24 season, the earliest RSV peak also occurred in the first week of November (in Florida); however, the latest peak occurred much later in the year, spiking in late February (in South Dakota). In the 24–25 season, the earliest RSV peak occurred in late November (in Florida, Georgia, and Mississippi) and the latest RSV peak occurred in early March (in Montana and South Dakota). Late peaks of ILI were observed in December (in the 22–23 season) and as late as March (in the 23–24 season). Across the three analyzed ILI seasons, COVID-19 did not demonstrate strictly annual seasonality like either RSV or influenza. While COVID-19 volume peaked between November and January in the 22–23 season, we observed multiple epidemics of COVID-19 in the 23–24 season in several states and only an early epidemic of COVID-19 in the 24–25 season (during the summer months of July–September). We observed these patterns in both the regression based analyses and the additional correlation-based analyses (Supplementary Figures S32–S37).

**Fig. 2:**
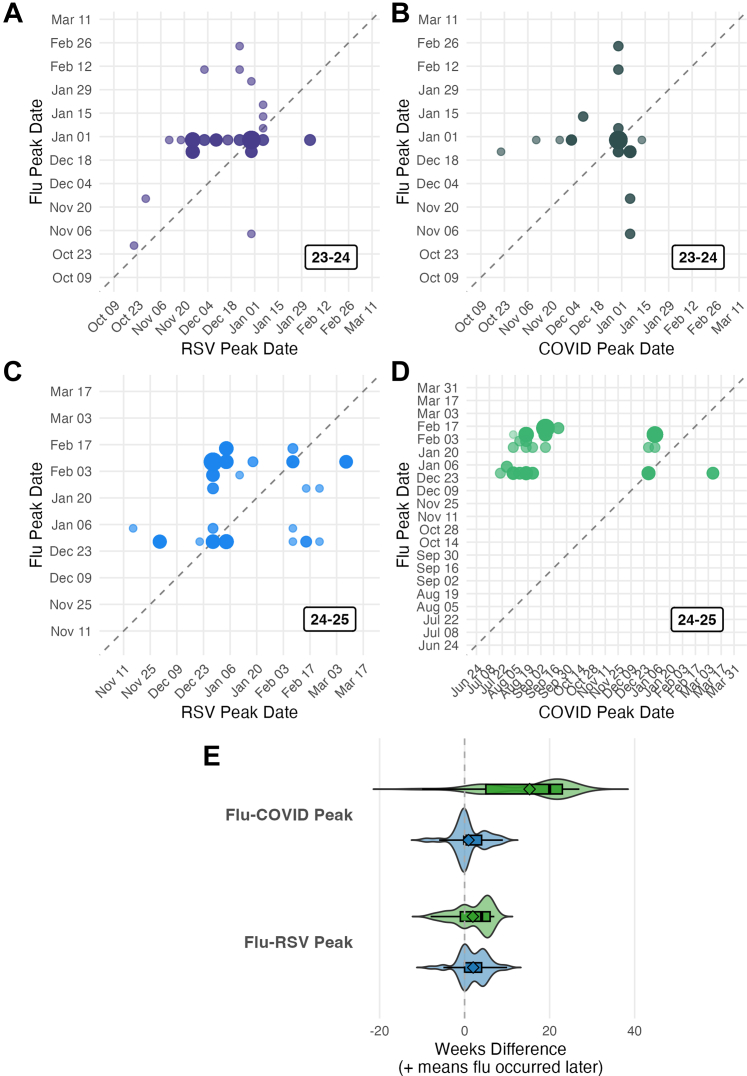
**Anomaly detection methods indicate RSV peaks before influenza in most seasons, while COVID-19 exhibits irregular seasonality.** Points above the diagonal line indicate that the disease on the *y*-axis (influenza) peaks after the disease on the *x*-axis (either RSV or COVID-19). Larger points indicate that peaks in multiple states occurred during the same week. **A, B:** RSV peaked before influenza, while the COVID-19/influenza relationship is less clear in the 22–23 season. **C, D:** While the RSV-influenza relationship remains consistent, COVID-19 seasonality is noticeably different in the 24–25 season. **E:** Distribution and spread of the difference (in weeks) of RSV, COVID-19 and influenza peaks. Blue violins represent data from 23 to 24 and green violins represent data from 24 to 25. Abbreviations: COVID-19: coronavirus disease 2019, flu: influenza, RSV: respiratory syncytial virus.

We then used our anomaly detection method to quantify the time differences between onsets and peaks of epidemics (Fig. 2). First, we identified that the median difference between peaks of influenza and RSV epidemics was +2.0 weeks (95% percentile range [PR]: −5.0, +9.0) in the 23–24 season and +4.0 weeks (95% PR: −7.0, +6.2) in the 24–25 season (the + sign indicates that the RSV peak occurred before the influenza peak). Next, we also determined that the median time between the onsets of influenza and RSV epidemics was +3.0 weeks (95% PR: −7.4, +11.7) in the 23–24 season and +0.05 weeks in the 24–25 season (95% PR: −11.0, +11.0) (Supplementary Figure S38). The results of our analyses comparing peaks and onsets of COVID-19 to peaks and onsets of influenza were inconclusive as the onset and peak pattern of COVID-19 in the 23–24 season differed substantially from the onset and peak pattern of COVID-19 in the 24–25 season. We observed two epidemics of COVID-19 in most states in the summer of 2023 and in the winter between 2023 and 2024 but only observed a summer surge in COVID-19 volume in the 2024–25 season. Because our comparison assumes an overall seasonal trajectory like that of ILI (i.e., regular, seasonal outbreaks occurring within a similar timeframe), the inconsistent seasonality of COVID-19 represents a challenge for future research.

## Discussion

In this study, we used data from the past three ILI seasons in the United States to clarify the temporal sequencing of epidemics of influenza and RSV. We deployed regularized regression models to evaluate the relative order of influenza, RSV and COVID-19 epidemics and their contribution to the overall trajectory of the ILI season. We determined that in 114 out of 148 recent state-ILI seasons (77.0%), RSV epidemics peaked before influenza epidemics. We then used an anomaly detection method to quantify the time differences between these epidemics and determined that there is a three-week median separation (range: −8, +10 weeks) between peaks of RSV epidemics and peaks of influenza epidemics. Taken together, these results suggest that public health authorities and healthcare providers should be ready to prepare for surges in RSV volume likely before surges in influenza volume (if this pattern persists), especially before and during the start of the traditional ILI season in early autumn.

The finding that the peaks of RSV epidemics preceded influenza epidemics in many of the recent ILI seasons in the United States has notable implications for public health authorities and healthcare providers. Based on our analyses, increases in the volume of RSV hospitalizations are a likely indicator for the start of the yearly ILI season. We also found that the ILI seasons we analyzed were consistently initiated by epidemics of RSV in the Southeastern United States; this geographic pattern aligns with the findings of previous work assessing trends in RSV^32^ in addition to work evaluating the spatiotemporal trends of other respiratory infections such as streptococcal pharyngitis.^33^ The sources of this consistent outbreak initiation in usually warm Southeastern states during the start of winter should be investigated in future studies, especially as they potentially relate to changes in climate and in the environment. These findings also suggest that detection of surges of RSV cases in one state jurisdiction in late autumn or early winter should trigger alarms to health officials in nearby states for the onset of the annual ILI season, even before outpatient cases of RSV or influenza are reported. Furthermore, compared to the influenza and RSV epidemics, we observed irregular seasonality of COVID-19 outbreaks in this post-pandemic period which did not present in any easily recognizable pattern. Recent work has suggested that COVID-19 demonstrates bi-annual seasonality,^34^^,^^35^ but continued data collection and analysis that tracks the circulation of SARS-CoV-2 is warranted.

Our findings also have direct implications for the administration of RSV prophylaxis with the monoclonal antibody nirsevimab. Current guidance from the Advisory Committee on Immunization Practices (ACIP) recommends seasonal administration from October through March for most of the continental United States.^36^ The large number of children who primarily receive vaccines through the CDC's Vaccines for Children (VFC) program means that state VFC programs generally cannot deviate from this window. However, our analyses reveal substantial state-level variability in the timing of RSV epidemic onsets and peaks. The broad, fixed window for nirsevimab administration is a practical compromise for national-level guidance, balancing the need to protect infants before the season starts against the realities of waning passive immunity and the significant cost of the therapeutic. A critical future direction is to use these data to quantify the portion of RSV cases that occur outside the standard ACIP window and to determine if these deviations follow predictable patterns. For instance, whether states in certain regions consistently experience later seasons would significantly impact nirsevimab administration policy through state VFC programs. Ultimately, our work suggests that a data-informed methodology to recommend administration windows on a state-by-state basis would be possible, allowing public health authorities to leverage real-time surveillance to issue more precise guidance and better align prophylaxis with the true local onset of the RSV season.

Additionally, while we focused on temporal relationships in this study, we recognize that the ordering, peaks, and onsets of respiratory epidemics may also be influenced by spatial factors. We observed a pattern of early-season epidemics occurring in the southeastern US that are followed by spreading across the country in a north-western direction for both influenza and RSV which resembled spatiotemporal patterns observed for other respiratory diseases as mentioned previously. We visualized this spread at the weekly level (Supplementary Figures S39 and S40) for both the 23–24 and 24–25 seasons and observed that the timings of RSV and influenza onsets both demonstrated the southeast to northwest spread pattern. On the other hand, peak timings for both influenza and RSV were more varied, with different sequences of peak timings occurring across the two analyzed seasons. While our interpretation of the spreading pattern across the country is limited by our weekly time resolution, we were still able to observe a gradual progression of epidemic onsets in a northwesterly direction. Additionally, these temporal patterns suggest that influenza and RSV patterns may be more regional rather than localized at the state level; as Supplementary Figures S39 and S40 suggest, similarities in climatic and weather patterns of states in the Southeast, Midwest, and Northwest point future research toward spatial models which take this regional clustering into account and analyze region-seasons of ILI (though this would result in a smaller orverall sample size). Future research should take advantage of finer-resolution data, such as data collected at the daily level, to track the movement of epidemics during ILI seasons.

While our results provide a top-down overview of the timing and ordering of peaks of RSV and influenza epidemics, questions about the reasons for the temporal separation between RSV and influenza remain. For example, while our findings concur with those of past studies evaluating the timing of RSV and influenza epidemics in Argentina,^37^ Spain,^38^ the Netherlands,^39^ and other work done at the global scale^40^ identified that RSV epidemics in temperate regions (like the US) occurred before influenza epidemics, but that this relationship was not present in tropical regions. While this conclusion implicates weather and temperature changes as drivers of the relationship between RSV and influenza, it does not fully explain why RSV peaks before influenza in the US. One possible rationale for the earlier rise of RSV epidemics is that they are driven by the congregation of children of school age; however, since the average school opening occurs in late August to early September in the US,^41^ it is unlikely that this congregation is the sole driver of RSV epidemics. It is therefore possible for a combination of climate and increased contact among younger individuals which promotes the growth of either RSV or influenza epidemics. An alternative possibility is that both RSV and influenza are re-introduced to seasonal circulation at similar times, but RSV peaks earlier because of its increased transmissibility compared to seasonal influenza.^42^^,^^43^ Future work should carefully inspect drivers of respiratory epidemics through modeling or surveillance studies and identify the potential differences among those drivers.

Finally, this study has several limitations. First, our analyses relied on data reported to the CDC's National Syndromic Surveillance System by participating emergency departments in hospitals nationwide and therefore do not necessarily capture the entire burden of ILI, influenza, or RSV cases. Testing protocols (especially those related to COVID-19) varied over time, which may impact the quality of the source data; however, the NSSP is a major source for respiratory disease surveillance data which covers a majority of healthcare institutions in the US. Second, our data were aggregated at the weekly level and therefore we could only temporally order individual epidemics within periods of 7 days; while this data structure can exhibit autocorrelation, especially when considering the regression models' error terms, we avoid issues of cross-season autocorrelation by fitting independent models to each individual state-season. Third, we only had data for one fully complete ILI season and two mostly complete seasons due to the nature of the dataset used; future surveillance and analysis work should continue using this data moving forward to emphasize the robustness of our results. Fourth, the anomaly detection approach we employed to detect peaks and onsets assumes uncertainty for these timepoints can be ignored; such uncertainty may be more pronounced in assessing a long-term sequence of epidemic onsets. Finally, our analysis treats each state as an independent unit; however, we recognize that epidemic timing is spatially correlated, which may reduce the effective sample size of our observations. Future work should incorporate formal spatial statistical models to account for these dependencies.

## Contributors

Dewey: Conceptualization, Formal Analysis, Investigation, Methodology, Software, Visualization, Writing–Original Draft, Writing–Review and Editing.

Meyer: Conceptualization, Formal Analysis, Investigation, Methodology, Writing–Review and Editing.

Garcia: Formal Analysis, Methodology, Software, Writing–Review and Editing.

Santillana: Conceptualization, Formal Analysis, Investigation, Methodology, Supervision, Writing–Original Draft, Writing–Review and Editing.

All authors reviewed and edited the manuscript. GD made the decision to submit the manuscript for publication.

## Data sharing statement

Data and code used in this article can be found at github.com/MIGHTE-lab/rsv_peak_timing. The original data is provided by the United States Centers for Disease Control and Prevention through data.cdc.gov.

## Declaration of interests

AM received consulting fees from Northeastern University for research work with the Machine Intelligence Group for the Betterment of Health and the Environment (MIGHTE). MS received institutional research funds from the Johnson & Johnson Foundation and from Janssen Global Public Health. GD and RG report no conflicts of interest.

## References

[1] World Health Organization (2025). WHO surveillance case definitions for ILI and SARI. https://www.who.int/teams/global-influenza-programme/surveillance-and-monitoring/case-definitions-for-ili-and-sari.

[2] Centers for Disease Control and Prevention (2025). Influenza - about estimated flu burden. https://www.cdc.gov/flu-burden/php/about/index.html.

[3] Clark E.C., Neumann S., Hopkins S., Kostopoulos A., Hagerman L., Dobbins M. (2024). Changes to public health surveillance methods due to the COVID-19 pandemic: scoping review. JMIR Public Health Surveill.

[4] Spencer J.A., Shutt D.P., Moser S.K. (2022). Distinguishing viruses responsible for influenza-like illness. J Theor Biol.

[5] Kwon J.H., Paek S.H., Park S.H., Kim M.J., Byun Y.H., Song H.Y. (2024). COVID-19, influenza, and RSV in children and adults: a clinical comparative study of 12,000 cases. J Clin Med.

[6] Bajema K.L., Bui D.P., Yan L. (2025). Severity and long-term mortality of COVID-19, influenza, and respiratory syncytial virus. JAMA Intern Med.

[7] Hanage W.P., Schaffner W. (2025). Burden of acute respiratory infections caused by influenza virus, respiratory syncytial virus, and SARS-CoV-2 with consideration of older adults: a narrative review. Infect Dis Ther.

[8] Tejada S., Martinez-Reviejo R., Karakoc H.N., Peña-López Y., Manuel O., Rello J. (2022). Ribavirin for treatment of subjects with respiratory syncytial virus-related infection: a systematic review and meta-analysis. Adv Ther.

[9] Chan-Tack K.M., Murray J.S., Birnkrant D.B. (2009). Use of ribavirin to treat influenza. N Engl J Med.

[10] Bonneux B., Jacoby E., Ceconi M., Stobbelaar K., Delputte P., Herschke F. (2024). Direct-acting antivirals for RSV treatment, a review. Antiviral Res.

[11] Cowling B.J., Wong I.O., Ho L.-M., Riley S., Leung G.M. (2006). Methods for monitoring influenza surveillance data. Int J Epidemiol.

[12] Leuba S.I., Yaesoubi R., Antillon M., Cohen T., Zimmer C. (2020). Tracking and predicting US influenza activity with a real-time surveillance network. PLoS Comput Biol.

[13] Reed C., Chaves S.S., Daily Kirley P. (2015). Estimating influenza disease burden from population-based surveillance data in the United States. PLoS One.

[14] Centers for Disease Control and Prevention (2025). Current CDC vaccine price list. https://www.cdc.gov/vaccines-for-children/php/awardees/current-cdc-vaccine-price-list.html.

[15] Caini S., Andrade W., Badur S. (2016). Temporal patterns of influenza A and B in tropical and temperate countries: what are the lessons for influenza vaccination?. PLoS One.

[16] Dhanasekaran V., Sullivan S., Edwards K.M. (2022). Human seasonal influenza under COVID-19 and the potential consequences of influenza lineage elimination. Nat Comm.

[17] Borchering R.K., Gunning C.E., Gokhale D.V. (2021). Anomalous influenza seasonality in the United States and the emergence of novel influenza B viruses. Proc Natl Acad Sci U S A.

[18] Centers for Disease Control and Prevention (2024). NSSP support during and after the COVID-19 pandemic. https://www.cdc.gov/nssp/php/partnerships/nssp-support-during-after-covid-19-pandemic.html.

[19] McGowan C.J., Biggerstaff M., Johansson M. (2019). Collaborative efforts to forecast seasonal influenza in the United States, 2015–2016. Sci Rep.

[20] Mathis S.M., Webber A.E., León T.M. (2024). Evaluation of FluSight influenza forecasting in the 2021–22 and 2022–23 seasons with a new target laboratory-confirmed influenza hospitalizations. Nat Comm.

[21] American Academy of Pediatrics (2024). Nirsevimab administration. https://www.aap.org/en/patient-care/respiratory-syncytial-virus-rsv-prevention/nirsevimab-administration/.

[22] Wilkins D., Yuan Y., Chang Y. (2023). Durability of neutralizing RSV antibodies following nirsevimab administration and elicitation of the natural immune response to RSV infection in infants. Nat Med.

[23] Moline H.L., Tannis A., Toepfer A.P. (2024). Early estimate of nirsevimab effectiveness for prevention of respiratory syncytial virus-associated hospitalization among infants entering their first respiratory syncytial virus season - new vaccine surveillance network, October 2023-February 2024. MMWR Morb Mortal Wkly Rep.

[24] Xu H., Aparicio C., Wats A. (2025). Estimated effectiveness of nirsevimab against respiratory syncytial virus. JAMA Netw Open.

[25] Grohskopf L.A., Ferdinands J.M., Blanton L.H., Broder K.R., Loehr J. (2024). Prevention and control of seasonal influenza with vaccines: recommendations of the advisory committee on immunization practices - United States, 2024-25 influenza season. MMWR Recomm Rep.

[26] Tokars J.I., Patel M.M., Foppa I.M., Reed C., Fry A.M., Ferdinands J.M. (2020). Waning of measured influenza vaccine effectiveness over time: the potential contribution of leaky vaccine effect. Clin Infect Dis.

[27] Centers for Disease Control and Prevention (2025). National syndromic surveillance system (NSSP). https://www.cdc.gov/nssp/index.html.

[28] Centers for Disease Control and Prevention (2025). NSSP and health care providers. https://www.cdc.gov/nssp/hcp/about/index.html.

[29] Kogan N.E., Clemente L., Liautaud P. (2021). An early warning approach to monitor COVID-19 activity with multiple digital traces in near real time. Sci Adv.

[30] Stolerman L.M., Clemente L., Poirier C. (2023). Using digital traces to build prospective and real-time county-level early warning systems to anticipate COVID-19 outbreaks in the United States. Sci Adv.

[31] Garrido Garcia R., Clemente L., Meyer A., Dewey G., Yang S., Santillana M. (2025). A prospective real-time early warning system to anticipate onsets and peaks of respiratory diseases outbreaks at the state level in the U.S.A transfer learning approach leveraging digital traces. medRxiv.

[32] Pitzer V.E., Viboud C., Alonso W.J. (2015). Environmental drivers of the spatiotemporal dynamics of respiratory syncytial virus in the United States. PLoS Pathog.

[33] Kline M.C., Kissler S.M., Whittles L.K., Barnett M.L., Grad Y.H. (2024). Spatiotemporal trends in group A streptococcal pharyngitis in the United States. Clin Infect Dis.

[34] Rubin I.N., Bushman M., Lipsitch M., Hanage W.P. (2025). Seasonal forcing and waning immunity drive the sub-annual periodicity of the COVID-19 epidemic. medRxiv.

[35] Rose E.B., Paden C., Cook P. (2025). Estimated COVID-19 periodicity and correlation with SARS-CoV-2 spike protein S1 antigenic diversity, United States. Emerg Infect Dis.

[36] Jones J.M., Fleming-Dutra K.E., Prill M.M. (2023). Use of nirsevimab for the prevention of respiratory syncytial virus disease among infants and young children: recommendations of the advisory committee on immunization practices - United States, 2023. MMWR Morb Mortal Wkly Rep.

[37] Baumeister E., Duque J., Varela T. (2019). Timing of respiratory syncytial virus and influenza epidemic activity in five regions of Argentina, 2007-2016. Influenza Other Respir Viruses.

[38] Míguez A., Iftimi A., Montes F. (2016). Temporal association between the influenza virus and respiratory syncytial virus (RSV): RSV as a predictor of seasonal influenza. Epidemiol Infect.

[39] van Asten L., Bijkerk P., Fanoy E. (2016). Early occurrence of influenza a epidemics coincided with changes in occurrence of other respiratory virus infections. Influenza Other Respir Viruses.

[40] Li Y., Reeves R.M., Wang X. (2019). Global patterns in monthly activity of influenza virus, respiratory syncytial virus, parainfluenza virus, and metapneumovirus: a systematic analysis. Lancet Glob Health.

[41] Desilver D. (2023). ‘Back to school’ means anytime from late july to after labor day, depending on where in the U.S. you live. https://www.pewresearch.org/short-reads/2023/08/25/back-to-school-dates-u-s/.

[42] Reis J., Shaman J. (2016). Retrospective parameter estimation and forecast of respiratory syncytial virus in the United States. PLoS Comput Biol.

[43] Biggerstaff M., Cauchemez S., Reed C., Gambhir M., Finelli L. (2014). Estimates of the reproduction number for seasonal, pandemic, and zoonotic influenza: a systematic review of the literature. BMC Infect Dis.

