# Supplementary material for: Uncovering the post-pandemic timing of influenza, RSV, and COVID-19 driving seasonal influenza-like illness in the United States: a retrospective ecological study
Source: Lancet Reg Health Am. 2026 Jan 1;55:101359. doi: 10.1016/j.lana.2025.101359 (PMC12804126; doi:10.1016/j.lana.2025.101359)
Supplement: Supplementary Material [file mmc1.docx]

**Supplementary Information for Uncovering the Post-Pandemic Timing of Influenza, RSV, and COVID-19 Driving Seasonal Influenza-like Illness in the United States: A Retrospective Ecological Study**

George Dewey, Austin G. Meyer, Raul Garrido Garcia, Mauricio Santillana

Table of Contents

[Supplementary Methods 2](#_Toc216701041)

[Specification of Ridge Regression Model for ILI Decomposition 2](#_Toc216701042)

[Specification of Retrospective Model for Onset Detection 3](#_Toc216701043)

[Pseudocode for Onset Anomaly Detection Method. 4](#_Toc216701044)

[Supplementary Figures 5](#_Toc216701045)

[Supplementary Figure 1: Beta coefficient comparison of regression models with and without intercept. 5](#_Toc216701046)

[Supplementary Figures 2-27: Sensitivity Analyses for Onset Detection Threshold 6](#_Toc216701047)

[Supplementary Figure 28: Timing of Respiratory Epidemics in the United States for the 2022-2023 ILI season. 32](#_Toc216701048)

[Supplementary Figure 29: Timing of Respiratory Epidemics in the United States for the 2023-2024 ILI season. 33](#_Toc216701049)

[Supplementary Figure 30: Timing of Respiratory Epidemics in the United States for the 2024-2025 ILI season. 34](#_Toc216701050)

[Supplementary Figure 31: State-level timelines of epidemic peaks based on the regression method for the 22-23, 23-24, and 24-25 ILI seasons in the United States. 35](#_Toc216701051)

[Supplementary Figures 32-37: Lagged Correlation Analysis 36](#_Toc216701052)

[Supplementary Figure 38: Anomaly detection methods show no clear precedence of RSV or COVID-19 before influenza when comparing epidemic onsets. 43](#_Toc216701053)

[Supplementary Figure 39: Anomaly detection methods indicate influenza and RSV epidemic onsets spread in a southeast to northwest direction the 2023-24 and 2024-25 ILI seasons 44](#_Toc216701054)

[Supplementary Figure 40: Anomaly detection methods indicate influenza and RSV epidemic peak spread in a southeast to northwest direction the 2023-24 and 2024-25 ILI seasons. 45](#_Toc216701055)

## Supplementary Methods

### Specification of Ridge Regression Model for ILI Decomposition

We fit an independent ridge regression model for each state *s* and ILI season *y*. The objective function of the regression model is to find the coefficients (*β*) that minimize:

$$\min_{\beta_{p, s, y}} ( \sum_{t=1}^{T_{s,y}} \left\| ILI_{s,y}\left( t \right)-\sum_{p\in P} \beta_{p,s,y}\cdot Pathogen_{p,s,y}\left( t \right) \right\|_{2}^{2}+\lambda\sum_{\left\| \beta_{p,s,y} \right\|_{2}^{2}} \left\| \beta_{p,s,y} \right\|_{2}^{2} )$$

where $\beta_{p,s,y}\geq0$ for all *p*.

The parameters are defined as follows:

- *t* is the week in season T_s,y_ (2022, 2023, or 2024)
- *p* is the specific pathogen: influenza, RSV, COVID-19
- ILI_s,y_(t) is the normalized weekly percentage of ED visits for ILI for state *s* and season *y* in week *t*
- $\beta_{p,s,y}$ is the non-negative regression coefficient for pathogen *p*, for state *s* and season (year) *y*.
- $\lambda$ is the regularization parameter which penalizes the sum of the squares of the coefficients.

### Specification of Retrospective Model for Onset Detection

To identify epidemic onsets, we computed a proxy for the effective reproductive number $\lambda_{t}$for each week *t*. This was done by fitting a lag-1 autoregressive model to the preceding six weeks of epidemic volume. The model is specified as:

$$Volume \left( t^{'} \right)=\lambda_{t}\cdot Volume\left( t^{'}-1 \right)+\epsilon$$

where *t’* includes the six weeks of data immediately preceding week *t.* The effective reproductive number $\lambda_{t}$is estimated using ordinary least squares linear regression. An onset was identified as the first point where $\lambda_{t}$>1 for a period of 6 weeks.

| 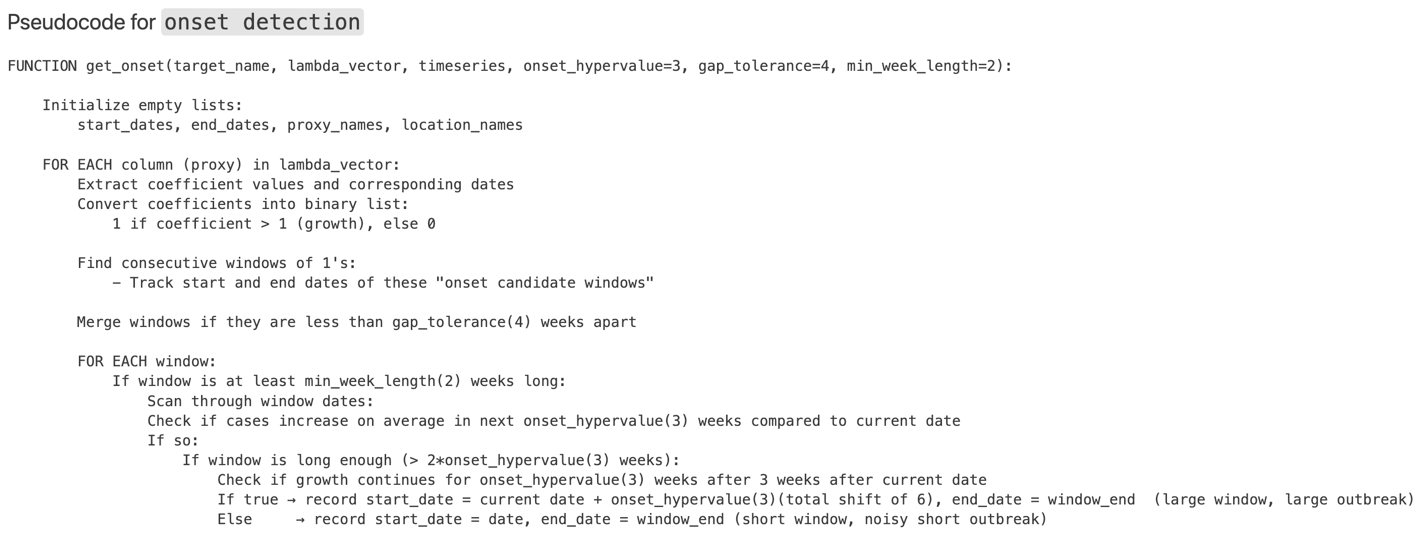 |
| --- |
| Pseudocode for Onset Anomaly Detection Method. |

## Supplementary Figures

| **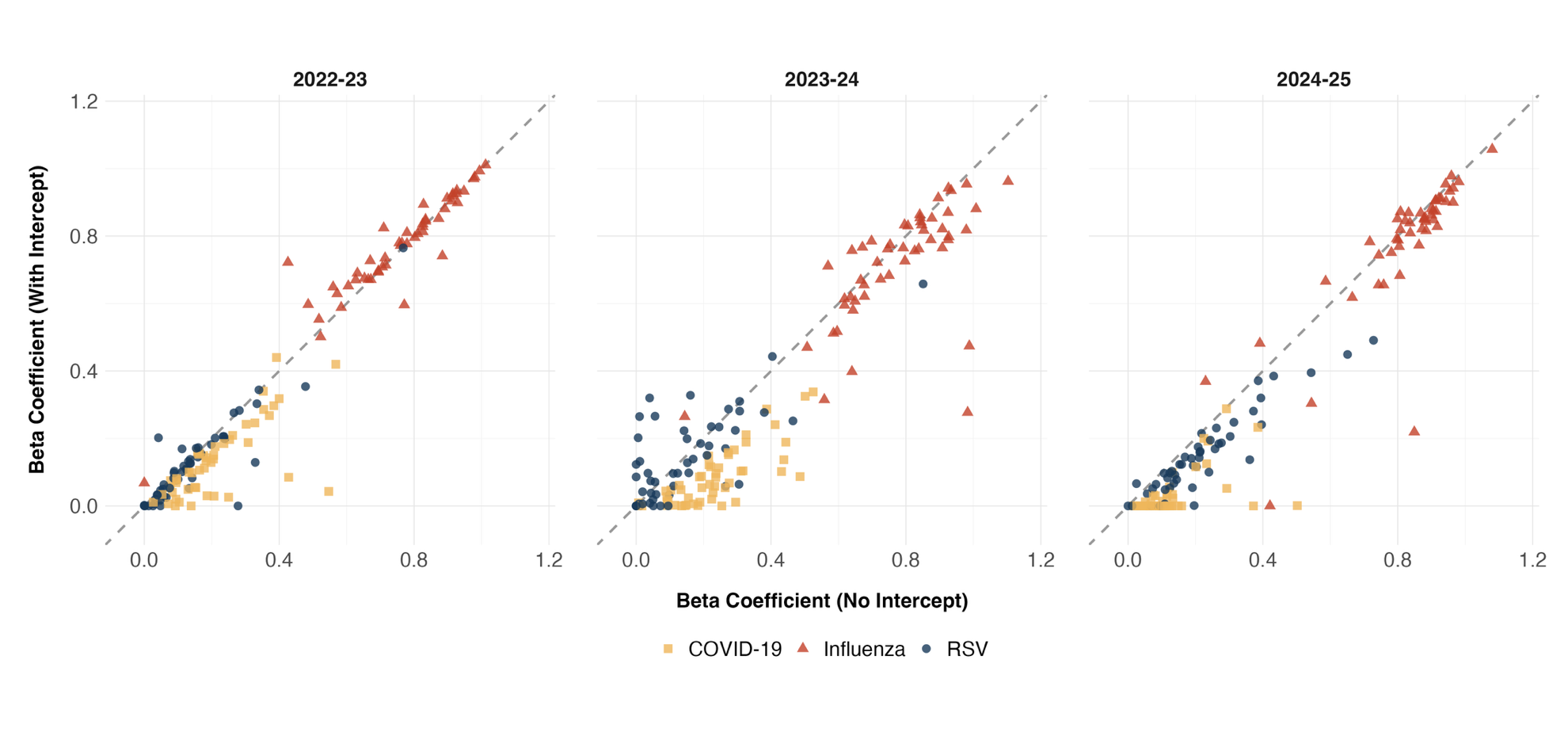** |
| --- |
| Supplementary Figure 1: Beta coefficient comparison of regression models with and without intercept. There was no substantial difference between the results of the without-intercept and with-intercept models. |

### Supplementary Figures 2-27: Sensitivity Analyses for Onset Detection Threshold

| 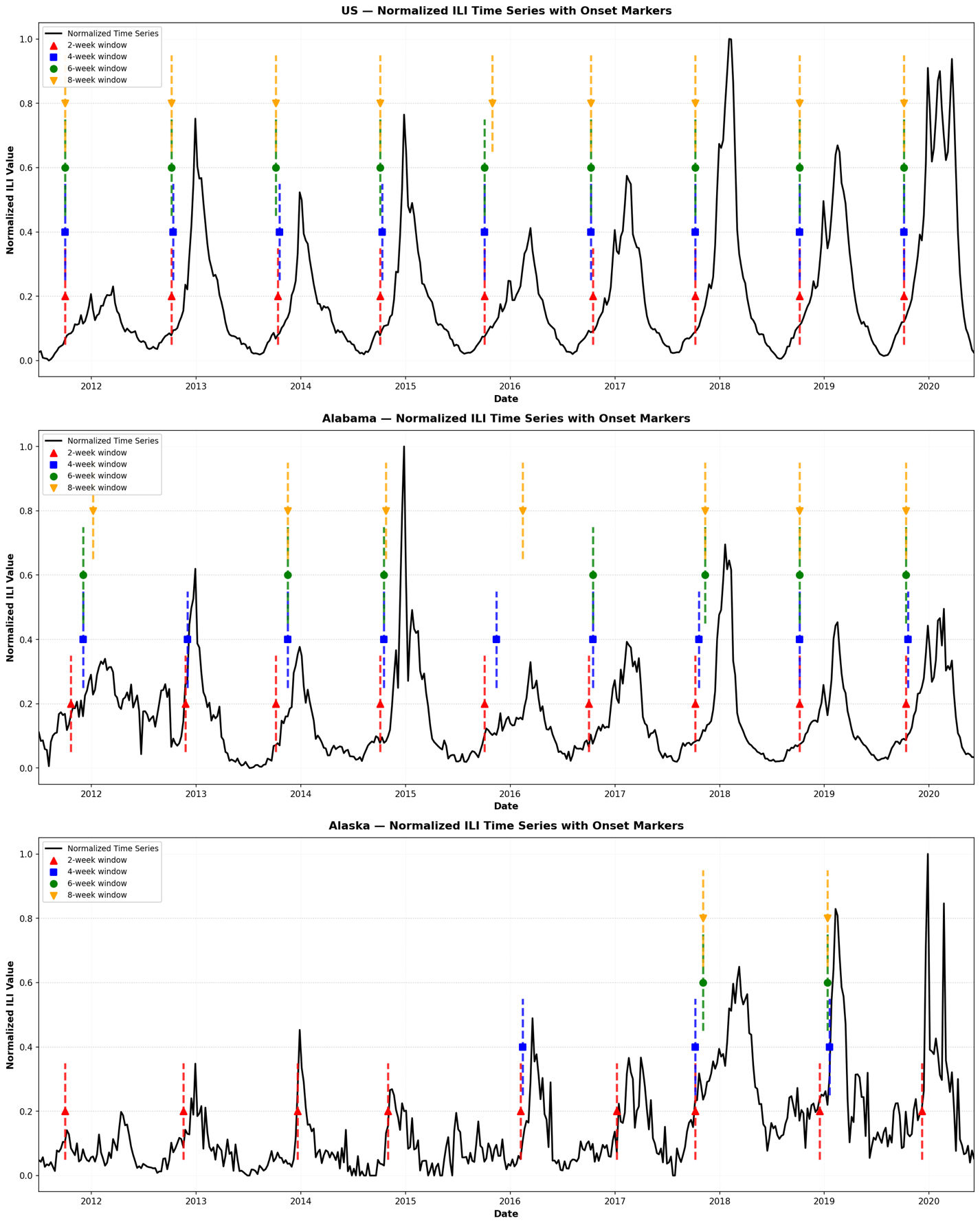 |
| --- |
| **Supplementary Figure 2**: **Sensitivity Analysis Results for Onset Detection Threshold for ILI (US, Alabama, Alaska)**. Reducing the window size below 6 weeks results in increases in false positive rate, while increasing the window size to 8 weeks misses outbreaks. |

| 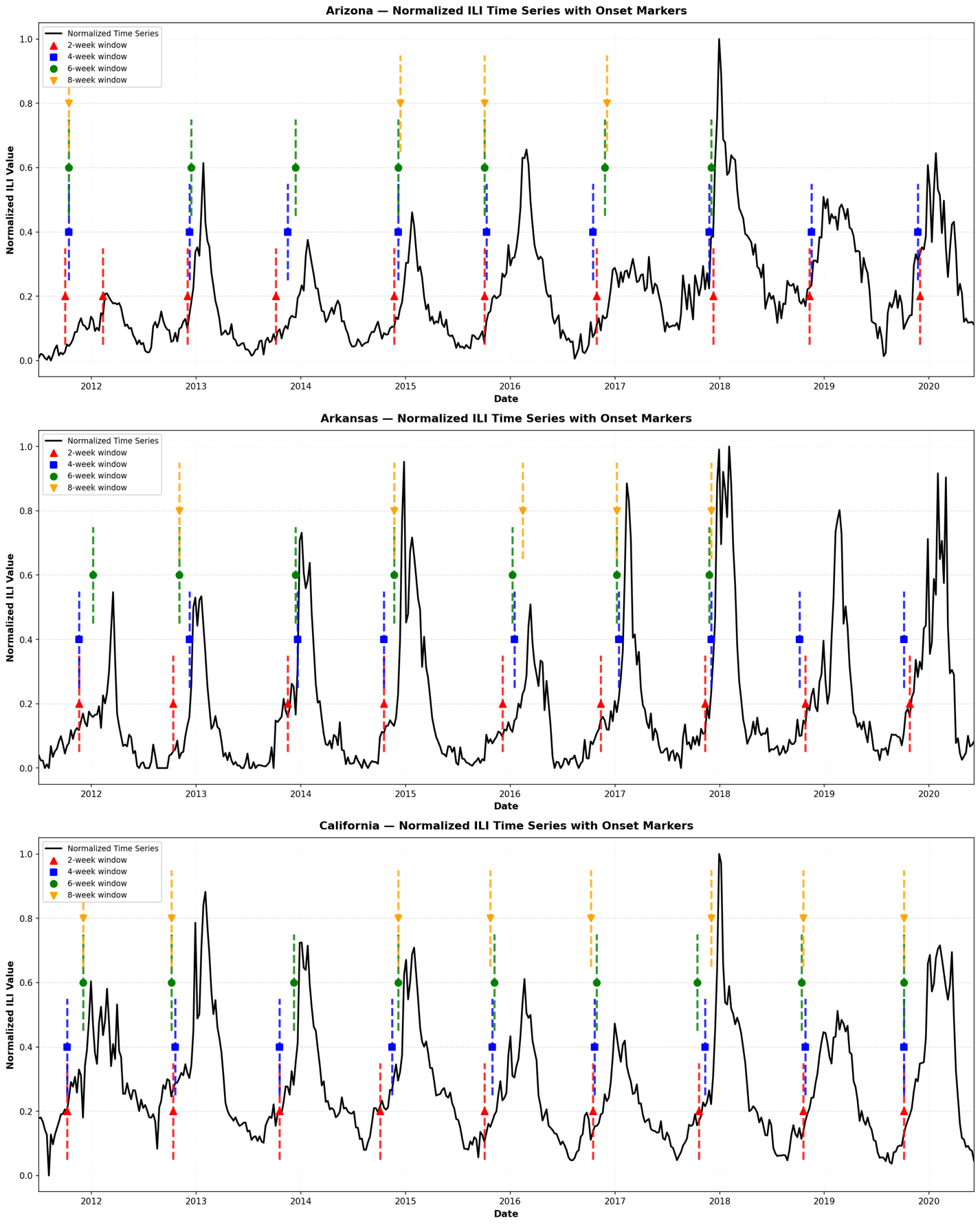 |
| --- |
| **Supplementary Figure 3**: **Sensitivity Analysis Results for Onset Detection Threshold for ILI (Arizona, Arkansas, California)**. Reducing the window size below 6 weeks results in increases in false positive rate, while increasing the window size to 8 weeks misses outbreaks. |

| 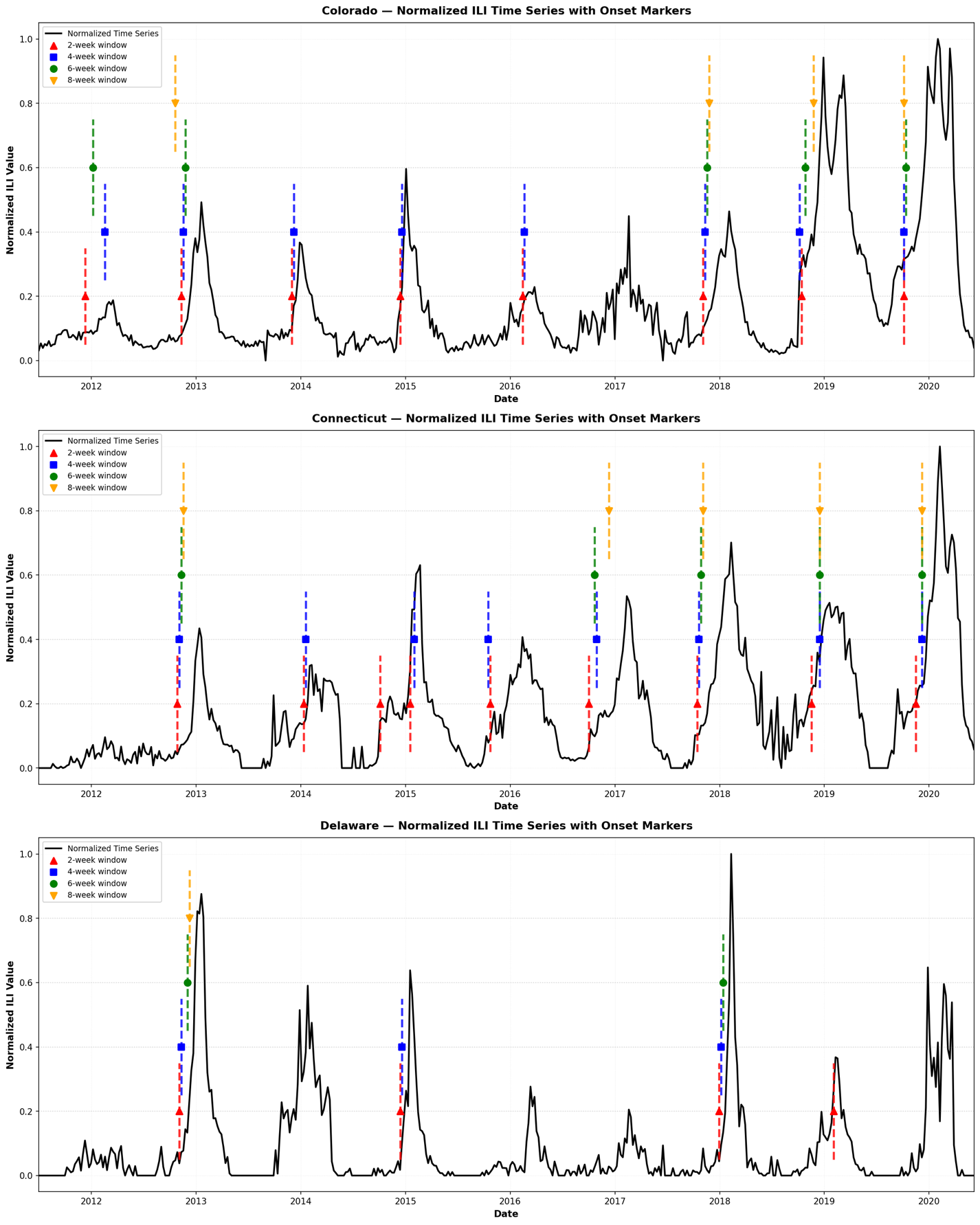 |
| --- |
| **Supplementary Figure 4**: **Sensitivity Analysis Results for Onset Detection Threshold for ILI (Colorado, Connecticut, Delaware)**. Reducing the window size below 6 weeks results in increases in false positive rate, while increasing the window size to 8 weeks misses outbreaks. |

| 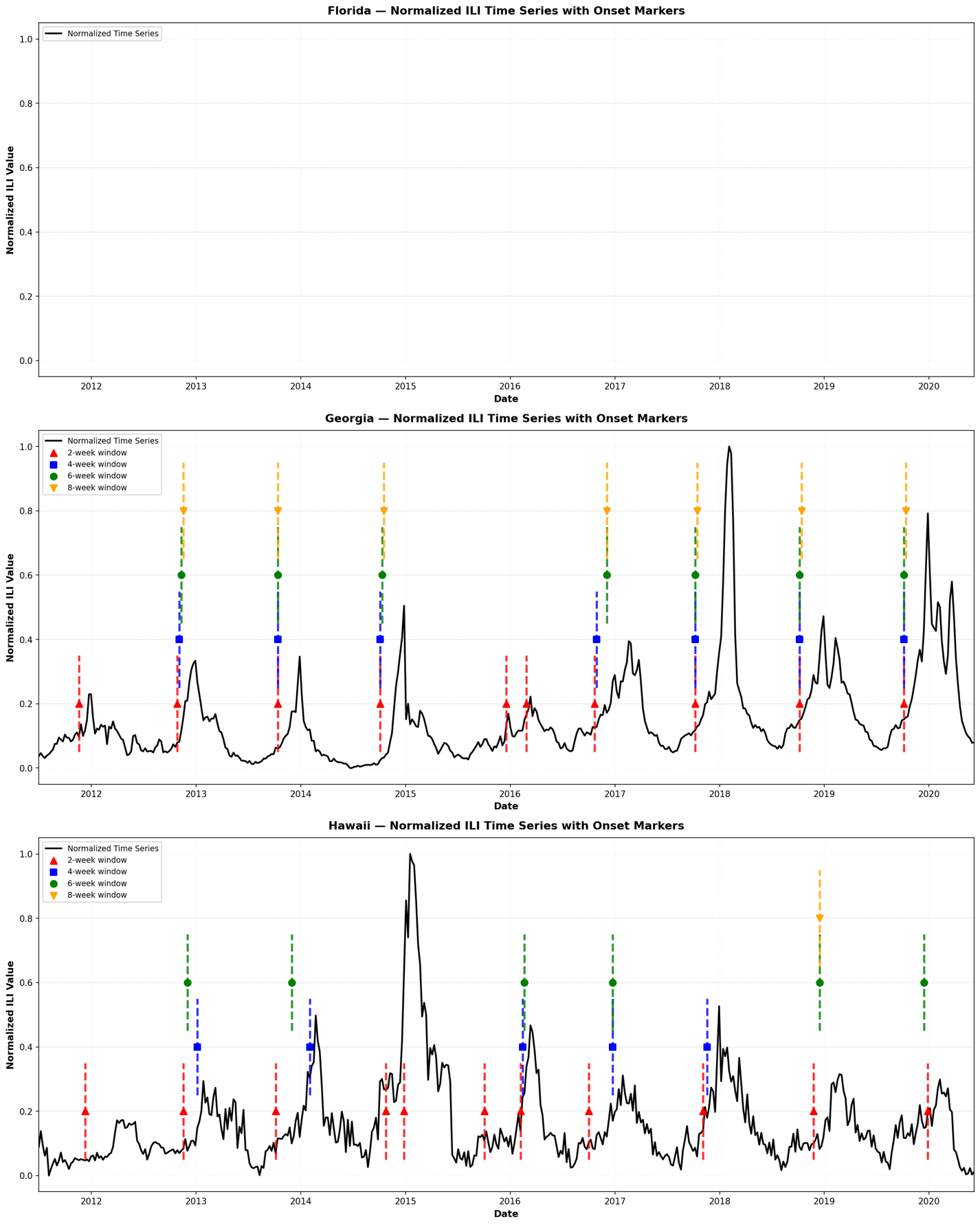 |
| --- |
| **Supplementary Figure 5**: **Sensitivity Analysis Results for Onset Detection Threshold for ILI (Florida, Georgia, Hawaii)** Reducing the window size below 6 weeks results in increases in false positive rate, while increasing the window size to 8 weeks misses outbreaks. |

| 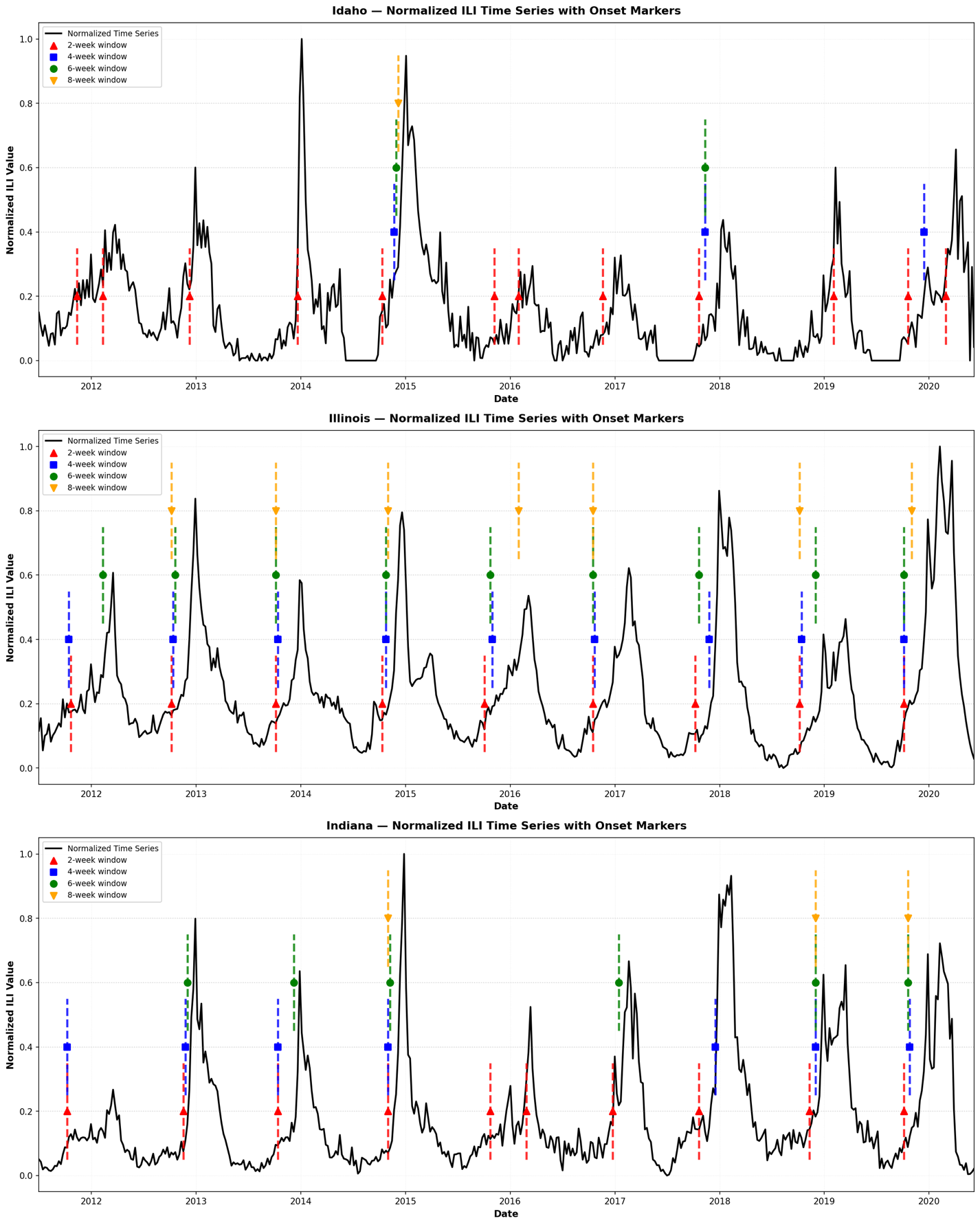 |
| --- |
| **Supplementary Figure 6**: **Sensitivity Analysis Results for Onset Detection Threshold for ILI (Idaho, Illinois, Indiana)**. Reducing the window size below 6 weeks results in increases in false positive rate, while increasing the window size to 8 weeks misses outbreaks. |

| 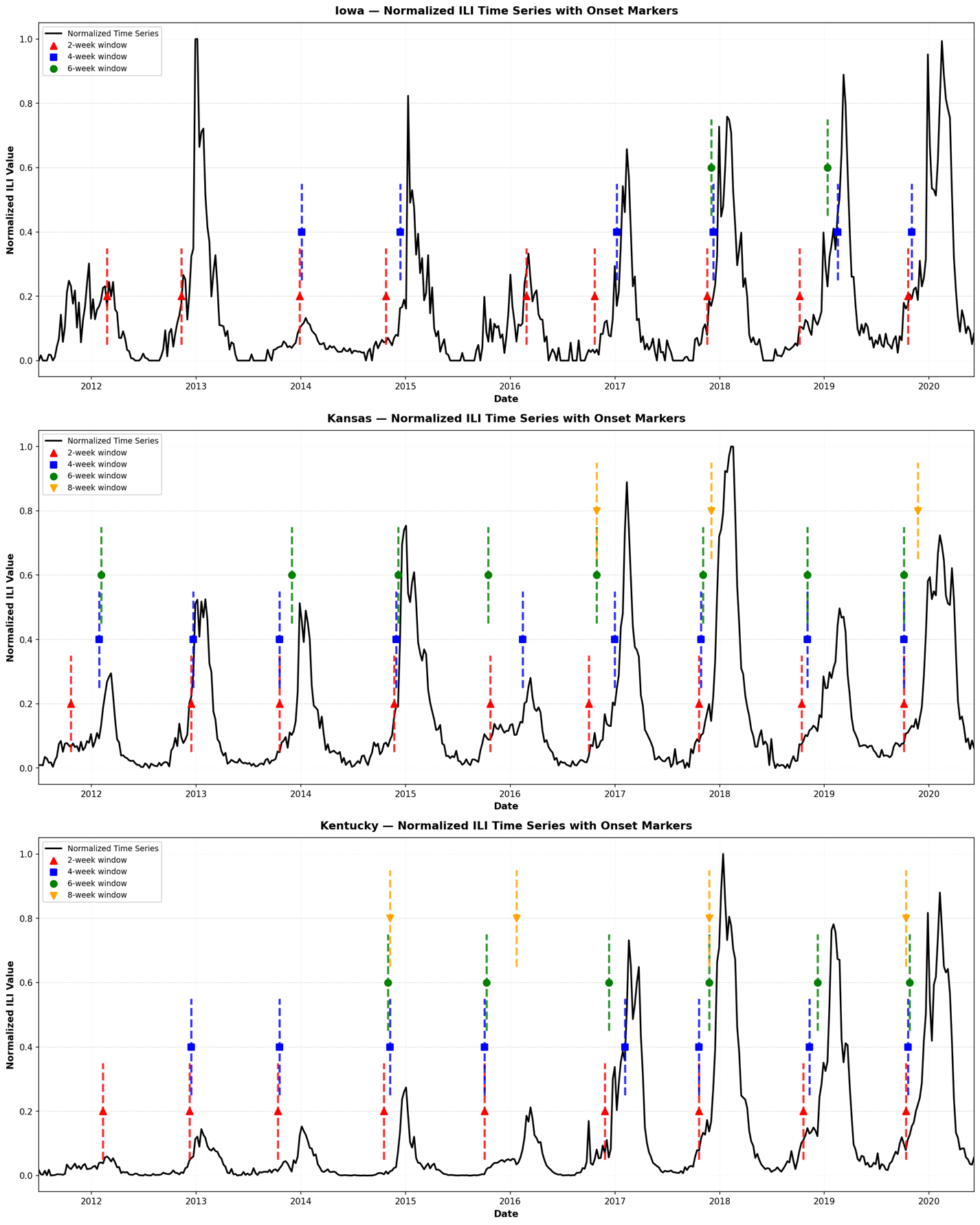 |
| --- |
| **Supplementary Figure 7**: **Sensitivity Analysis Results for Onset Detection Threshold for ILI (Iowa, Kansas, Kentucky)**. Reducing the window size below 6 weeks results in increases in false positive rate, while increasing the window size to 8 weeks misses outbreaks. |

| 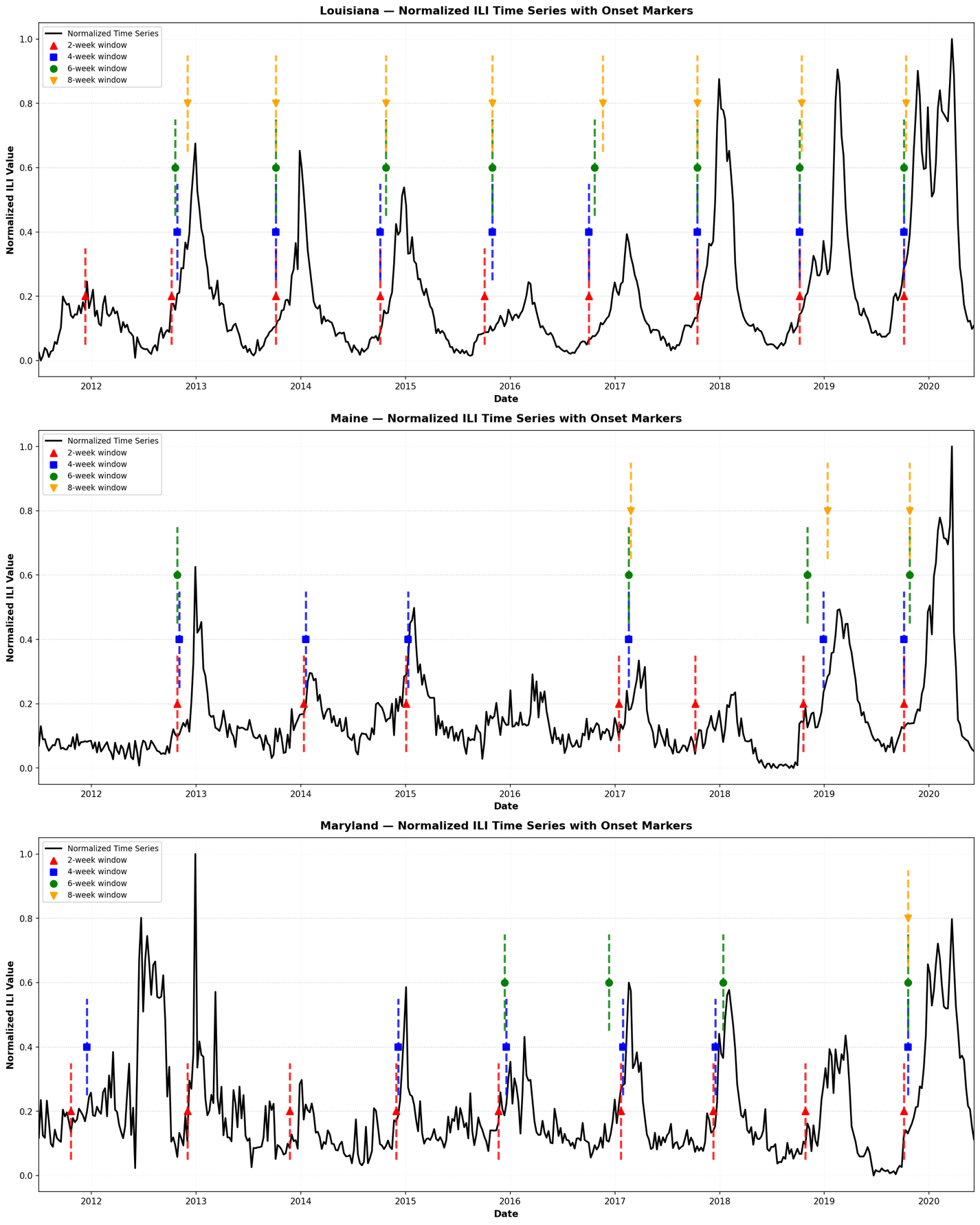 |
| --- |
| **Supplementary Figure 8**: **Sensitivity Analysis Results for Onset Detection Threshold for ILI (Louisiana, Maine, Maryland)**. Reducing the window size below 6 weeks results in increases in false positive rate, while increasing the window size to 8 weeks misses outbreaks. |

| 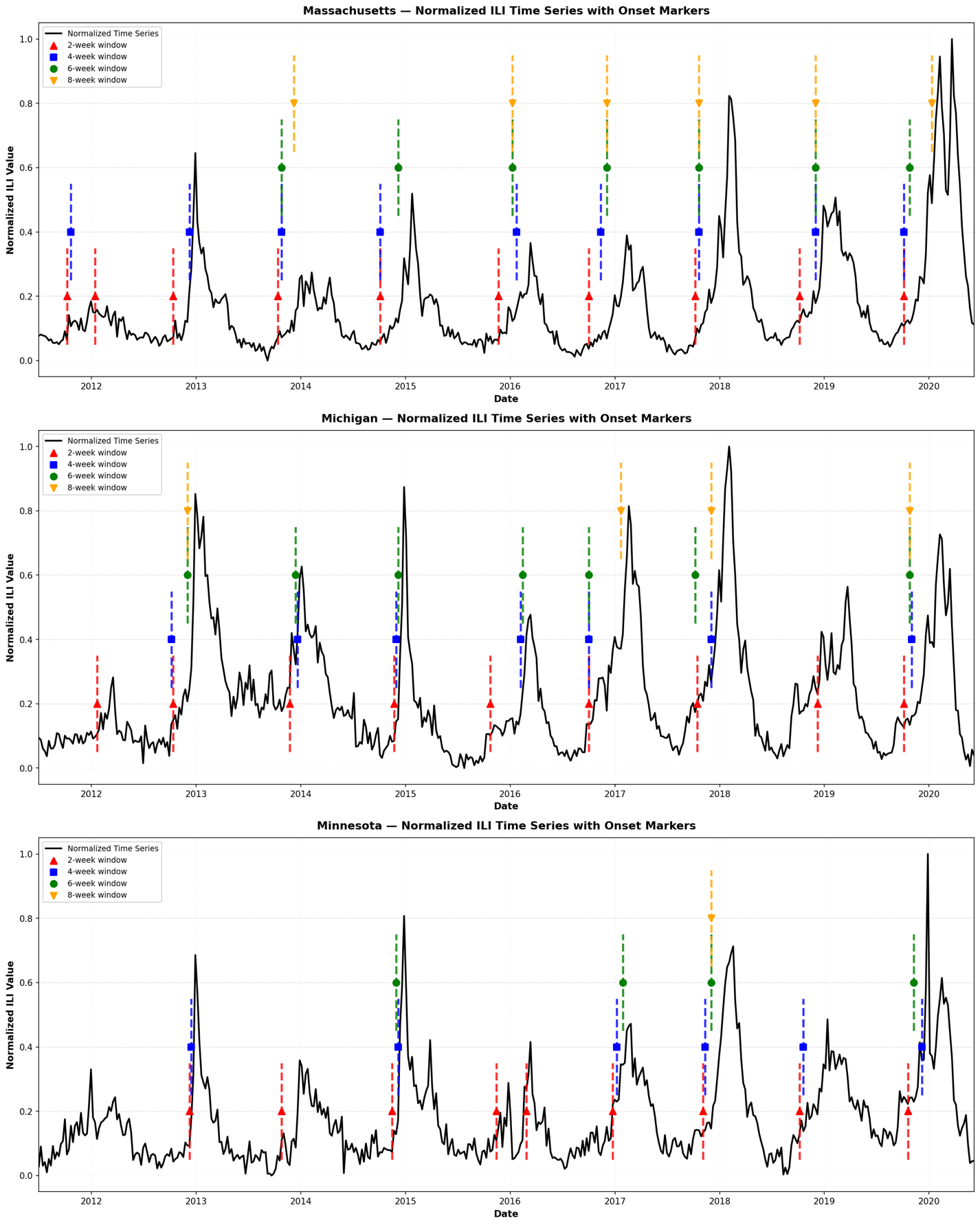 |
| --- |
| **Supplementary Figure 9**: **Sensitivity Analysis Results for Onset Detection Threshold for ILI (Massachusetts, Michigan, Minnesota)**. Reducing the window size below 6 weeks results in increases in false positive rate, while increasing the window size to 8 weeks misses outbreaks. |

| 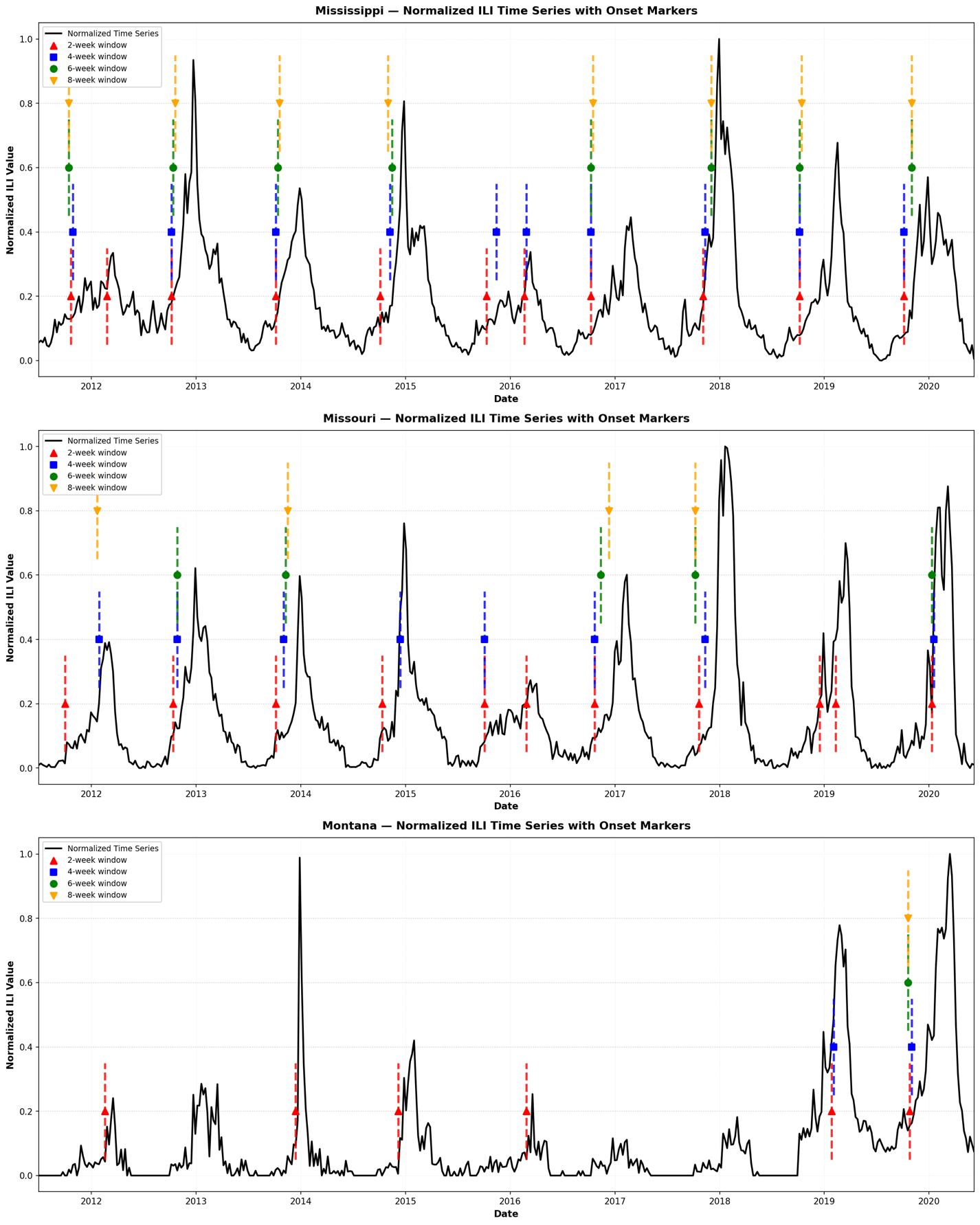 |
| --- |
| **Supplementary Figure 10**: **Sensitivity Analysis Results for Onset Detection Threshold for ILI (Mississippi, Missouri, Montana)**. Reducing the window size below 6 weeks results in increases in false positive rate, while increasing the window size to 8 weeks misses outbreaks. |

| 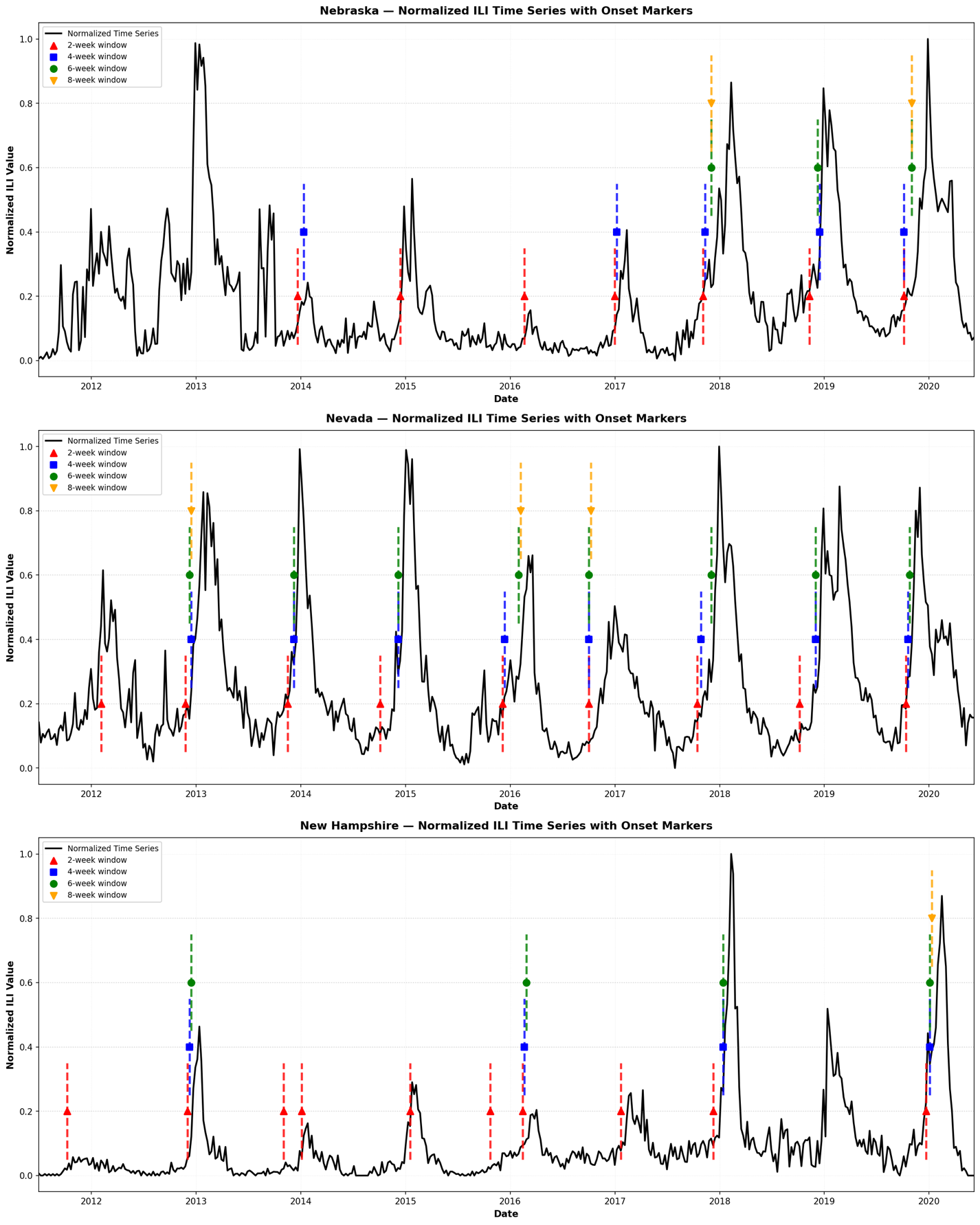 |
| --- |
| **Supplementary Figure 11**: **Sensitivity Analysis Results for Onset Detection Threshold for ILI (Nebraska, Nevada, New Hampshire)**. Reducing the window size below 6 weeks results in increases in false positive rate, while increasing the window size to 8 weeks misses outbreaks. |

| 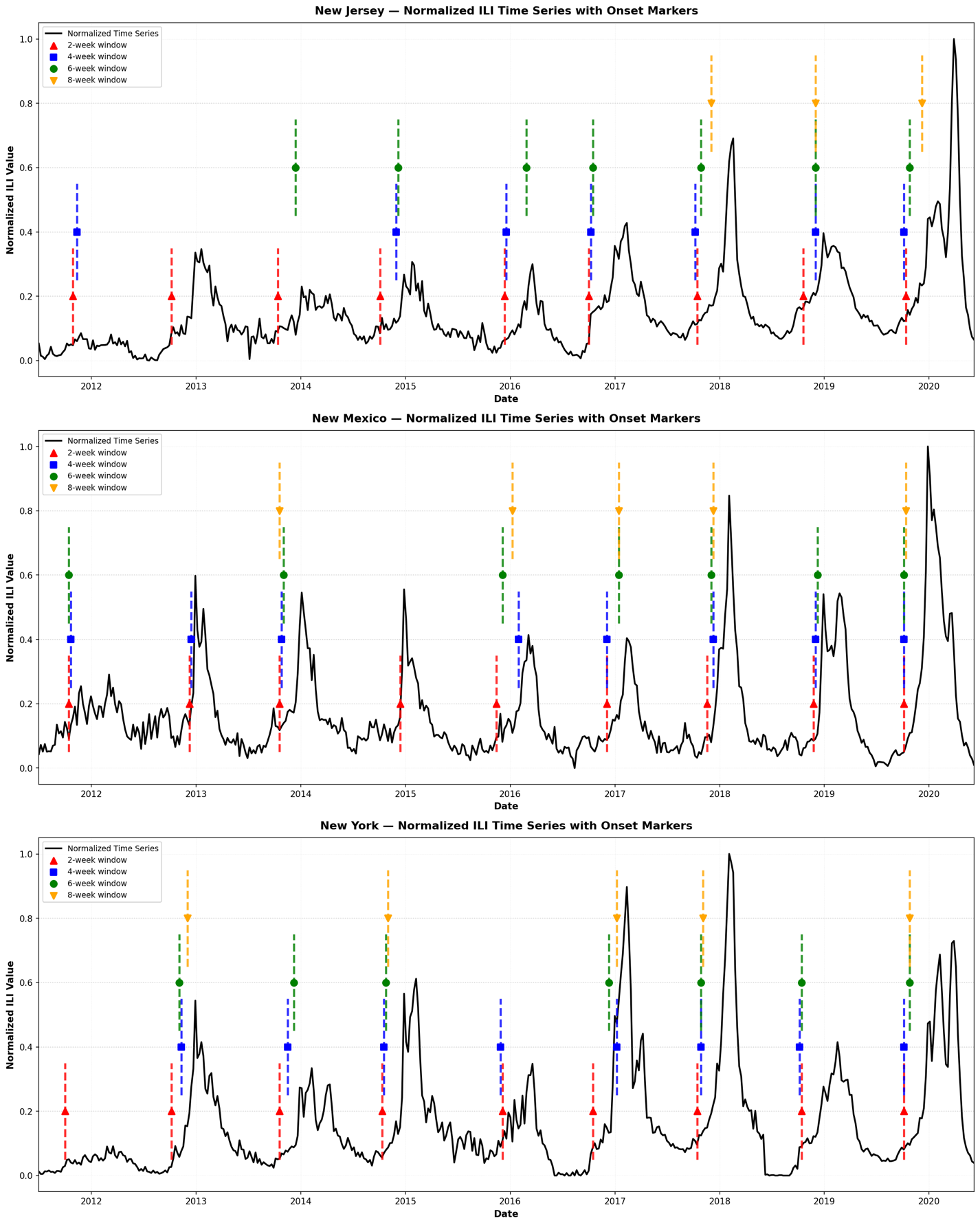 |
| --- |
| **Supplementary Figure 12**: **Sensitivity Analysis Results for Onset Detection Threshold for ILI (New Jersey, New Mexico, New York)**. Reducing the window size below 6 weeks results in increases in false positive rate, while increasing the window size to 8 weeks misses outbreaks. |

| 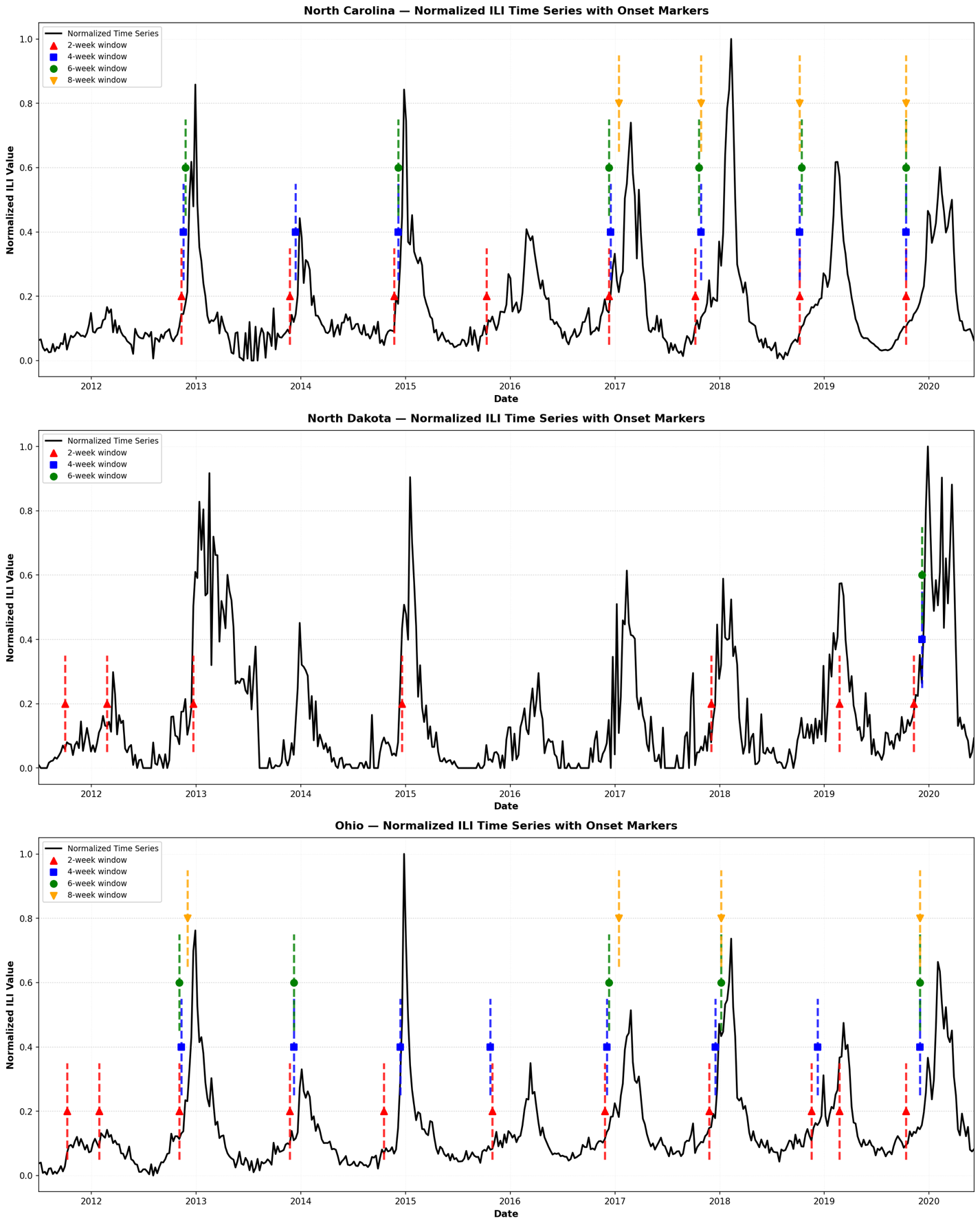 |
| --- |
| **Supplementary Figure 13**: **Sensitivity Analysis Results for Onset Detection Threshold for ILI (North Carolina, North Dakota, Ohio)**. Reducing the window size below 6 weeks results in increases in false positive rate, while increasing the window size to 8 weeks misses outbreaks. |

| 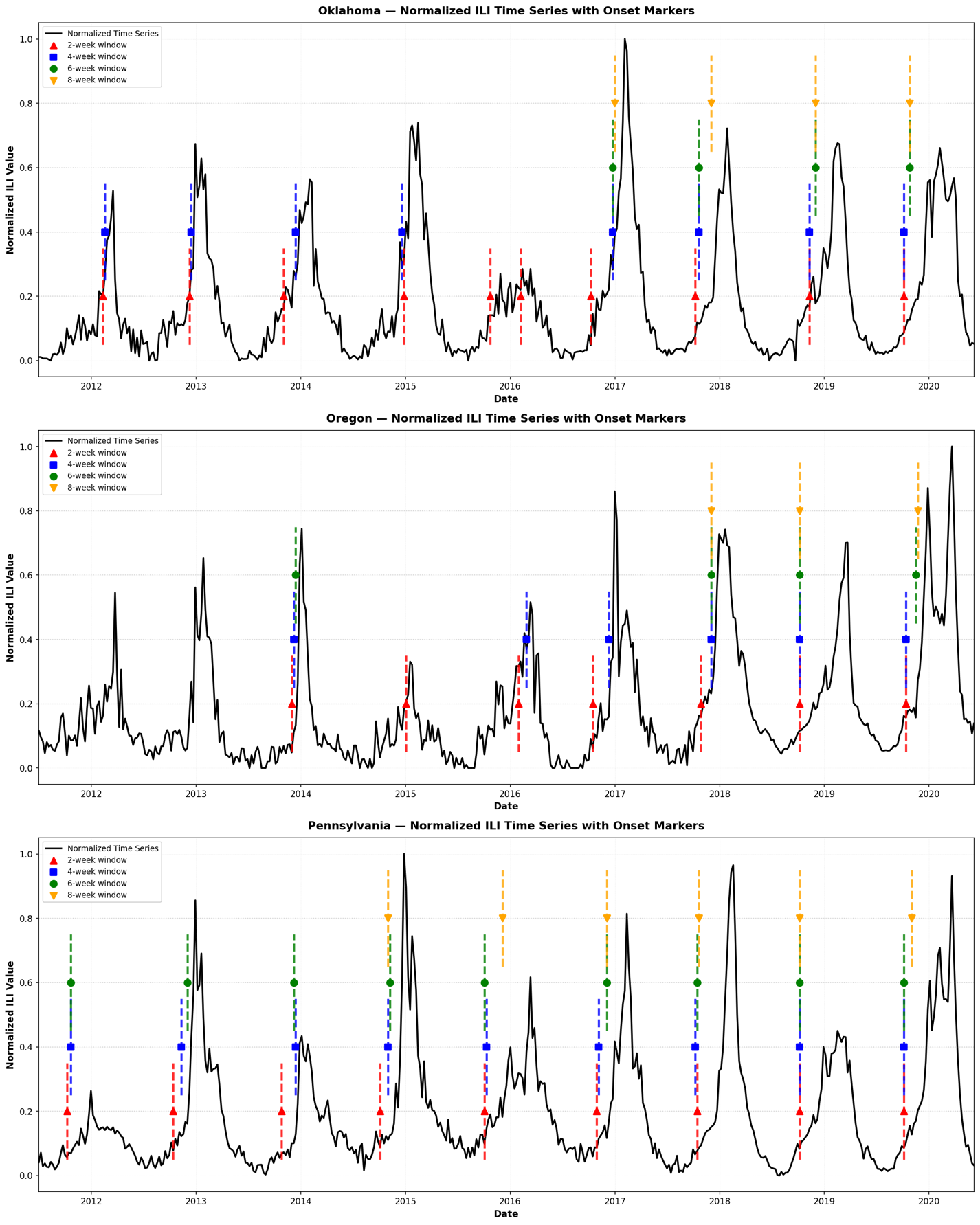 |
| --- |
|  |
| **Supplementary Figure 14**: **Sensitivity Analysis Results for Onset Detection Threshold for ILI (Oklahoma, Oregon, Pennsylvania)**. Reducing the window size below 6 weeks results in increases in false positive rate, while increasing the window size to 8 weeks misses outbreaks. |

| 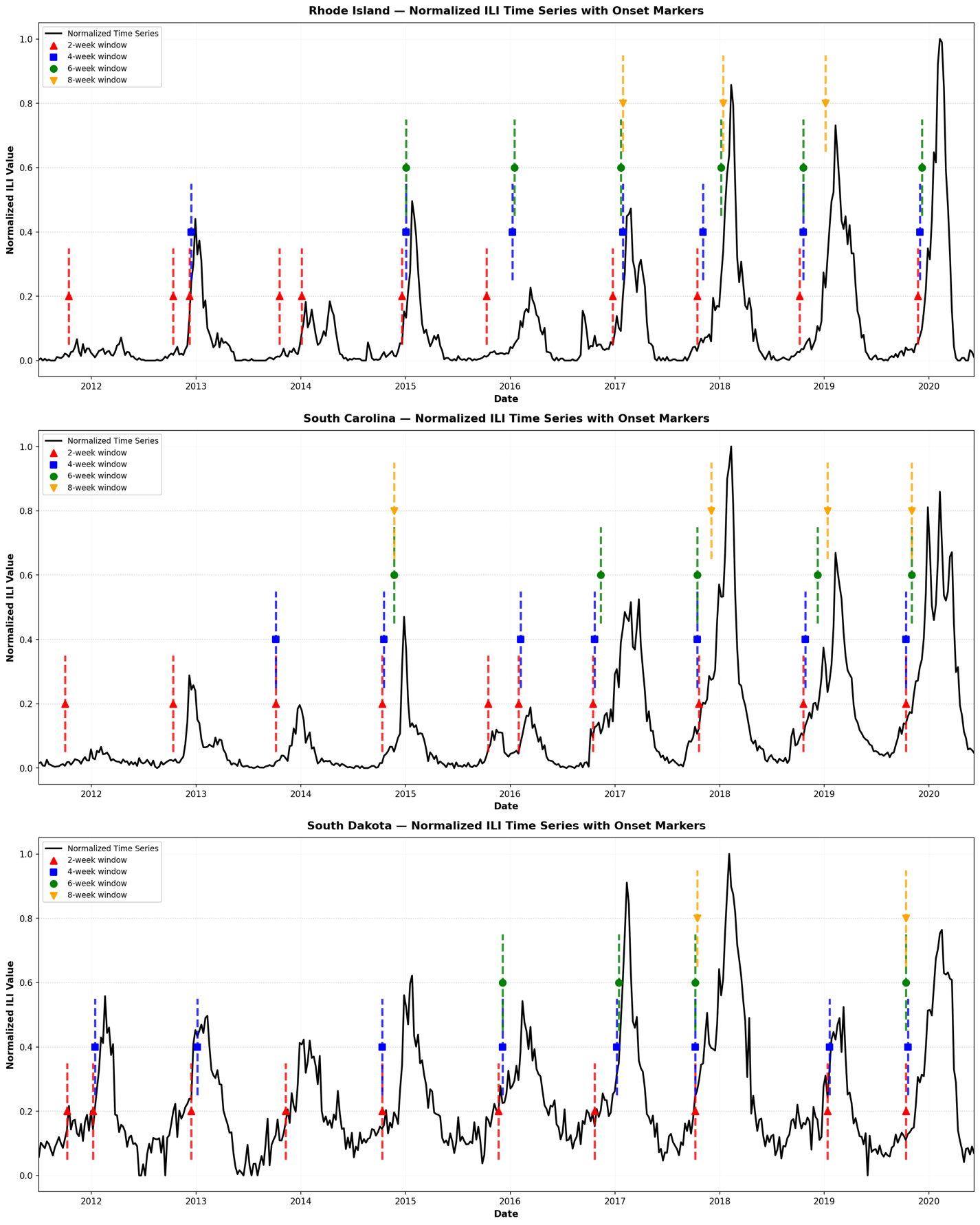 |
| --- |
| **Supplementary Figure 15**: **Sensitivity Analysis Results for Onset Detection Threshold for ILI (Rhode Island, South Carolina, South Dakota)**. Reducing the window size below 6 weeks results in increases in false positive rate, while increasing the window size to 8 weeks misses outbreaks. |

| 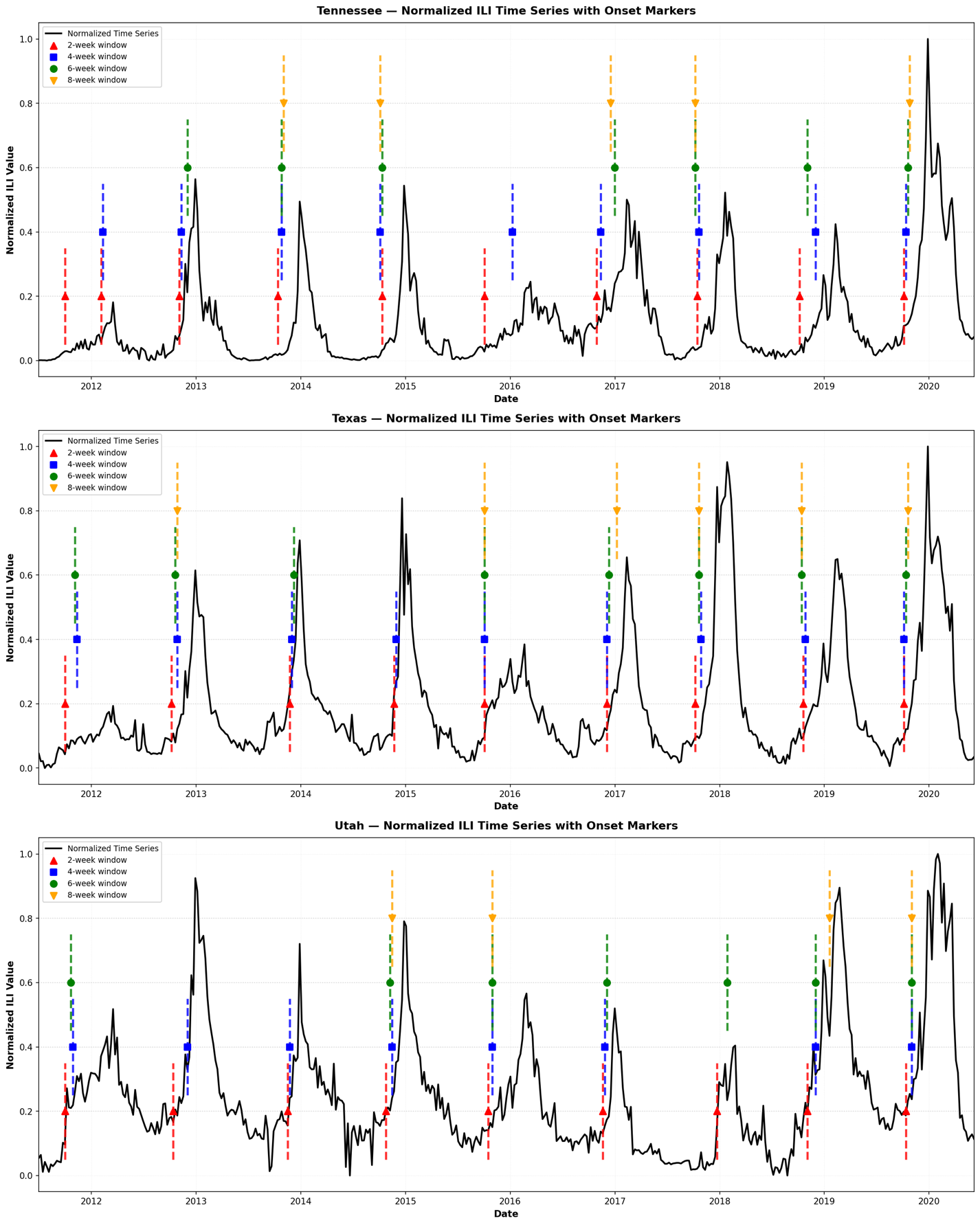 |
| --- |
| **Supplementary Figure 16**: **Sensitivity Analysis Results for Onset Detection Threshold for ILI (Tennessee, Texas, Utah)** Reducing the window size below 6 weeks results in increases in false positive rate, while increasing the window size to 8 weeks misses outbreaks. |

| 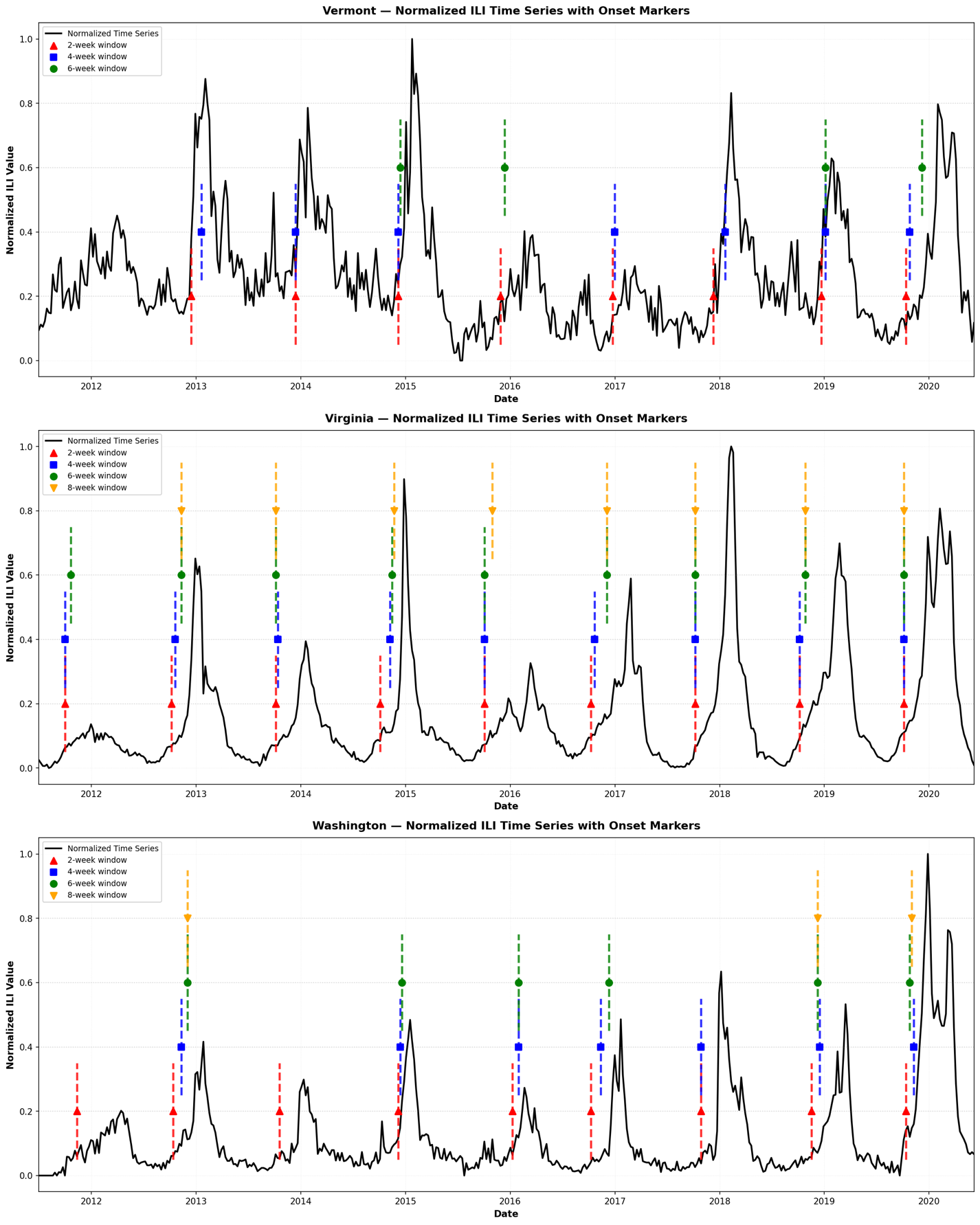 |
| --- |
| **Supplementary Figure 17**: **Sensitivity Analysis Results for Onset Detection Threshold for ILI (Vermont, Virginia, Washington)**. Reducing the window size below 6 weeks results in increases in false positive rate, while increasing the window size to 8 weeks misses outbreaks. |

| 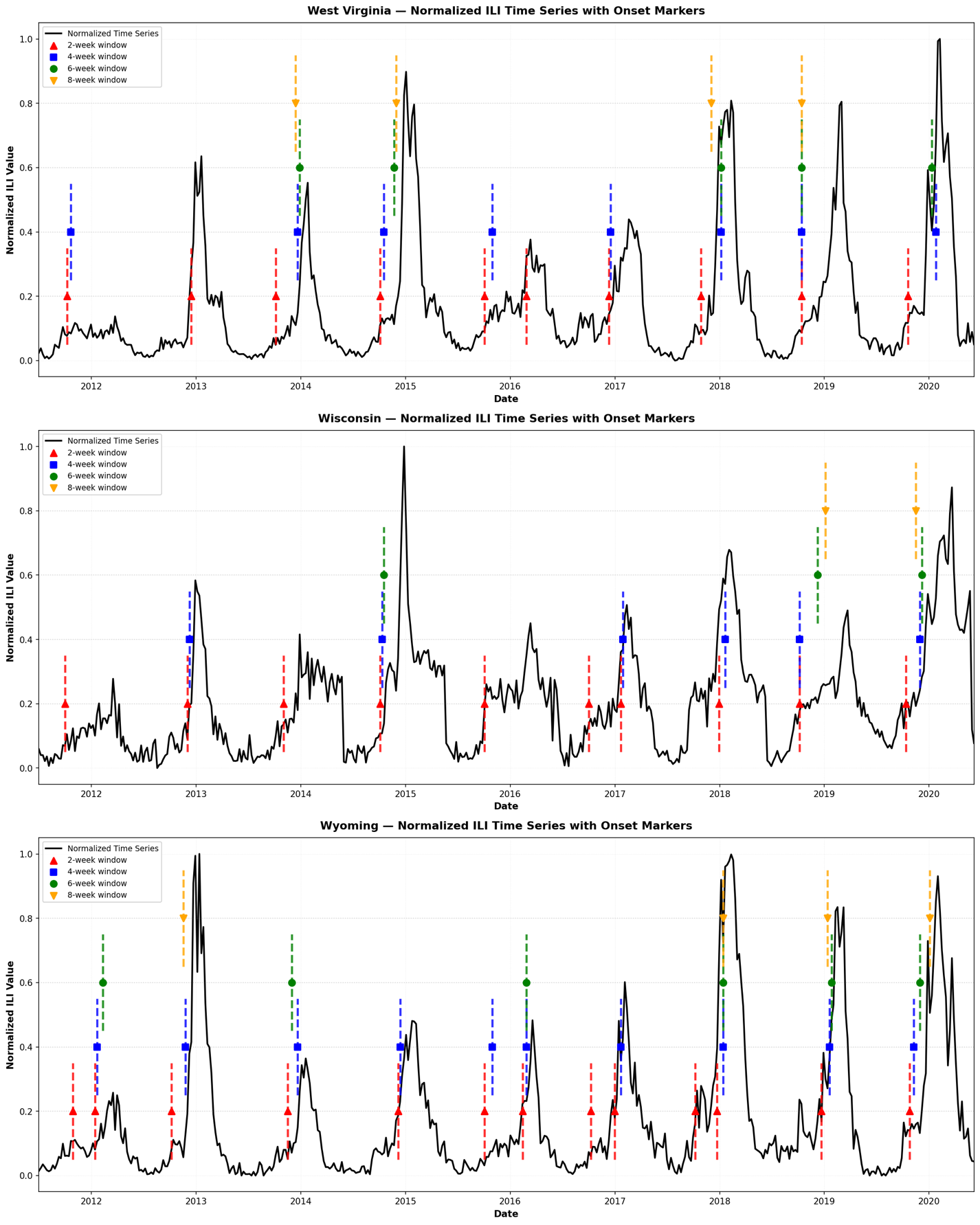 |
| --- |
| **Supplementary Figure 18**: **Sensitivity Analysis Results for Onset Detection Threshold for ILI (West Virginia, Wisconsin, Wyoming)**. Reducing the window size below 6 weeks results in increases in false positive rate, while increasing the window size to 8 weeks misses outbreaks. |

| 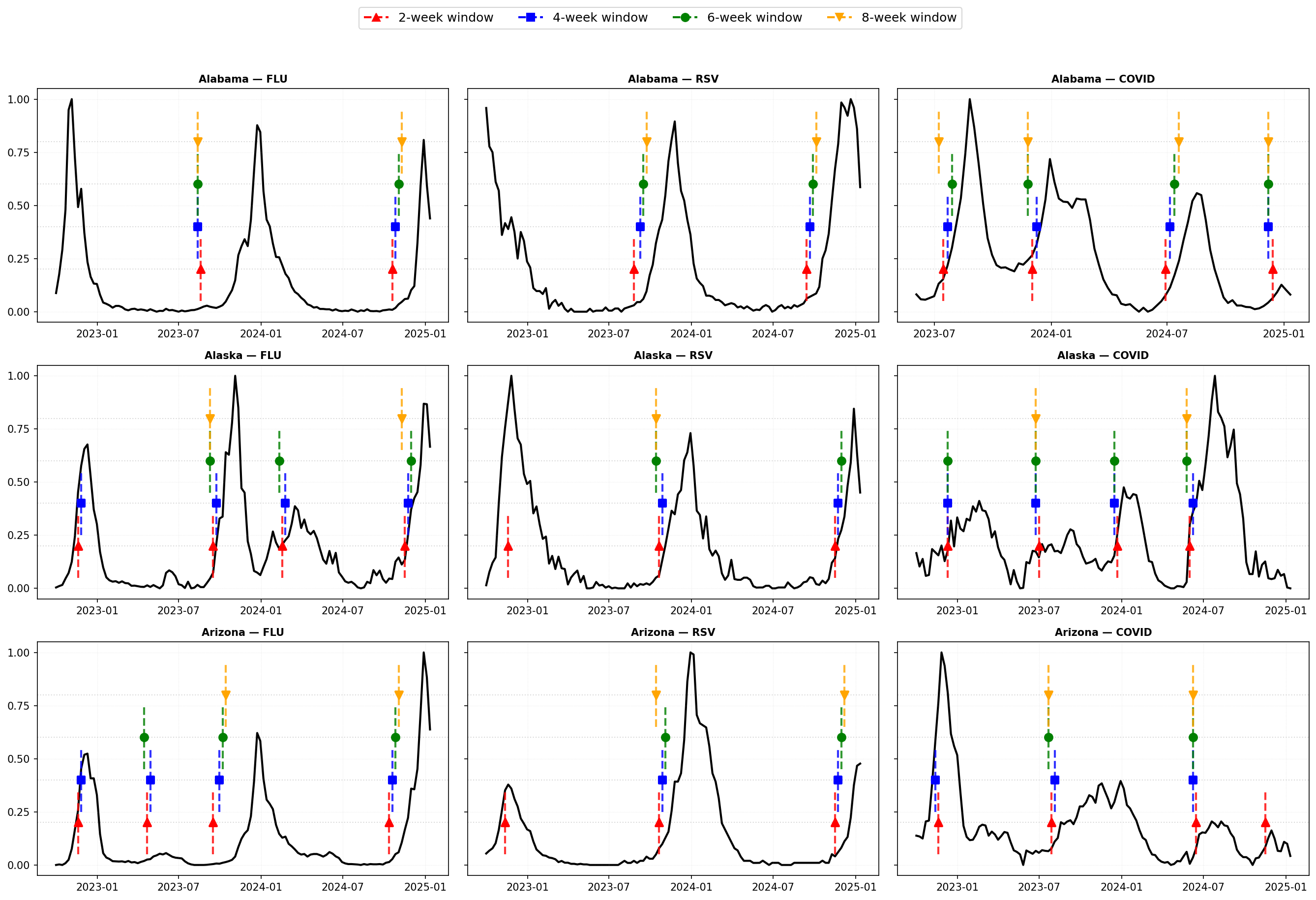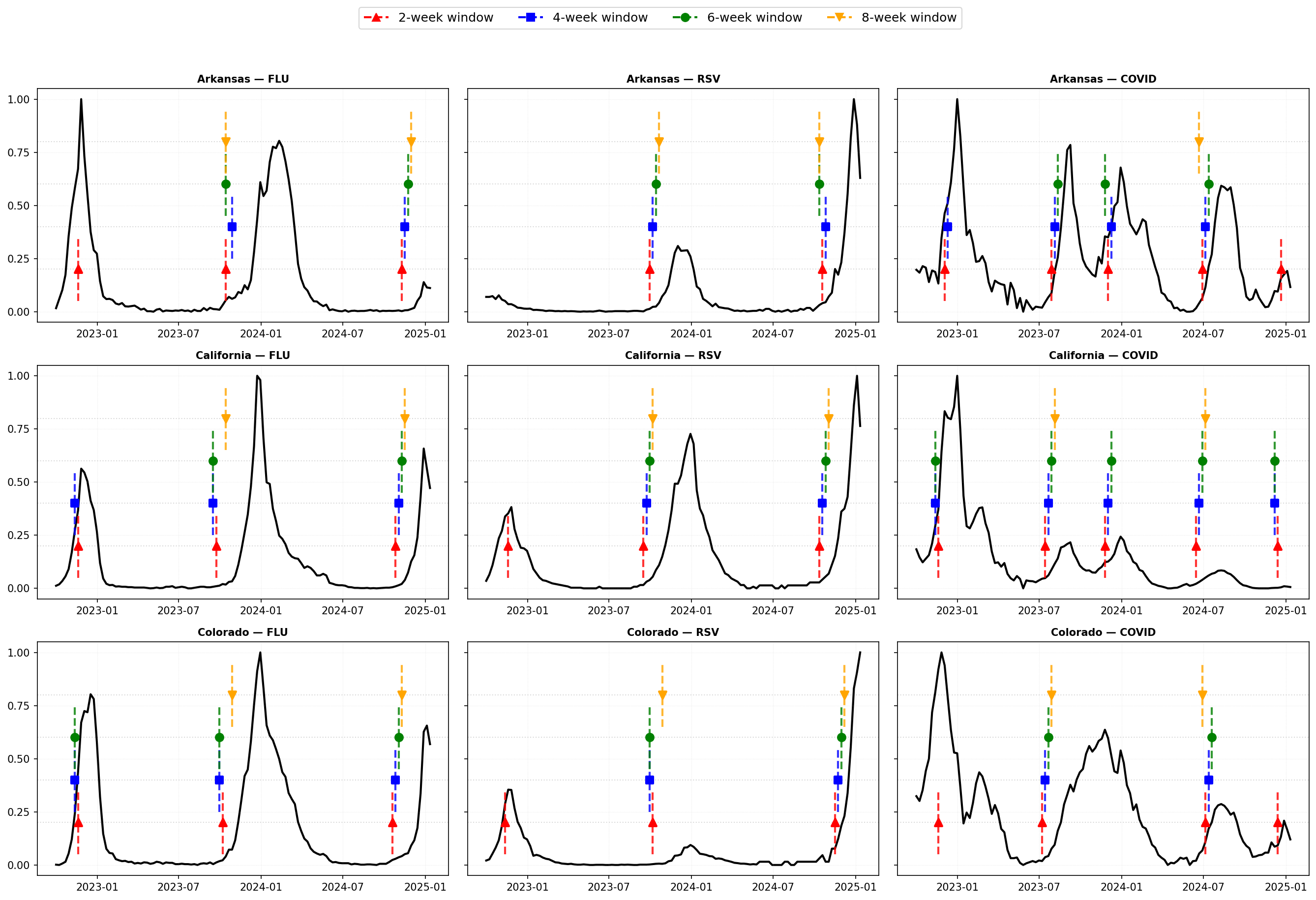 |
| --- |
| **Supplementary Figure 19**: **Sensitivity Analysis Results for Onset Detection Threshold for Predictors (Alabama, Alaska, Arizona, Arkansas, California, Colorado)**. Reducing the window size below 6 weeks results in increases in false positive rate, while increasing the window size to 8 weeks misses outbreaks. |

| 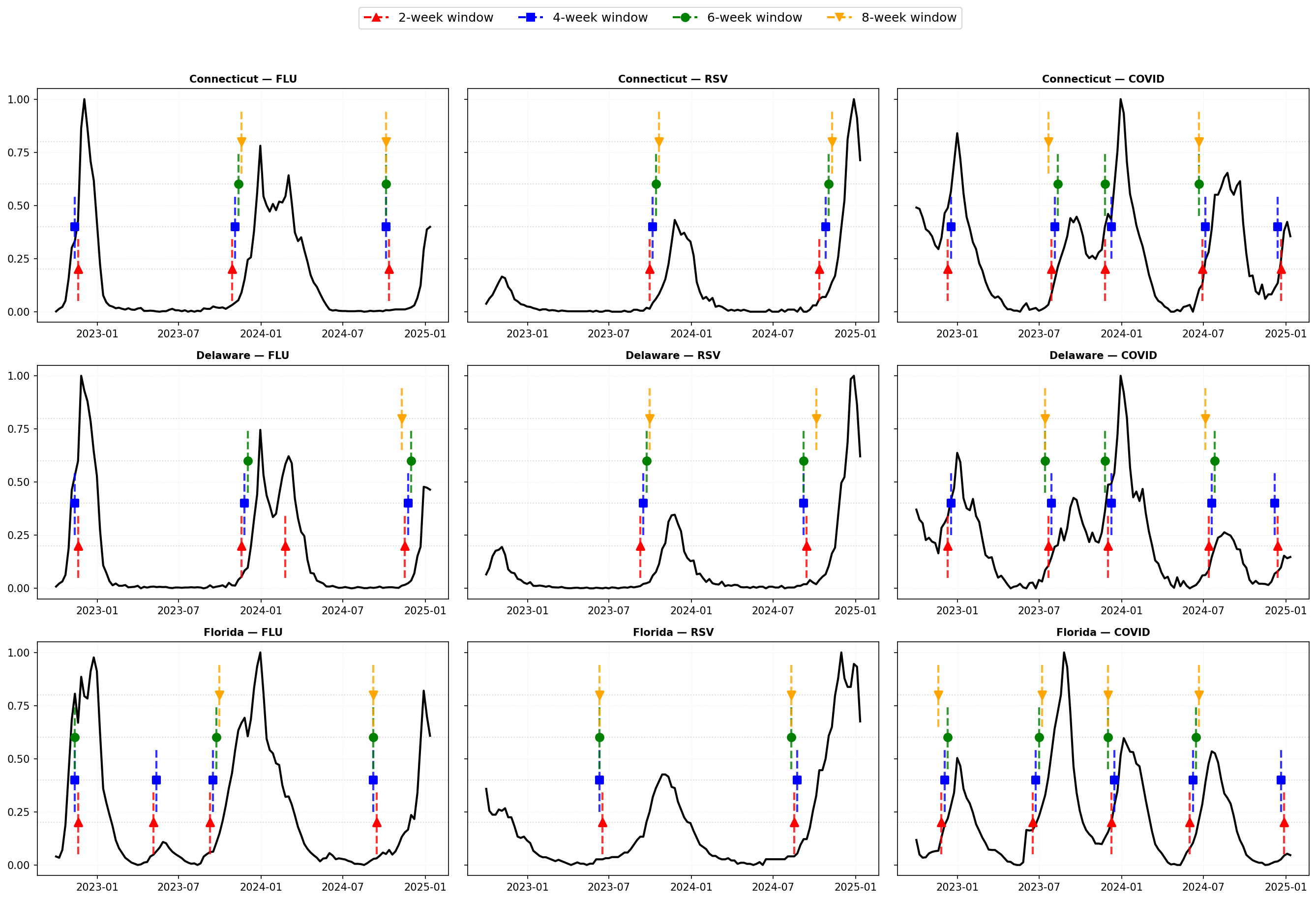 |
| --- |
| **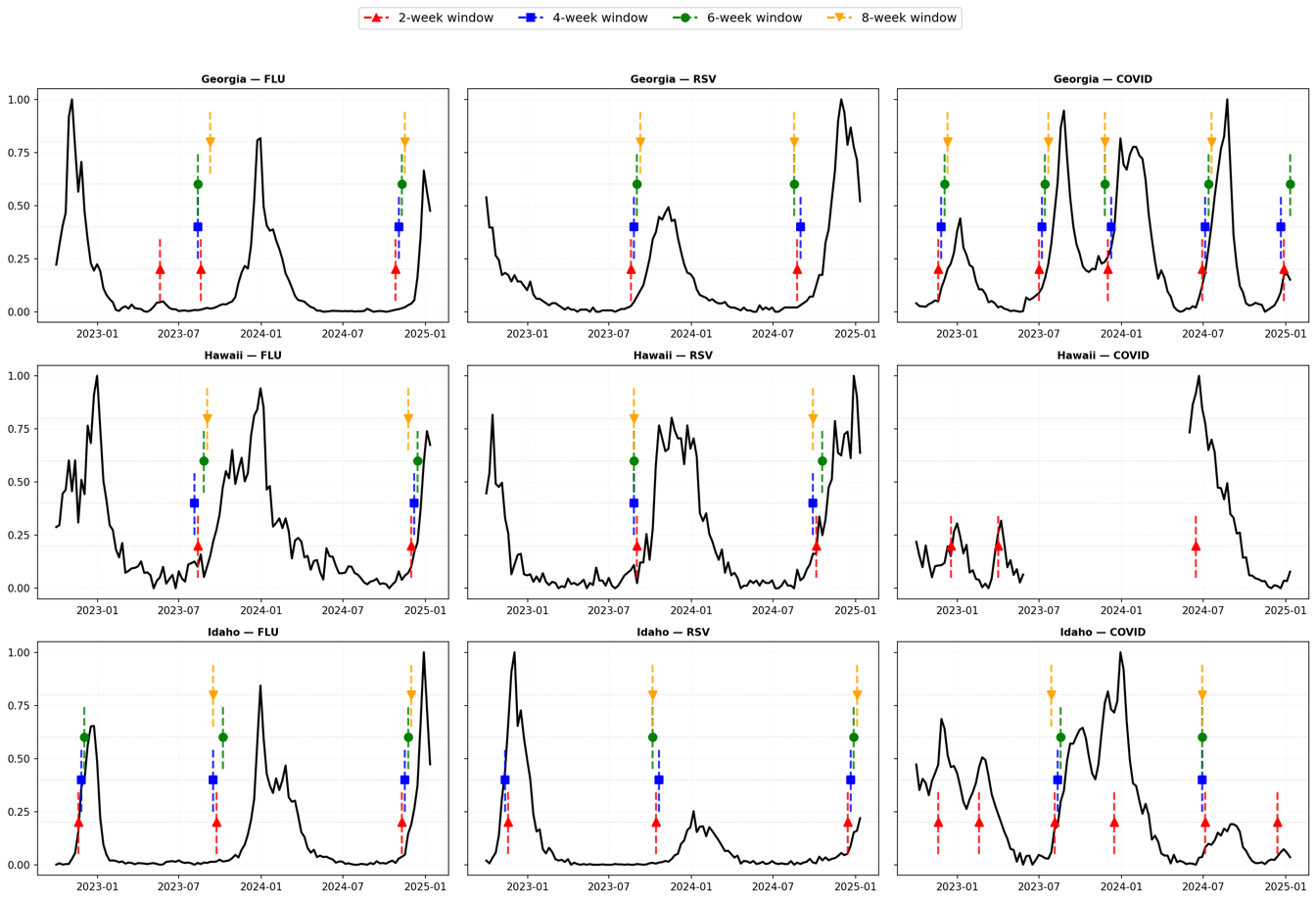**  **Supplementary Figure 20**: **Sensitivity Analysis Results for Onset Detection Threshold for Predictors (Connecticut, Delaware, Florida, Georgia, Hawaii, Idaho)**. Reducing the window size below 6 weeks results in increases in false positive rate, while increasing the window size to 8 weeks misses outbreaks. |

| 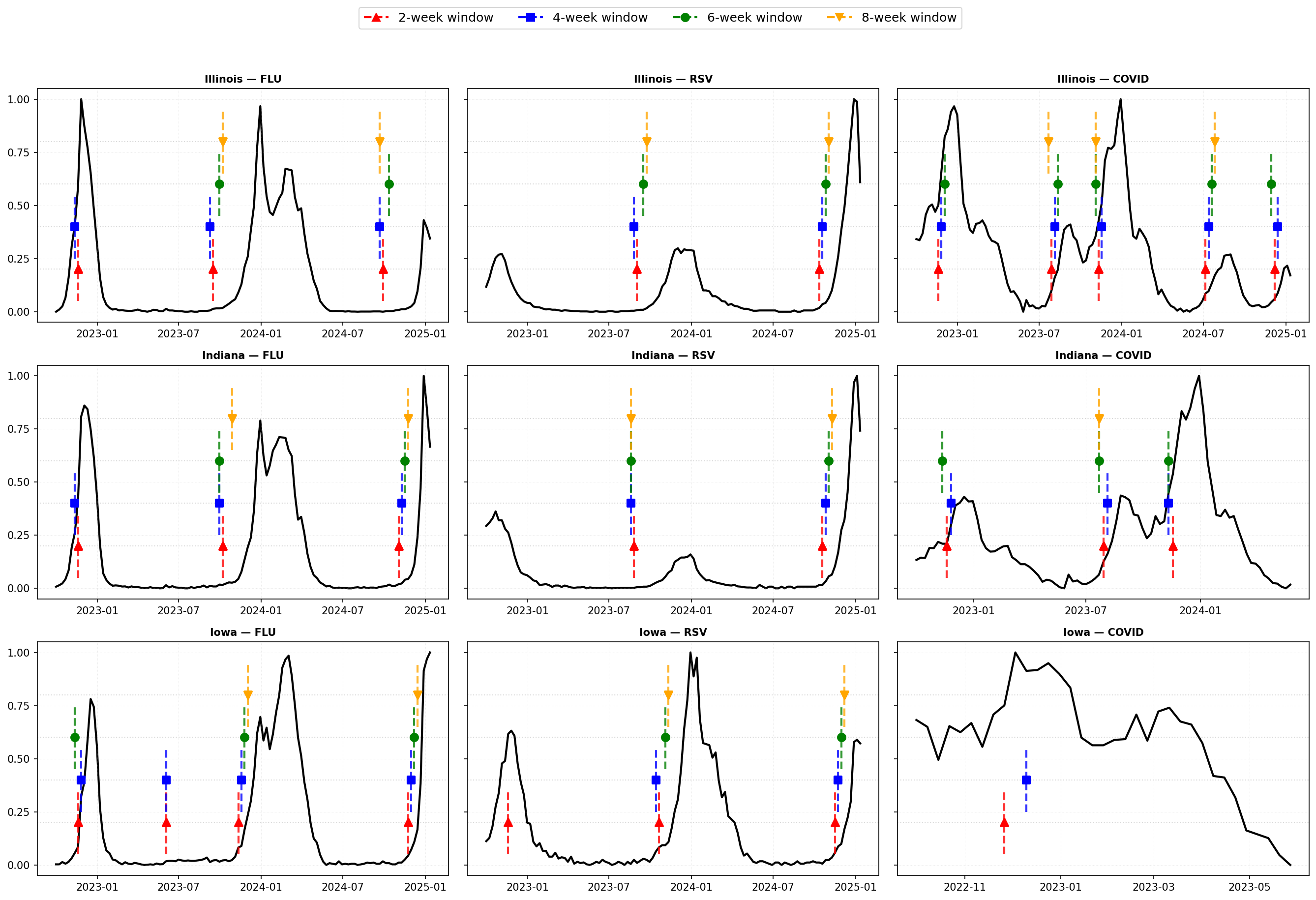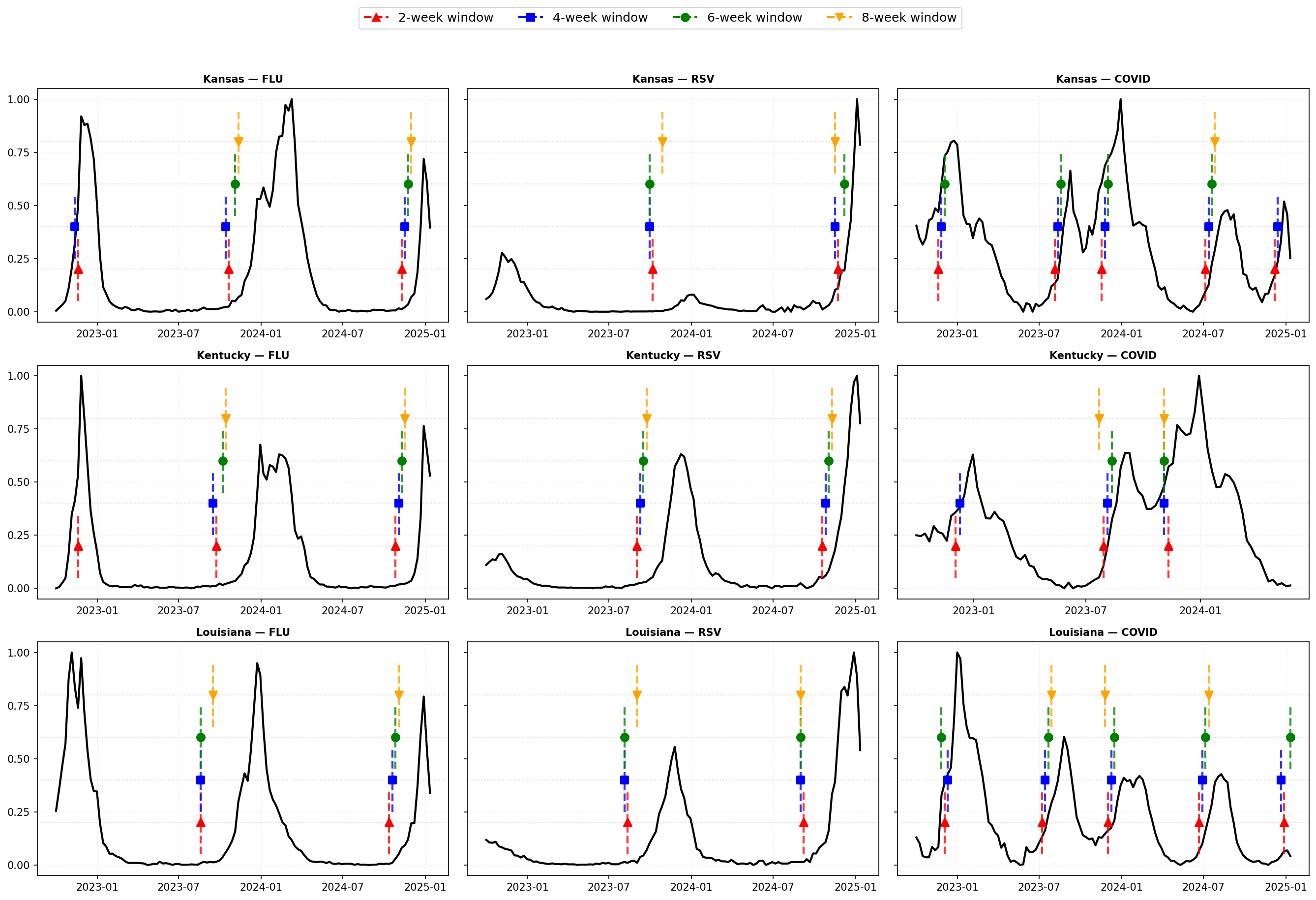 |
| --- |
| **Supplementary Figure 21**: **Sensitivity Analysis Results for Onset Detection Threshold for Predictors (Illinois, Indiana, Iowa, Kansas, Kentucky, Louisiana)**. Reducing the window size below 6 weeks results in increases in false positive rate, while increasing the window size to 8 weeks misses outbreaks. |

| 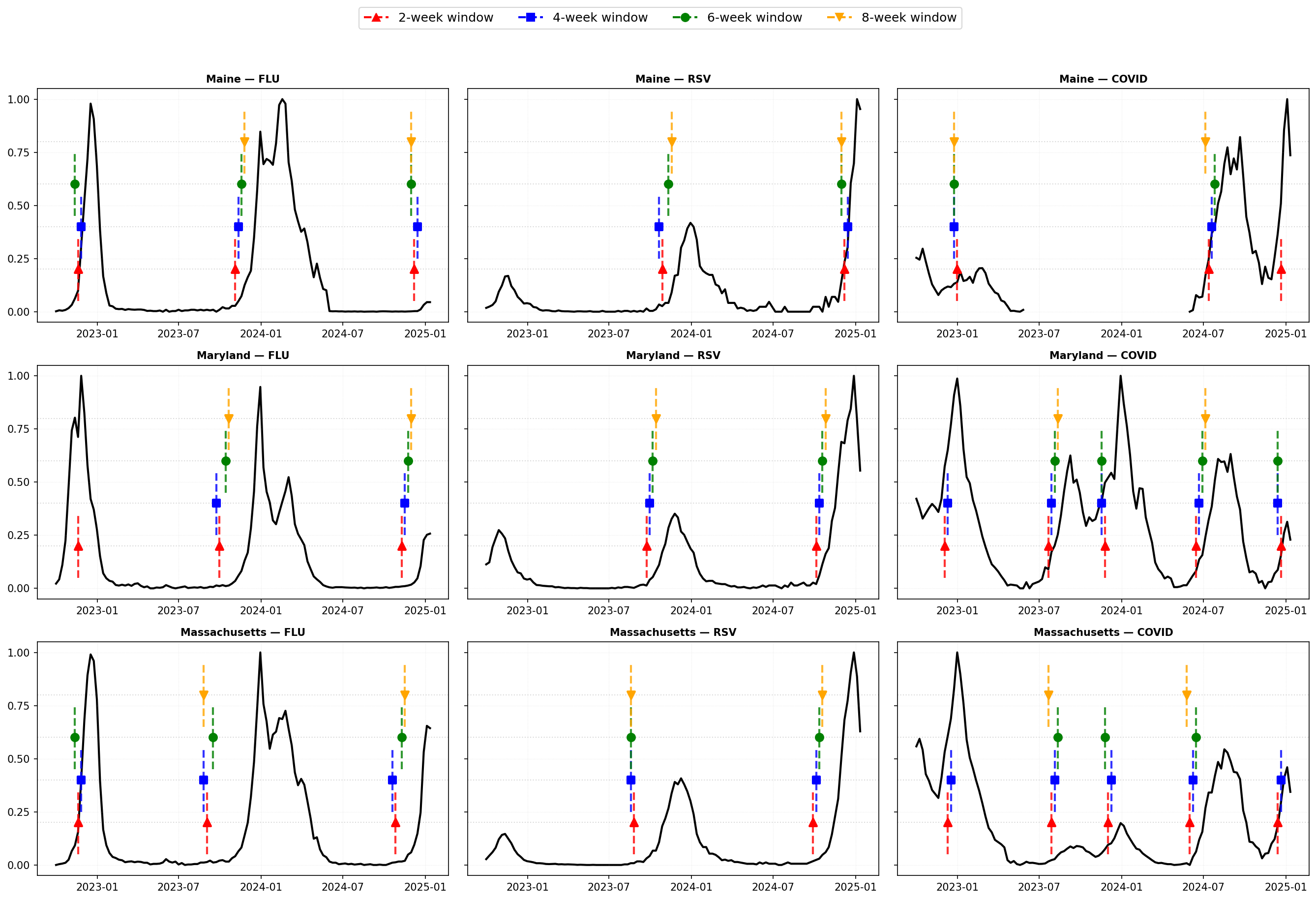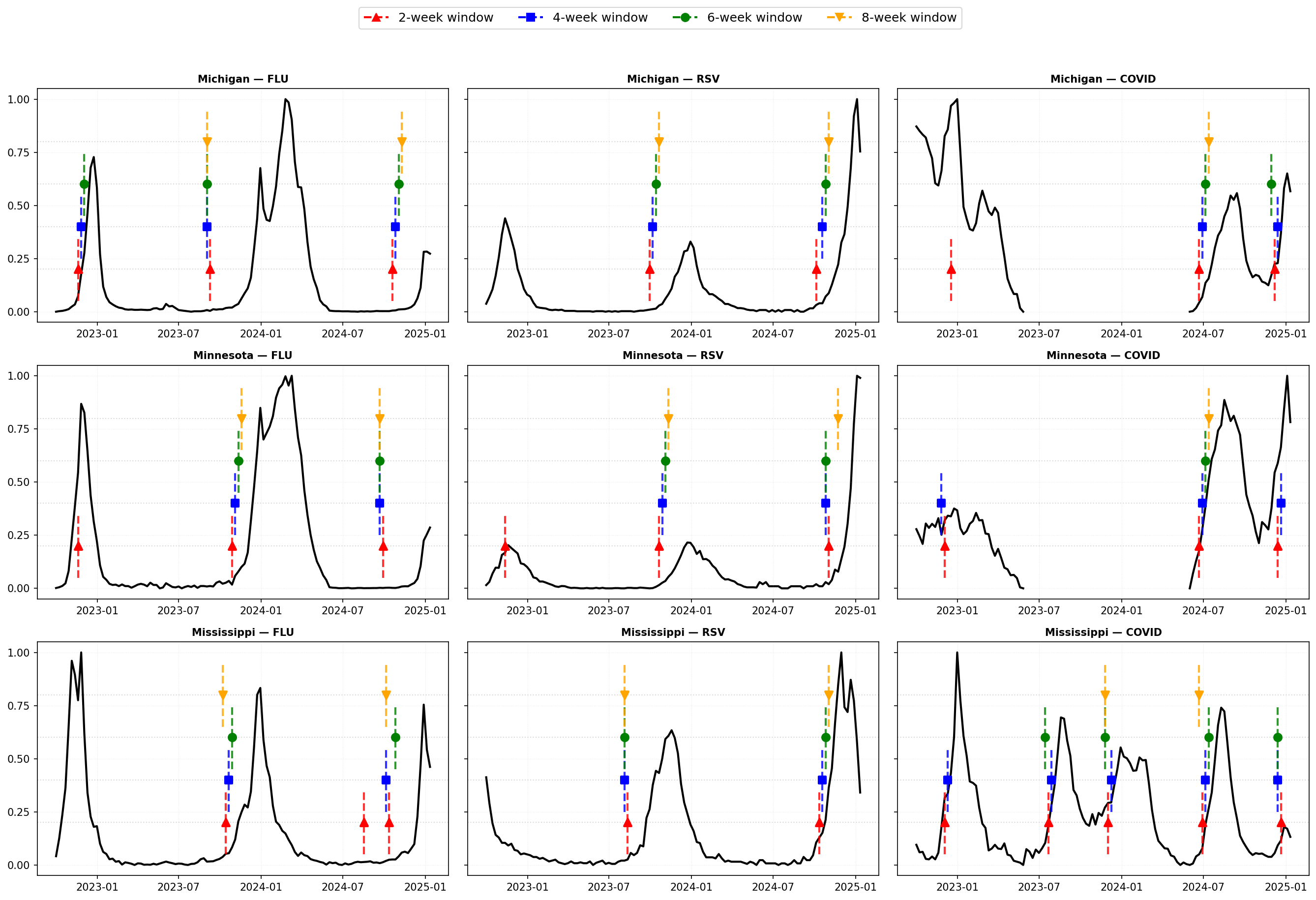 |
| --- |
| **Supplementary Figure 21**: **Sensitivity Analysis Results for Onset Detection Threshold for Predictors (Maine, Maryland, Massachusetts, Michigan, Minnesota, Mississippi)**. Reducing the window size below 6 weeks results in increases in false positive rate, while increasing the window size to 8 weeks misses outbreaks. |

| 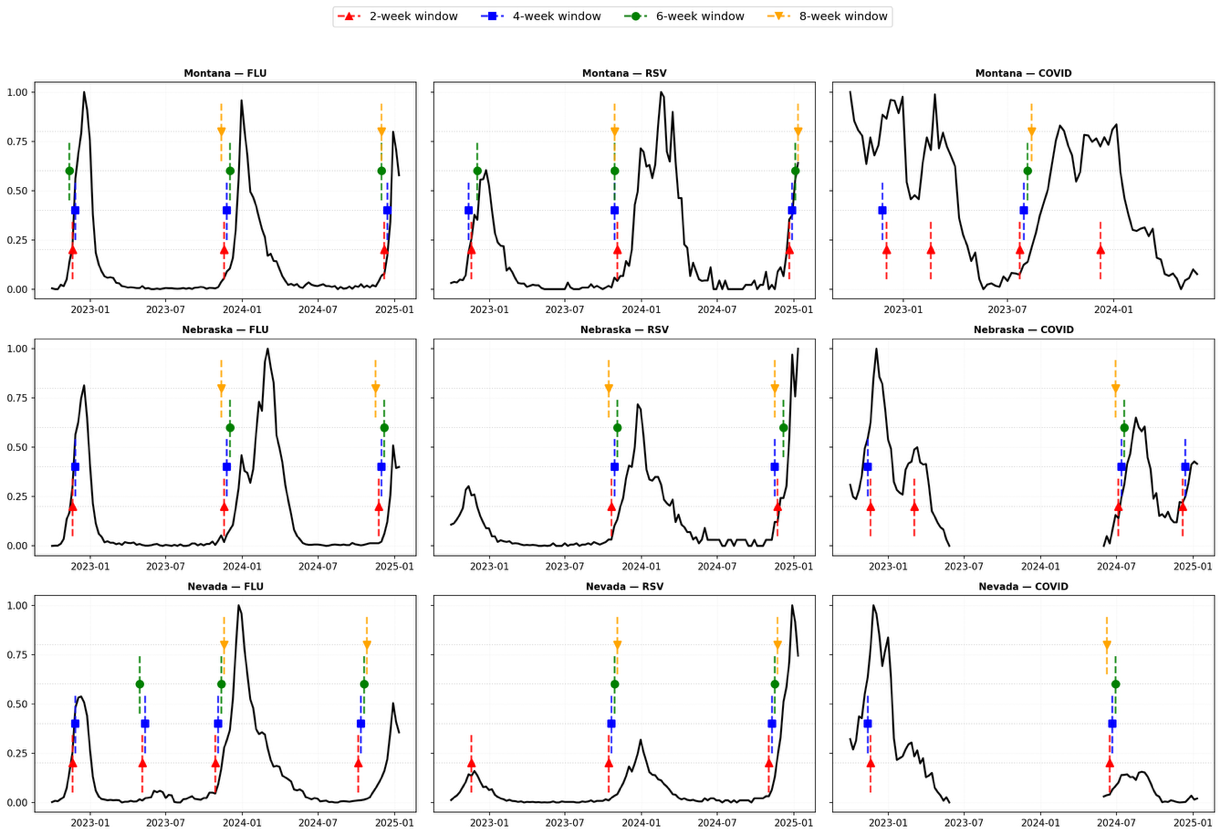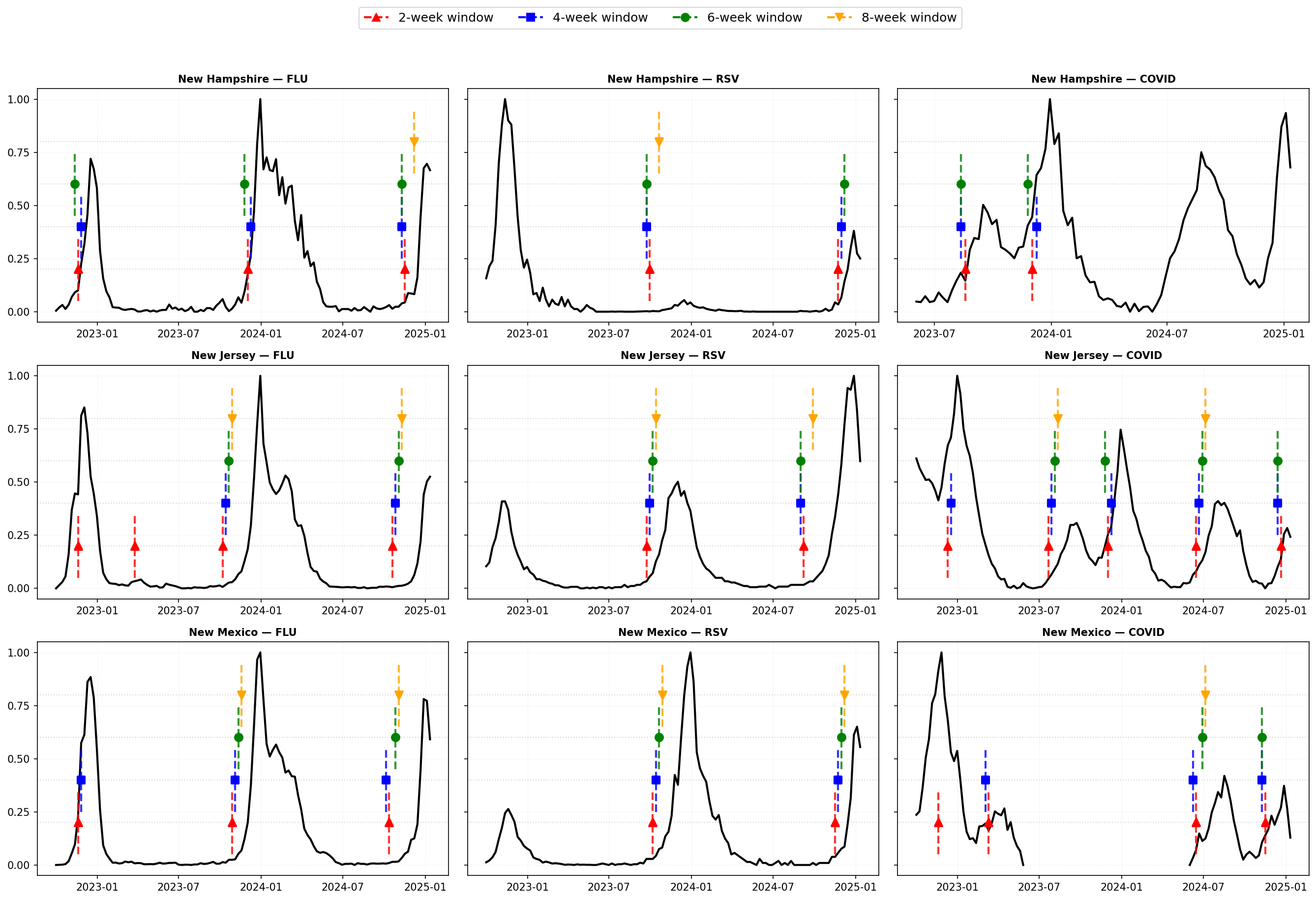 |
| --- |
| **Supplementary Figure 22**: **Sensitivity Analysis Results for Onset Detection Threshold for Predictors (Montana, Nebraska, Nevada, New Hampshire, New Jersey, New Mexico)**. Reducing the window size below 6 weeks results in increases in false positive rate, while increasing the window size to 8 weeks misses outbreaks. |

| 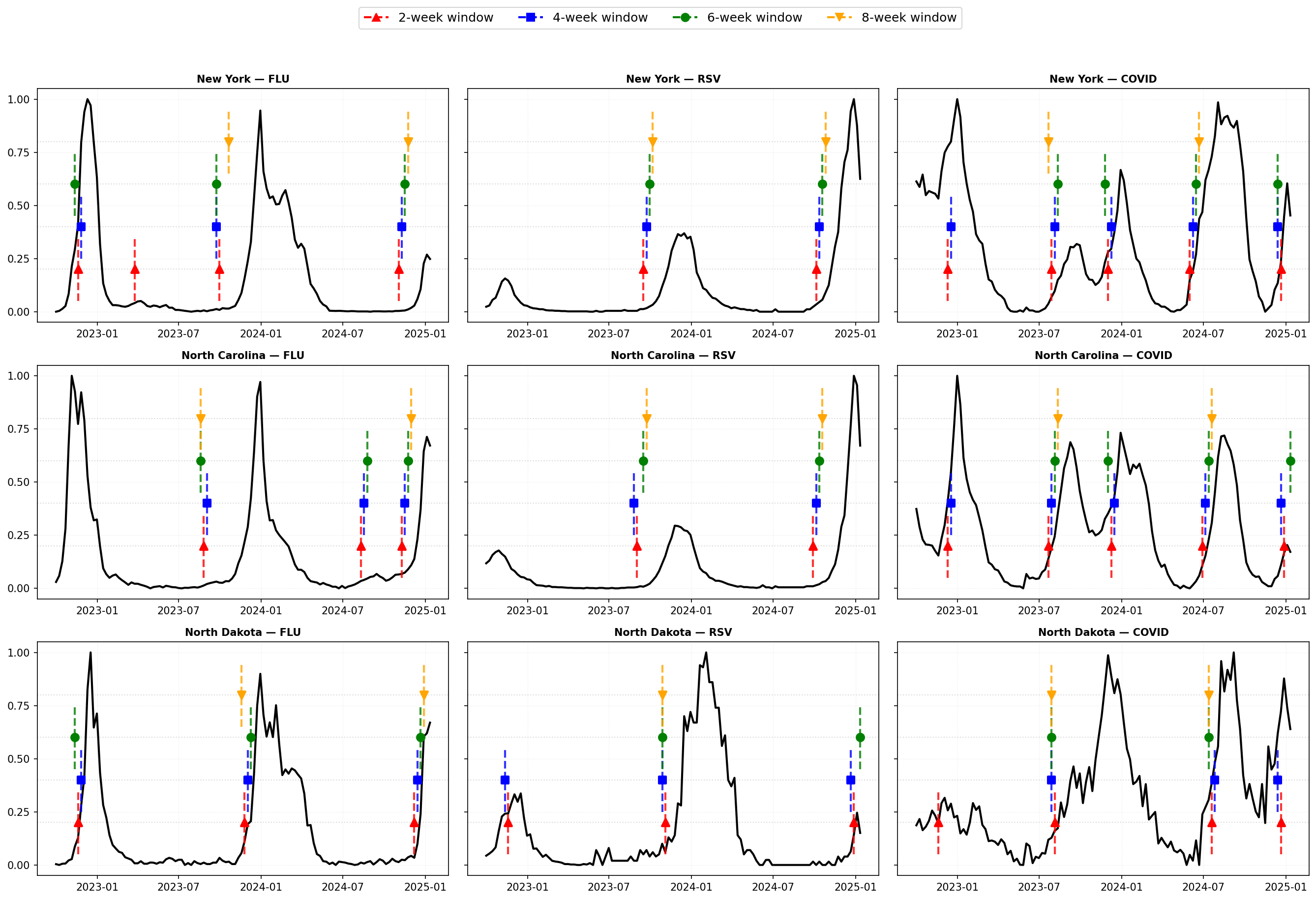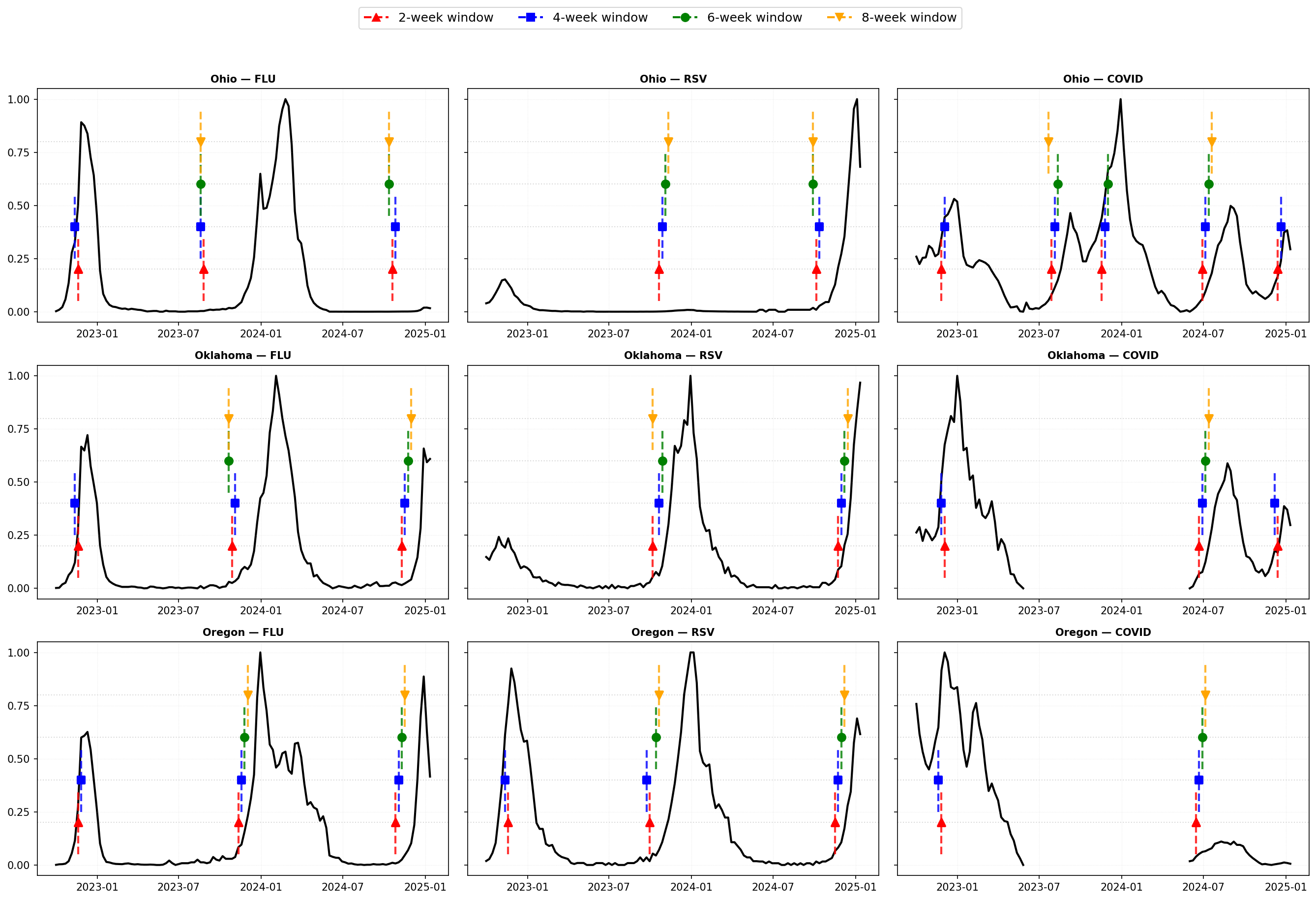 |
| --- |
| **Supplementary Figure 23**: **Sensitivity Analysis Results for Onset Detection Threshold for Predictors (New York, North Carolina, North Dakota, Ohio, Oklahoma, Oregon)**. Reducing the window size below 6 weeks results in increases in false positive rate, while increasing the window size to 8 weeks misses outbreaks. |

| 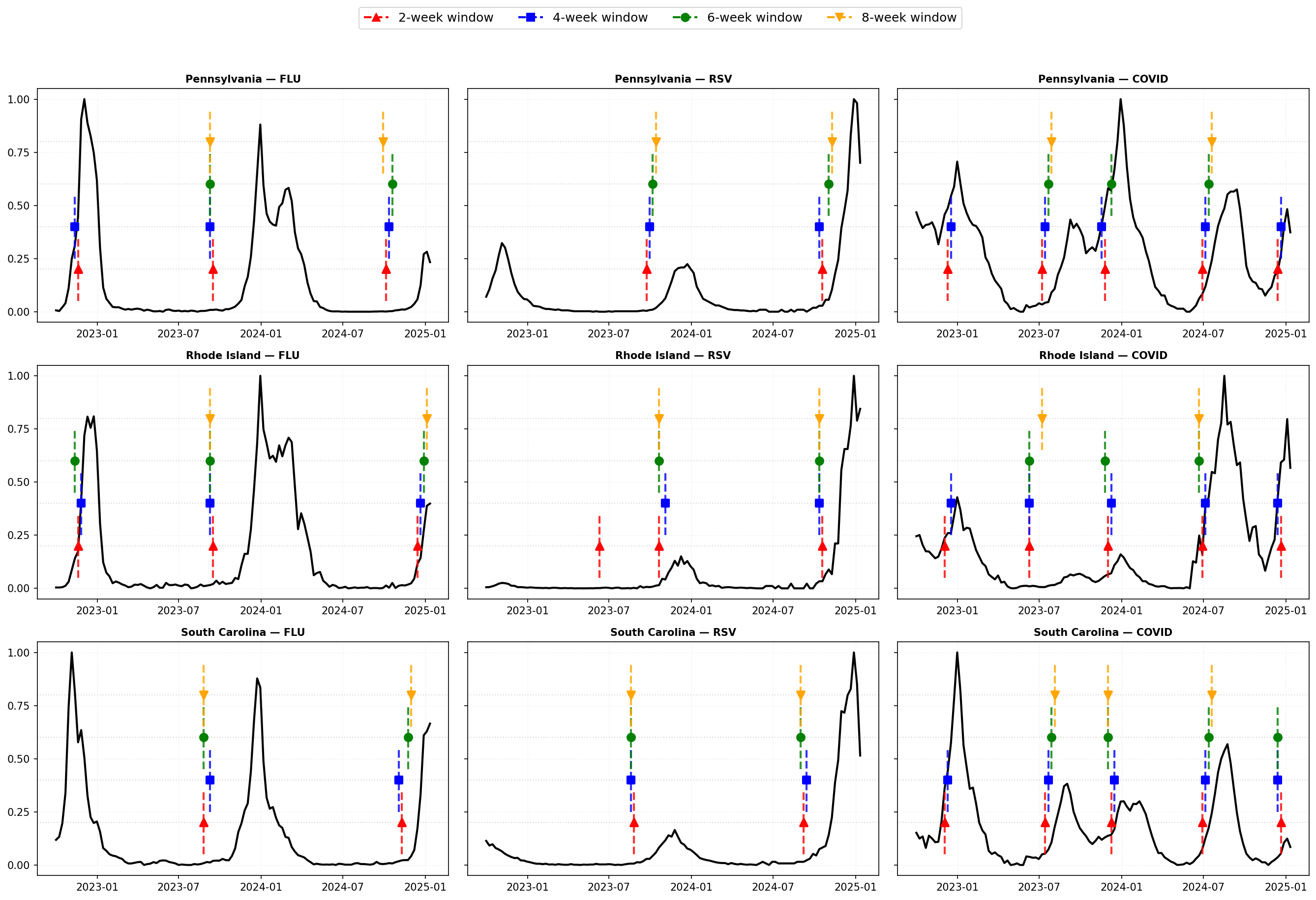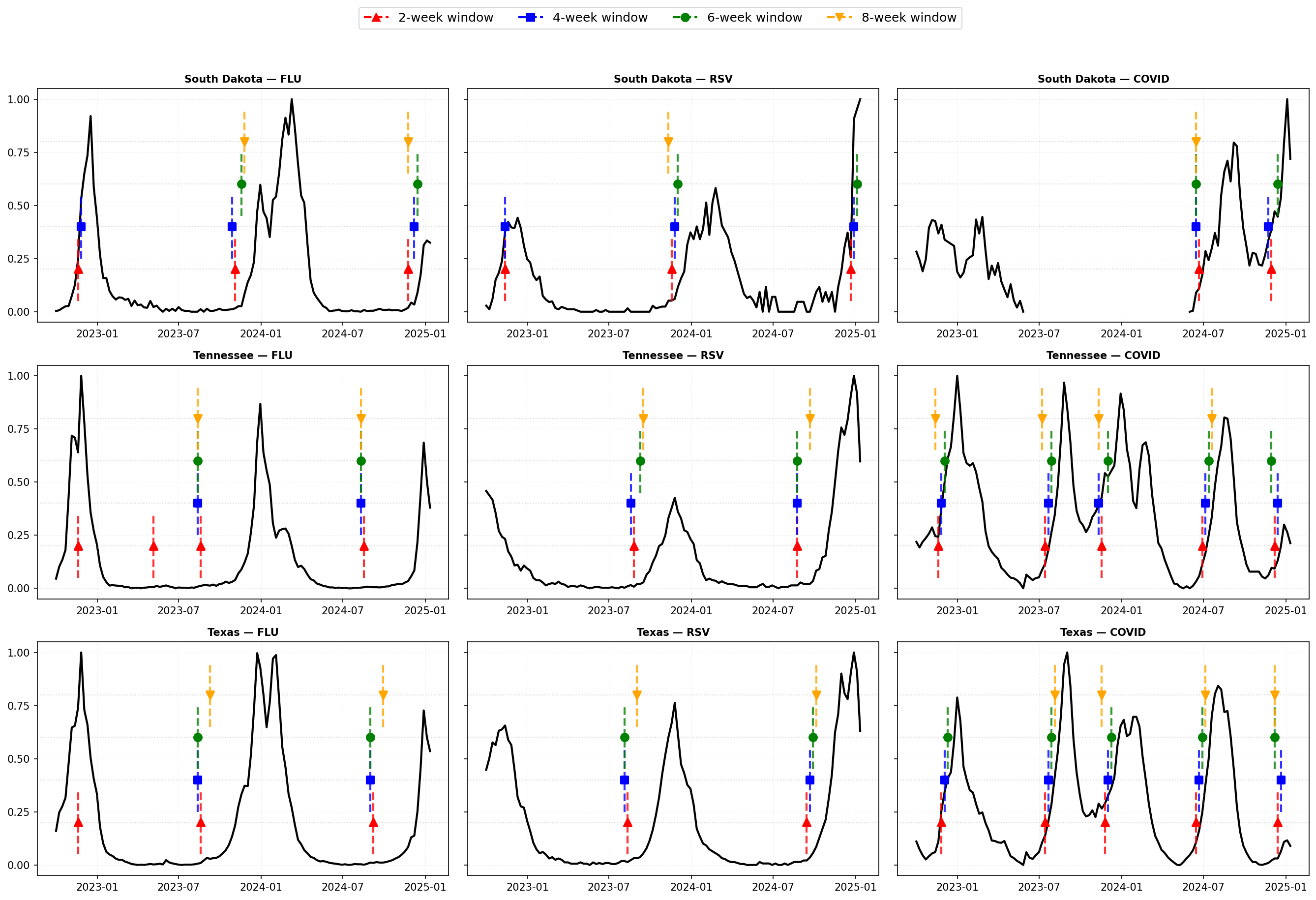 |
| --- |
| **Supplementary Figure 24**: **Sensitivity Analysis Results for Onset Detection Threshold for Predictors (Pennsylvania, Rhode Island, South Carolina, South Dakota, Tennessee, Texas)**. Reducing the window size below 6 weeks results in increases in false positive rate, while increasing the window size to 8 weeks misses outbreaks. |

| 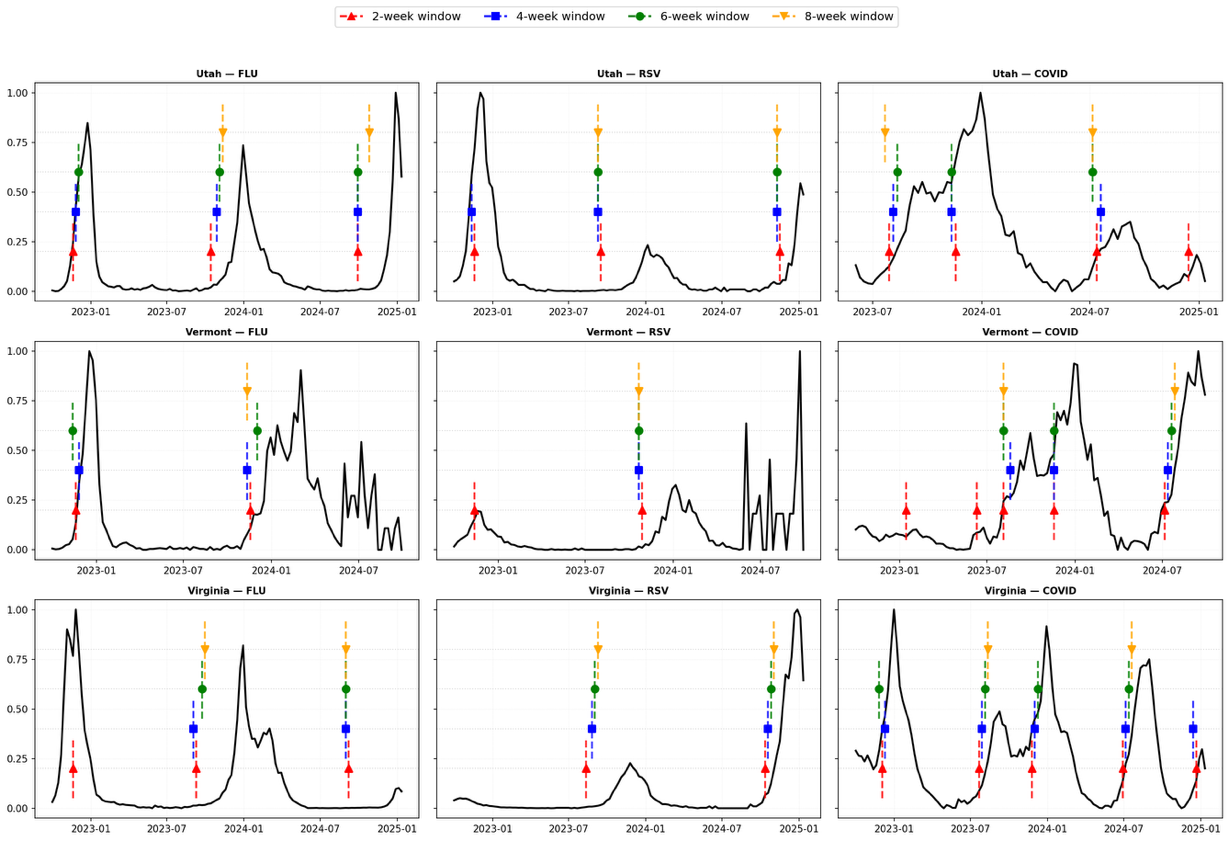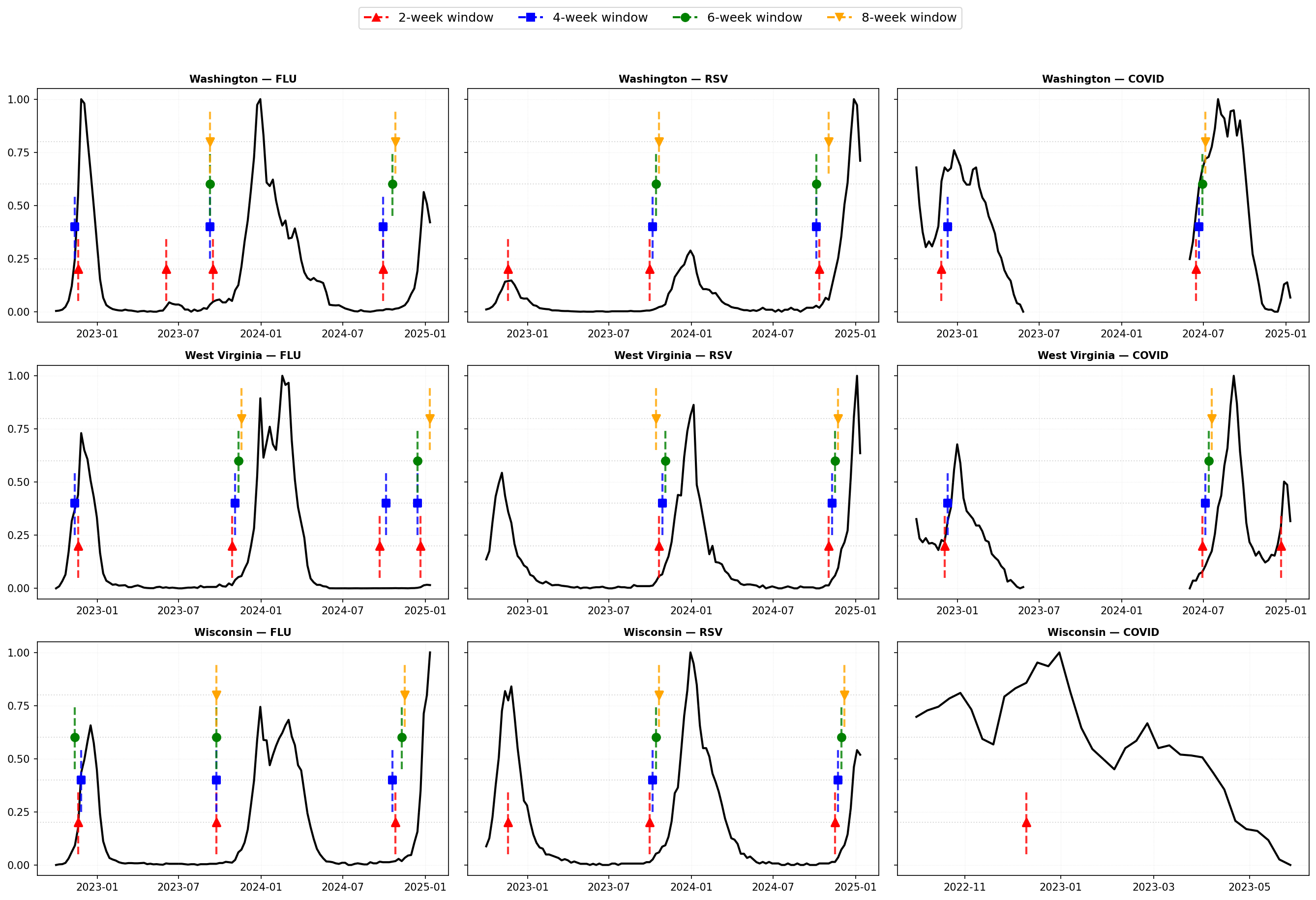 |
| --- |
| **Supplementary Figure 26**: **Sensitivity Analysis Results for Onset Detection Threshold for Predictors (Utah, Vermont, Virginia, Washington, West Virginia, Wisconsin)**. Reducing the window size below 6 weeks results in increases in false positive rate, while increasing the window size to 8 weeks misses outbreaks. |

| 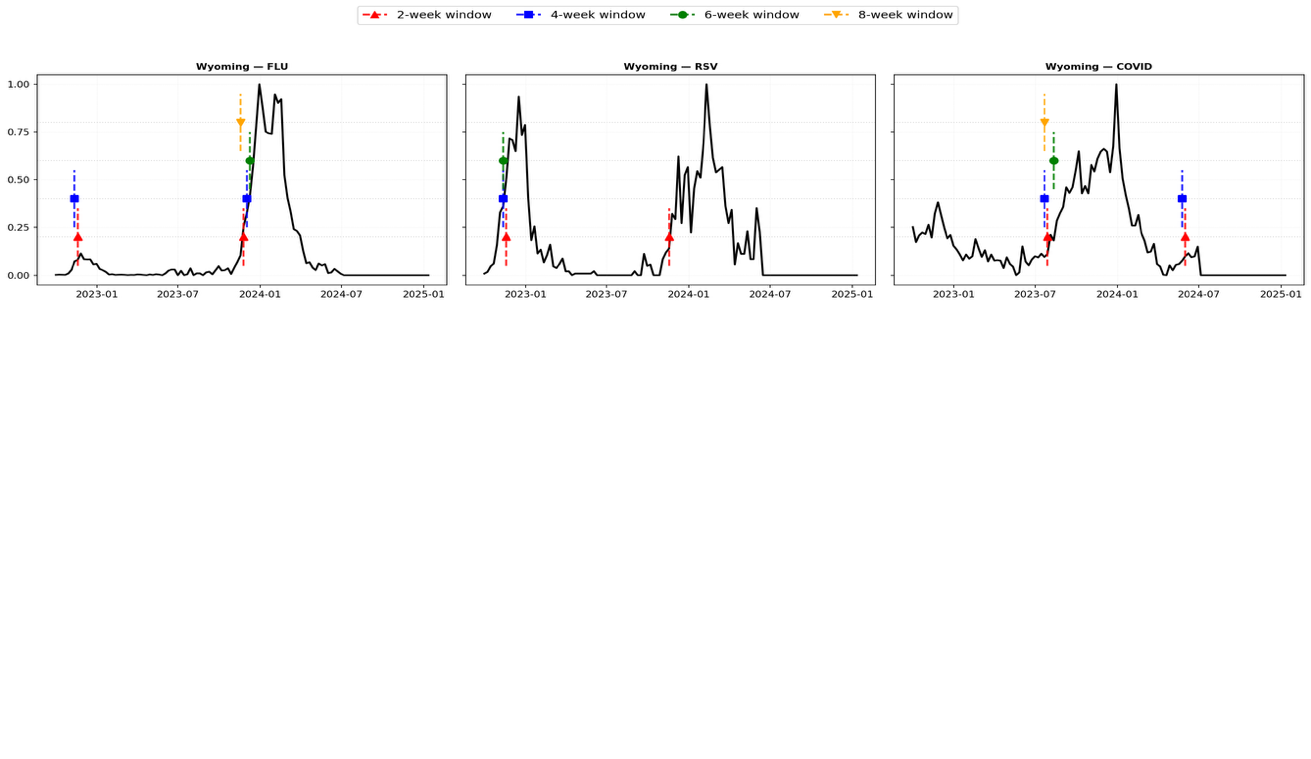 |
| --- |
| **Supplementary Figure 27**: **Sensitivity Analysis Results for Onset Detection Threshold for Predictors (Wyoming)**. Reducing the window size below 6 weeks results in increases in false positive rate, while increasing the window size to 8 weeks misses outbreaks. |

| **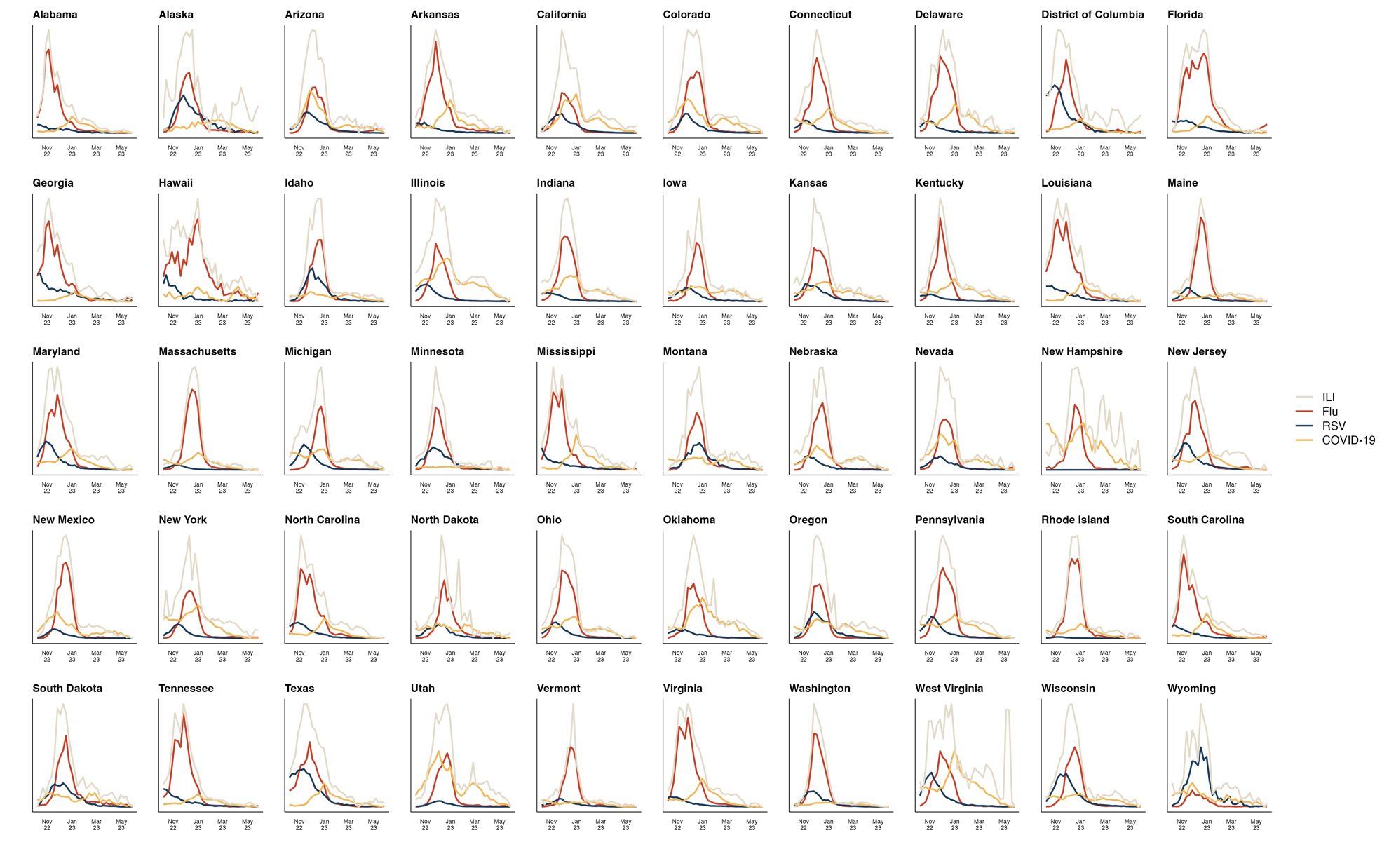** |
| --- |
| Supplementary Figure 28: Timing of Respiratory Epidemics in the United States for the 2022-2023 ILI season. Abbreviations - COVID-19: coronavirus disease 2019; flu: influenza; ILI: influenza-like illness; RSV: respiratory syncytial virus |

| **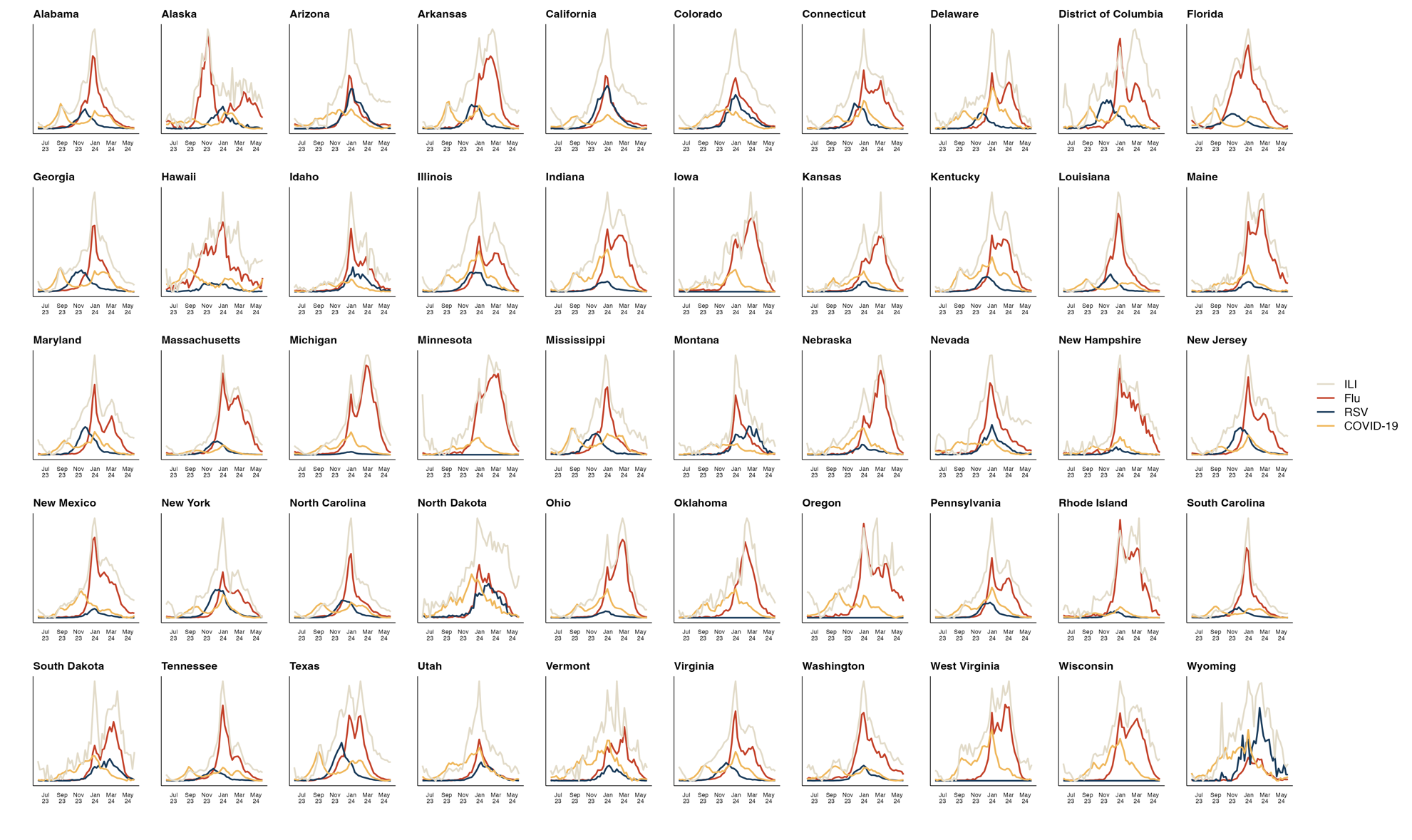** |
| --- |
| Supplementary Figure 29: Timing of Respiratory Epidemics in the United States for the 2023-2024 ILI season. Abbreviations - COVID-19: coronavirus disease 2019; flu: influenza; ILI: influenza-like illness; RSV: respiratory syncytial virus |

| **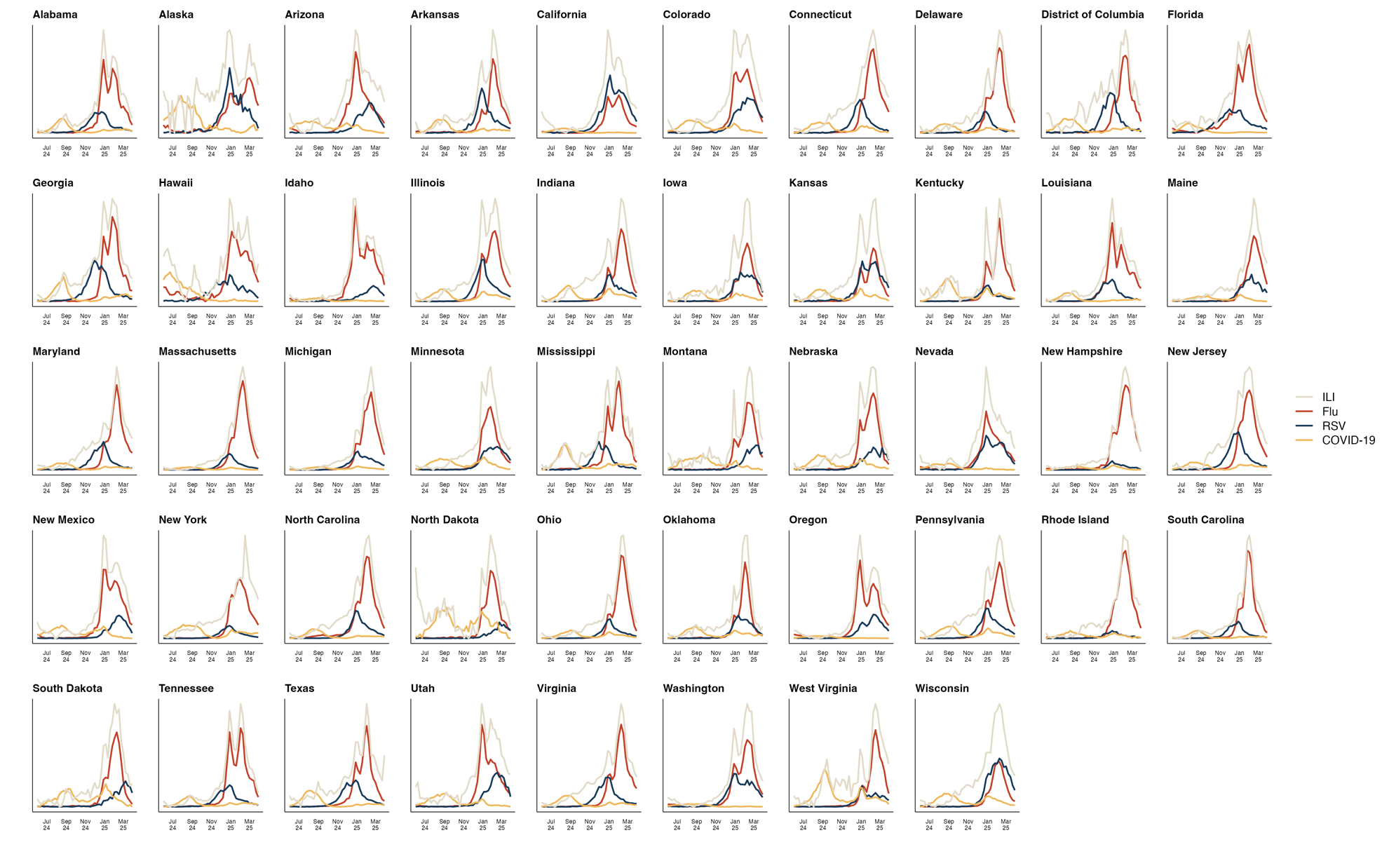** |
| --- |
| Supplementary Figure 30: Timing of Respiratory Epidemics in the United States for the 2024-2025 ILI season. Data from Wyoming and Vermont were not supplied to either NSSP or ILINet and were excluded from the analysis. Abbreviations - COVID-19: coronavirus disease 2019; Flu: influenza; ILI: influenza-like illness; RSV: respiratory syncytial virus |

| **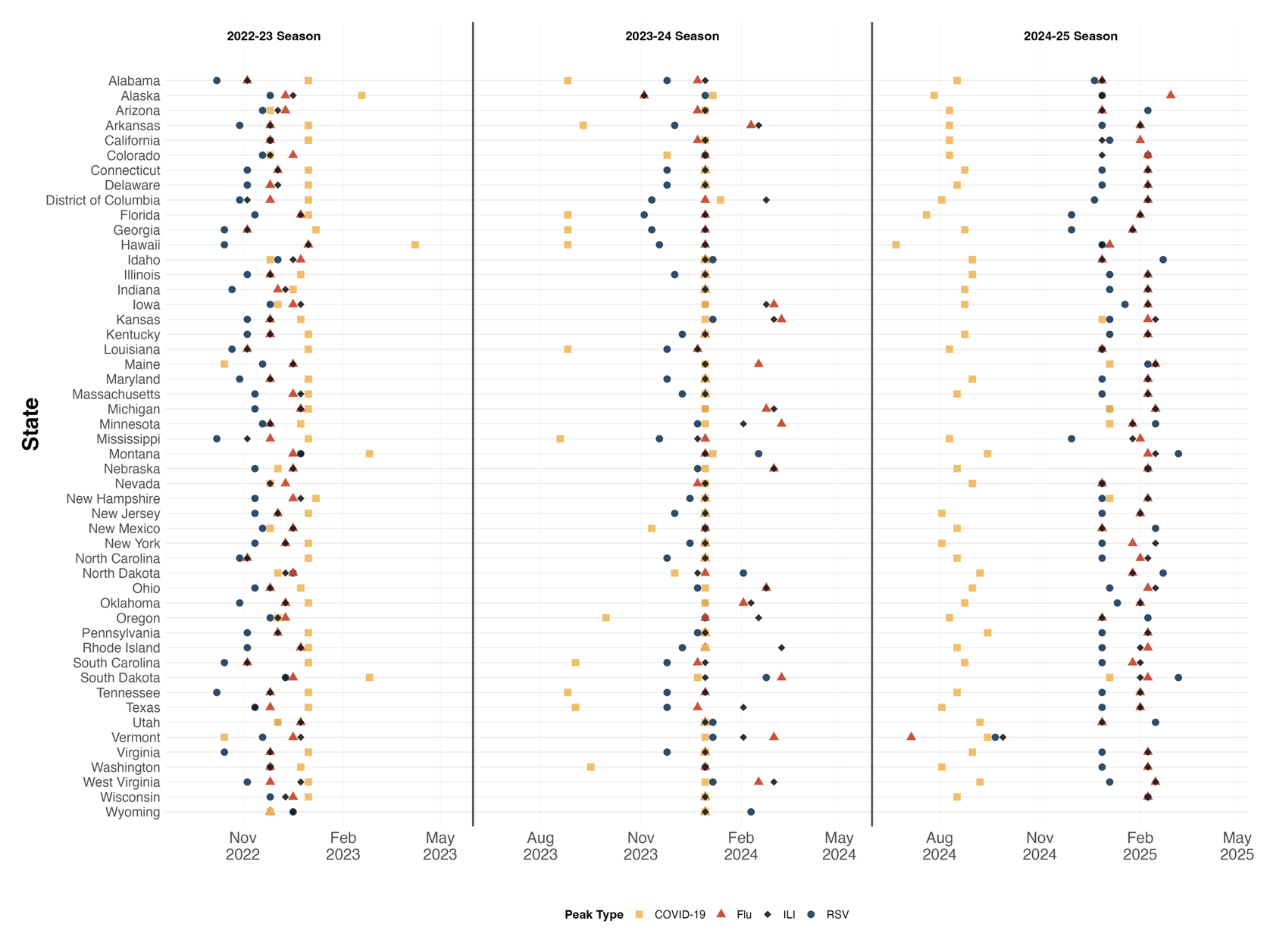** |
| --- |
| Supplementary Figure 31: State-level timelines of epidemic peaks based on the regression method for the 22-23, 23-24, and 24-25 ILI seasons in the United States. In general, RSV peaks occurred before flu peaks, while COVID-19 demonstrated irregular seasonality across the 3 years. |

### Supplementary Figures 32-37: Lagged Correlation Analysis

To quantify the temporal relationship between epidemics of influenza, RSV, and COVID-19 and confirm what we observed in our regression modeling results, we deployed a lagged correlation analysis as an alternative to our early warning system method. We first evaluated the time differences between peaks of epidemic activity by computing correlations between the trend of influenza ED visits and lagged trends of both RSV and COVID-19 ED visits (**Supplementary Figures 32-34**). We found that the influenza signal was highly correlated (Pearson correlation ≥ 0.6) with either concurrent RSV, lag-1 RSV, or lag-2 RSV (RSV shifted one or two weeks prior) in 49 out of 50 regions (98.0%) in the 22-23 season, 46 out of 50 regions (92.0%) in the 23-24 season, and 41 out of 48 regions (85.4%) in the 24-25 season. Based on this finding, we estimate that there is up to a 3 week delay between peak RSV volume and peak influenza volume. To validate these findings, we next performed a robustness check by conducting a lagged correlation analysis comparing a fixed influenza signal and COVID-19 signals shifted forward and backward in time (**Supplementary Figures 35-37**). Results of this check show the same inconsistent seasonality that we observed from the regression modeling results – we observe three different correlation patterns across the 3 analyzed seasons, indicating no regularity in the time difference between peaks of influenza volume and peaks of COVID-19 volume.

| **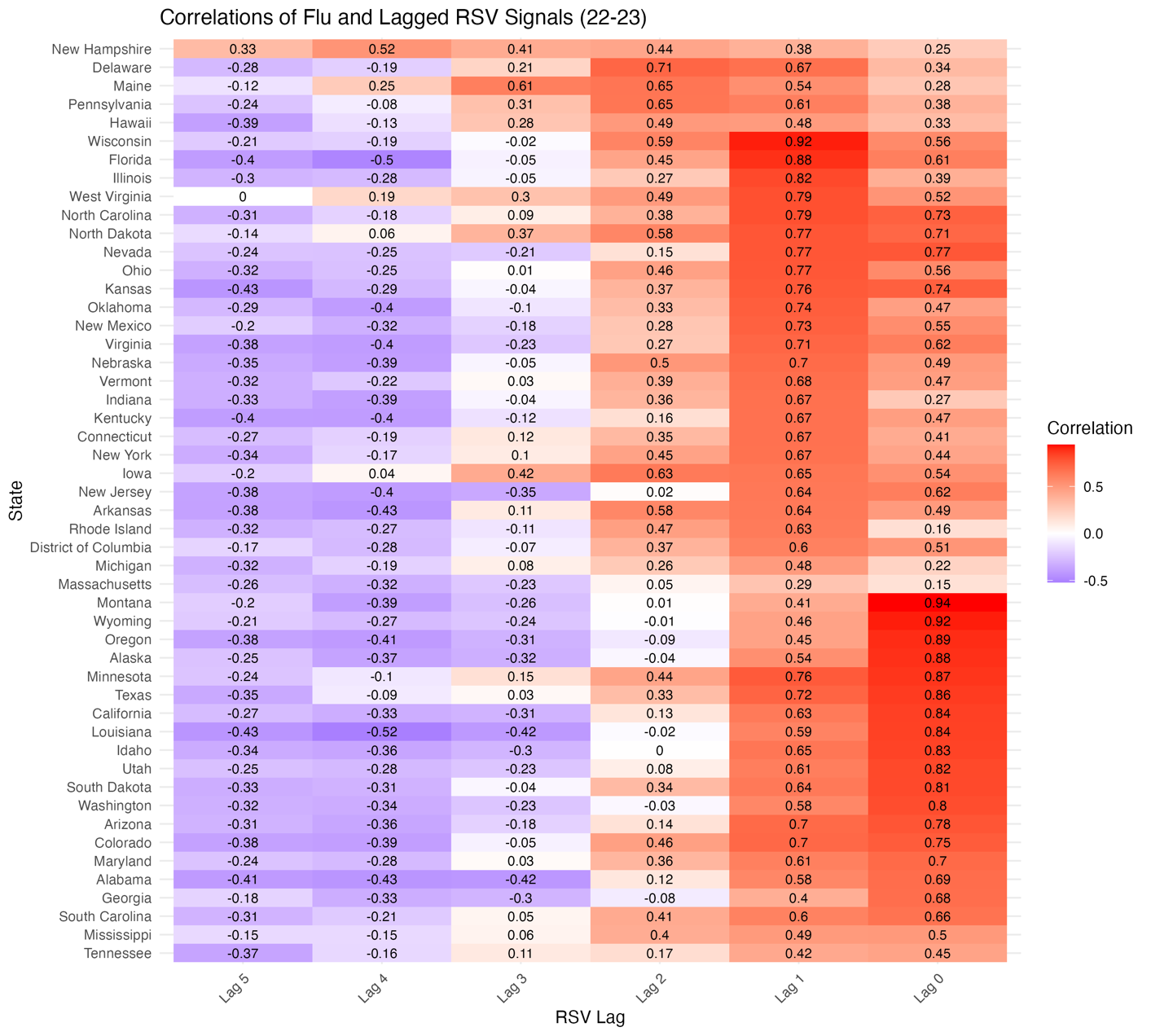** |
| --- |
| **Supplementary Figure 32: Correlations between influenza ED visits and lagged RSV ED visits in the 2022-23 ILI season.** The strongest correlations between influenza and RSV ED visits in the 22-23 season were in the lag-1 and lag-0 columns, implying the average deviation between signal peaks was between 0 and 1 weeks. The ordering of the state rows groups states together by the time lag where the flu and lagged RSV signals were the most correlated, highlighting the block of states in the lag-1 and lag-0 columns. |

| **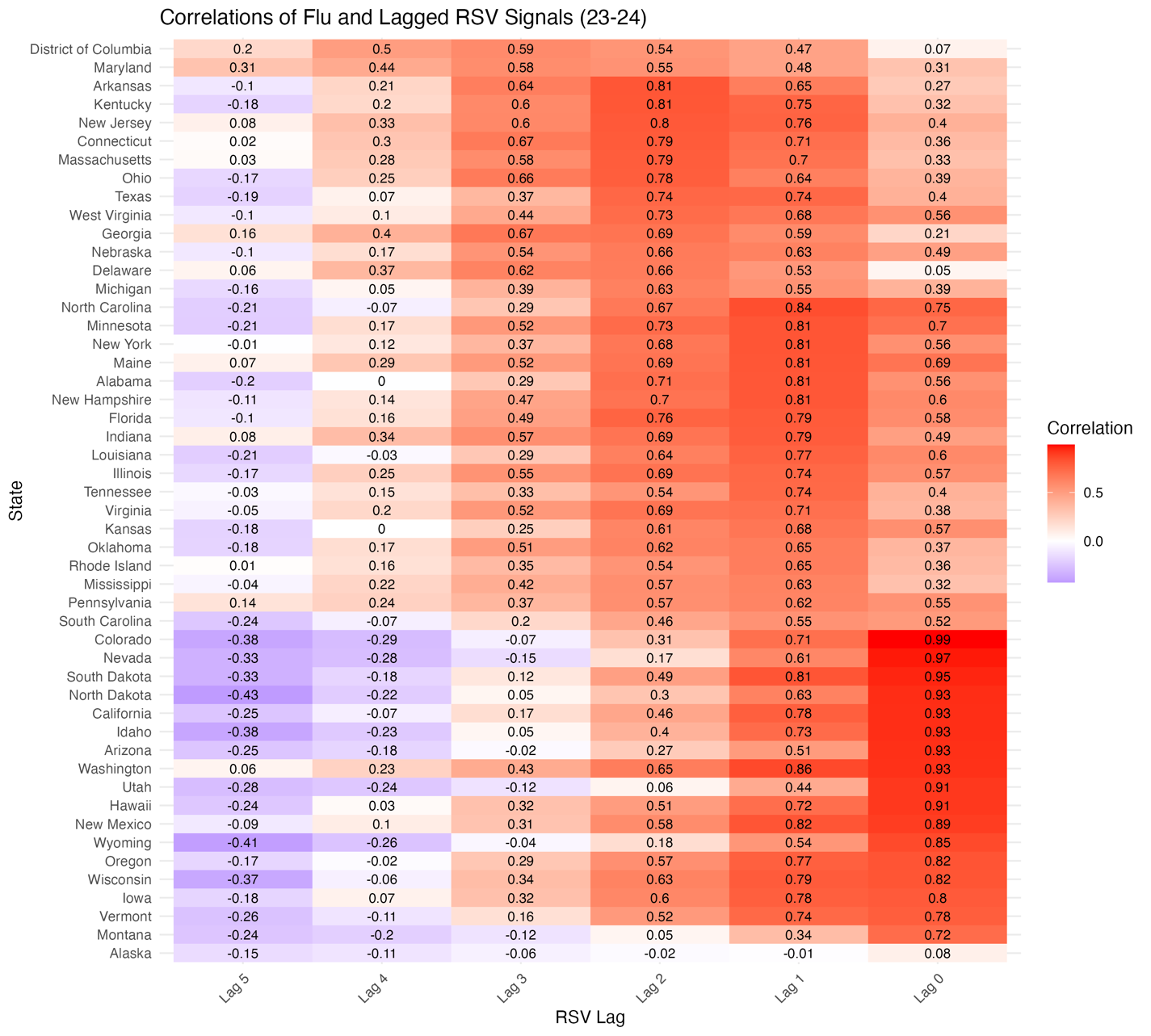** |
| --- |
| **Supplementary Figure 33: Correlations between influenza ED visits and lagged RSV ED visits in the 2023-24 ILI season.** The strongest correlations between influenza and RSV ED visits in the 23-24 season were in the lag-1 and lag-0 columns, implying the average deviation between signal peaks was between 0 and 1 weeks. The ordering of the state rows groups states together by the time lag where the flu and lagged RSV signals were the most correlated. |

| **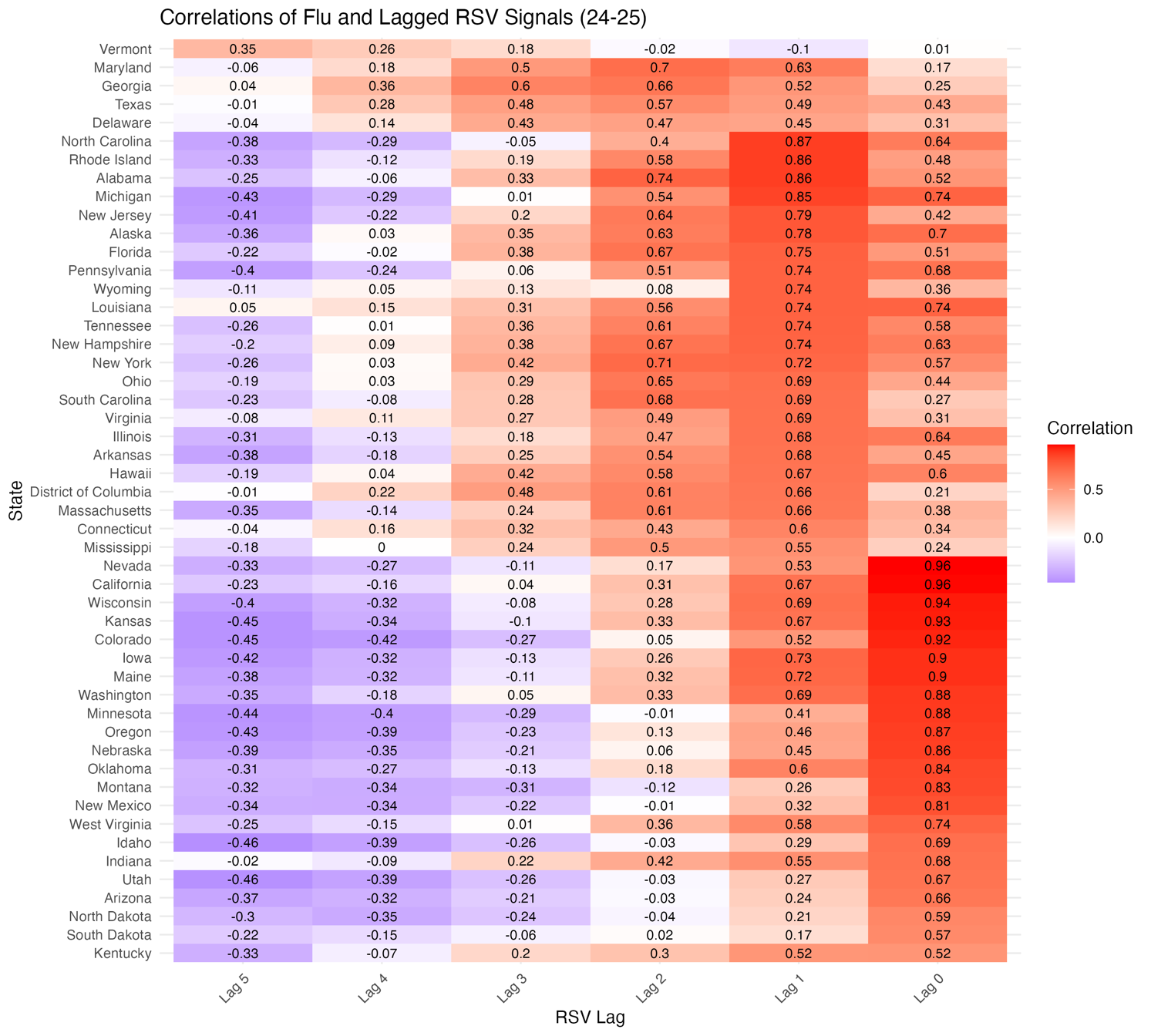** |
| --- |
| **Supplementary Figure 34: Correlations between influenza ED visits and lagged RSV ED visits in the 2024-25 ILI season.** The strongest correlations between influenza and RSV ED visits in the 24-25 season were in the lag-1 and lag-0 columns, implying the average deviation between signal peaks was between 0 and 1 weeks. The ordering of the state rows groups states together by the time lag where the flu and lagged RSV signals were the most correlated. |

| **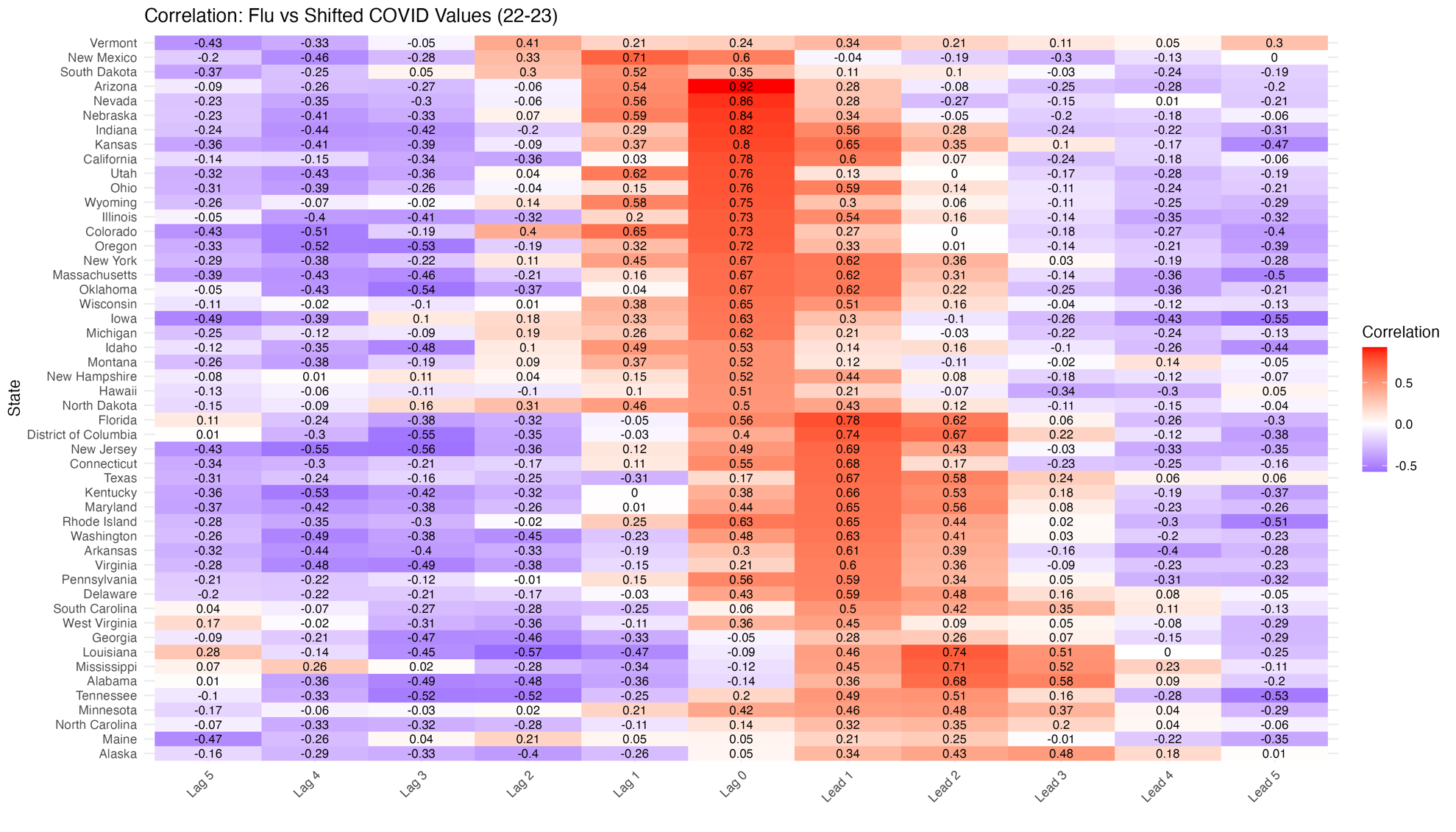** |
| --- |
| **Supplementary Figure 35: Correlations between influenza ED visits and time-shifted COVID-19 ED visits in the 2022-23 ILI season.** The ordering of the state rows groups states together by the time lag where the flu and lagged COVID signals were the most correlated. |

| **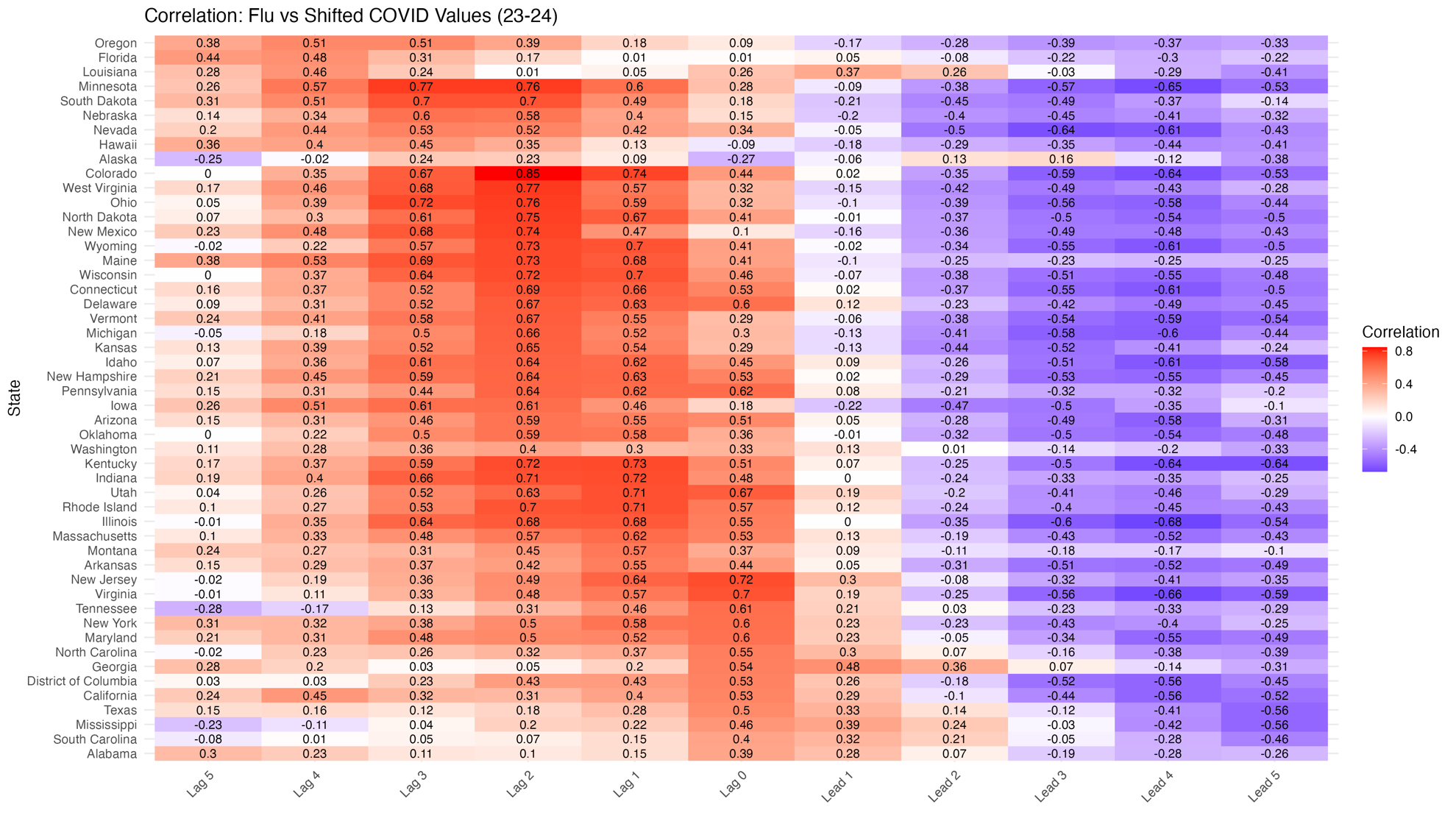** |
| --- |
| **Supplementary Figure 36: Correlations between influenza ED visits and time-shifted COVID-19 ED visits in the 2023-24 ILI season.** The ordering of the state rows groups states together by the time lag where the flu and lagged COVID signals were the most correlated. |

| **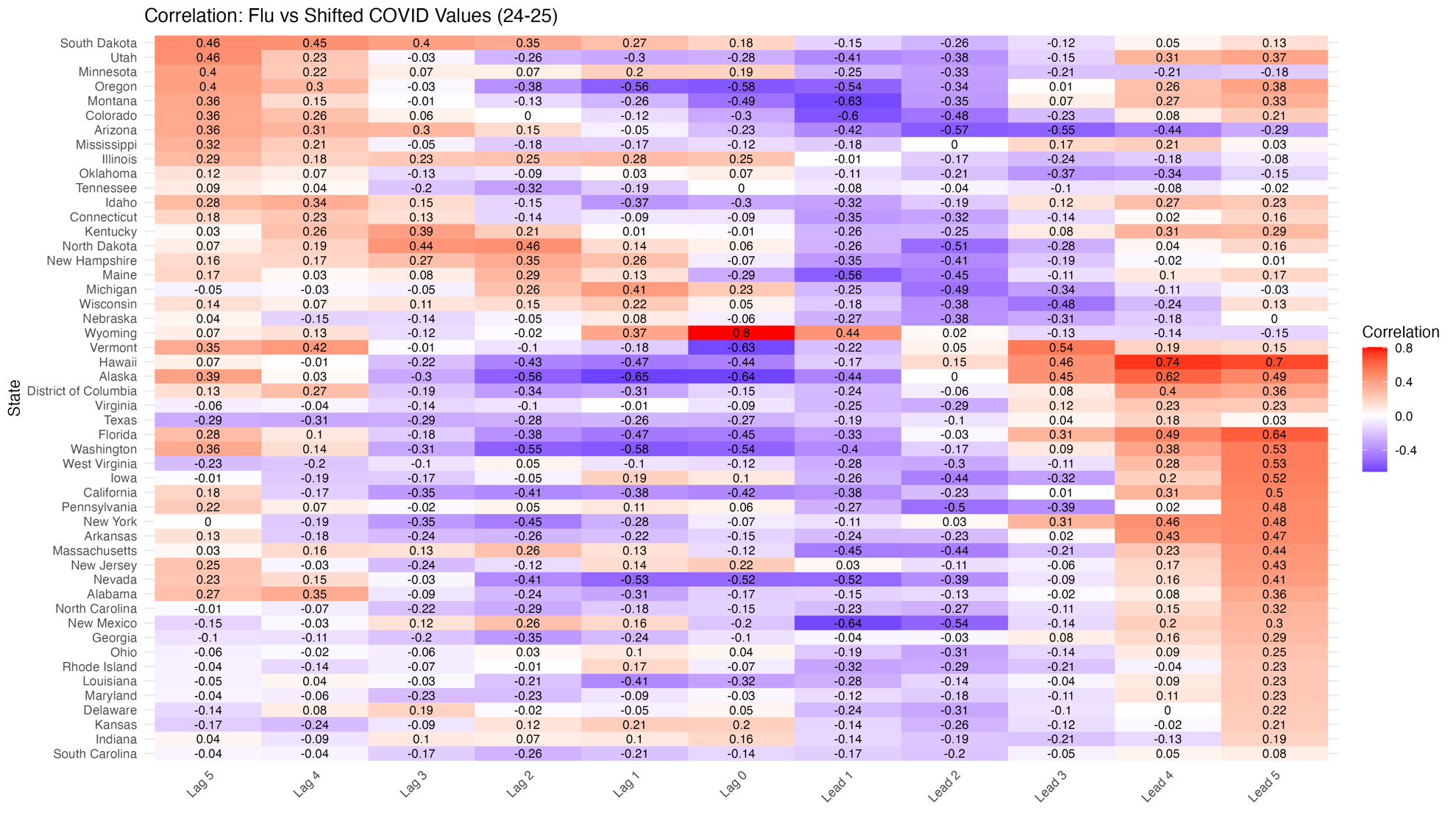** |
| --- |
| **Supplementary Figure 37: Correlations between influenza ED visits and time-shifted COVID-19 ED visits in the 2024-25 ILI season.** The ordering of the state rows groups states together by the time lag where the flu and lagged COVID signals were the most correlated. |

| **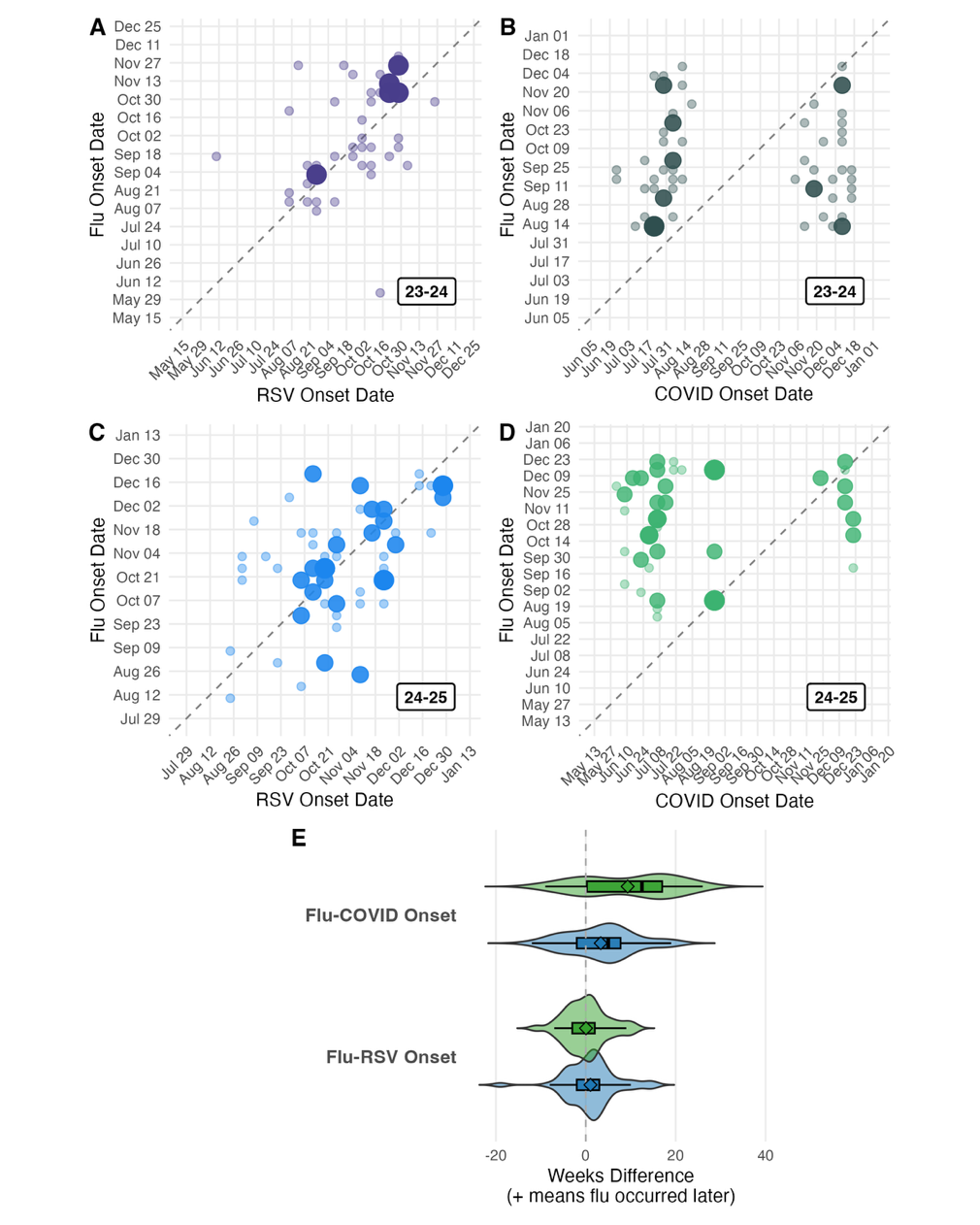** |
| --- |
| Supplementary Figure 38: Anomaly detection methods show no clear precedence of RSV or COVID-19 before influenza when comparing epidemic onsets. Points above the diagonal line indicate that the disease on the *y*-axis (influenza) peaks before the disease on the *x*-axis (either RSV or COVID-19). Larger points indicate that peaks in multiple states occurred during the same week. |

| **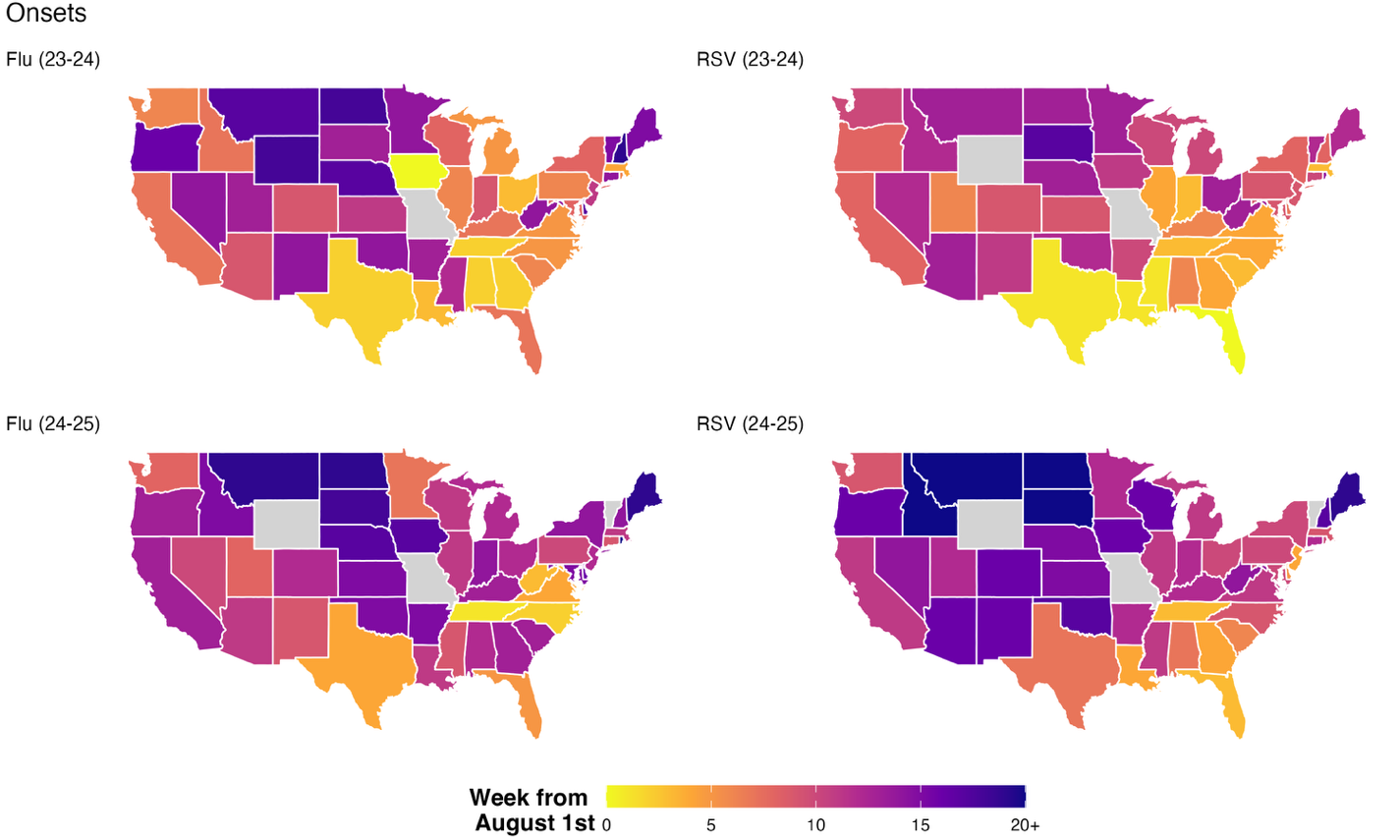** |
| --- |
| Supplementary Figure 39: Anomaly detection methods indicate influenza and RSV epidemic onsets spread in a southeast to northwest direction the 2023-24 and 2024-25 ILI seasons**.** The colors represent the number of weeks after August 1^st^ of the respective season for the anomaly detection method to identify the onset of an epidemic. States that are greyed out did not report data during the corresponding seasons. |

| **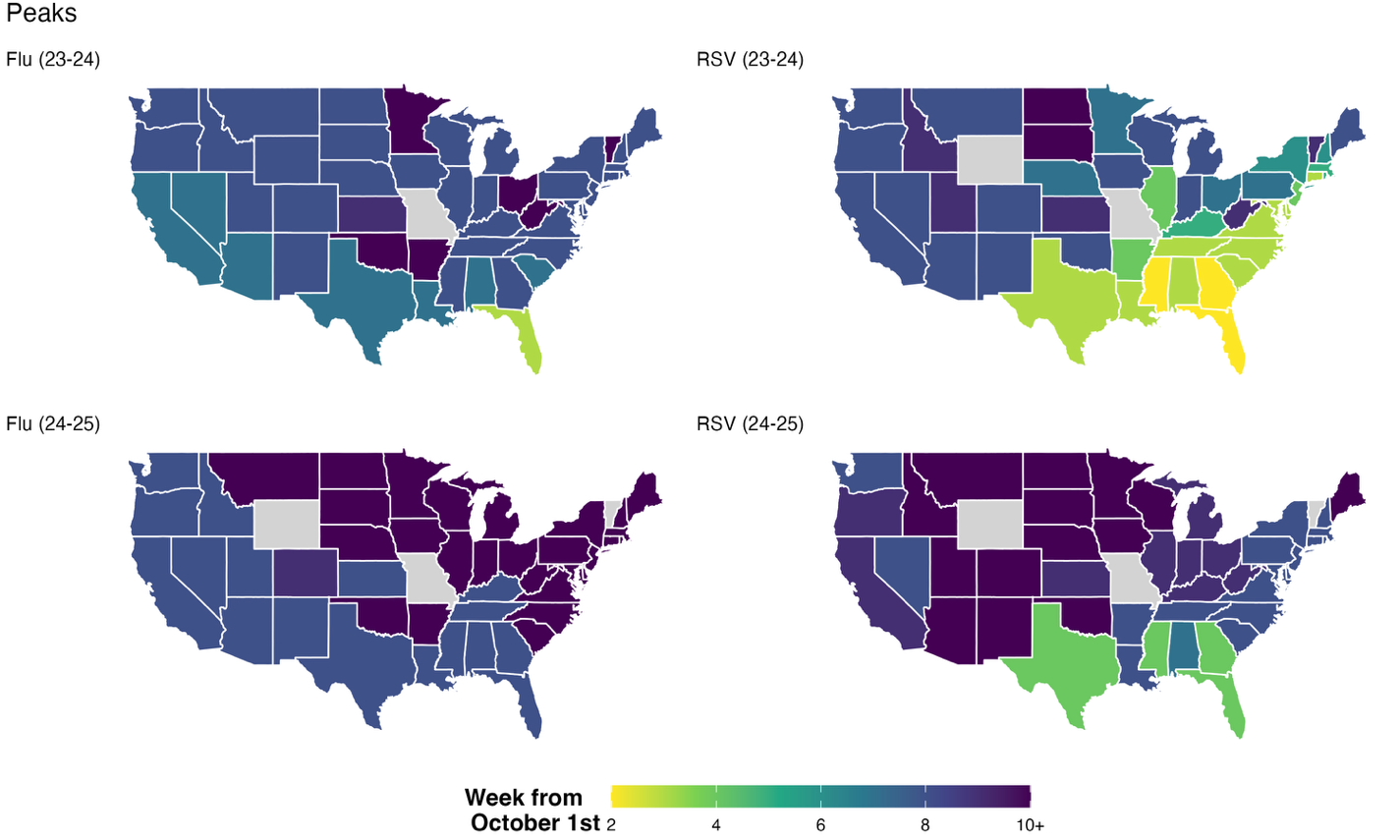** |
| --- |
| Supplementary Figure 40: Anomaly detection methods indicate influenza and RSV epidemic peak spread in a southeast to northwest direction the 2023-24 and 2024-25 ILI seasons. The colors represent the number of weeks after October 1^st^ of the respective seasons for the anomaly detection method to identify an epidemic peak. States that are greyed out did not report data during the corresponding seasons. |
